# Supplementary material for: What is the prevalence of COVID-19 detection by PCR among deceased individuals in Lusaka, Zambia? A postmortem surveillance study
Source: BMJ Open. 2022 Dec 6;12(12):e066763. doi: 10.1136/bmjopen-2022-066763 (PMC9729848; doi:10.1136/bmjopen-2022-066763)
Supplement: Supplementary data [file bmjopen-2022-066763supp001.pdf]

SUPPLEMENTARY DATA

Table S1. Proportion of the deceased who had been tested antemortem, by setting

	Facility	Community	All deaths
Tested antemortem	47.4% (272)	1.8% (10)	282
Not tested antemortem	52.6% (302)	97.2% (534)	836
Total	574	544	1,118

Note: We have elected not to conduct a detailed analysis of the concordance between the off study antemortem (clinical care) and postmortem (research) testing. Our team had no role in how those off study samples were collected, what swabs were used, how staff were trained, what test kits were used over time, what quality control measures were used, and so forth. Hence, we have no provenance over the non-study data and do not vouch for their accuracy.

With that caveat, we identified 35 individuals for whom a positive test result was recorded or reported (25 facility and 10 community deaths). Obviously, this is far fewer than what we observed in our postmortem testing. For the reasons outlined above, we can only hypothesize explanations for this discrepancy. Yet, the simplest explanation is plausible: most of the clinical samples were evaluated using rapid antigen kits, which are far less sensitive than PCR.

Please also note that this Table does not account for the different enrollment ratios between the facility and community deaths, which will under-represent the community deaths by three-fold.

Table S2. Proportion of the deceased who tested positive for CV19 by study month

	Ct>=40 (Neg)	Ct<40 (Pos)	Total	Ct>=40 (Neg)	Ct<40 (Pos)	Total
**Month**	**Unweighted**			**Weighted**		
January	77<br>47.8%	84<br>52.2%	161	108.1<br>49.4%	110.8<br>50.6%	218.8
February	118<br>57.0%	89<br>43.0%	207	118.2<br>57.1%	88.9<br>42.9%	207.2
March	189<br>77.5%	55<br>22.5%	244	173.1<br>76.7%	52.7<br>23.4%	225.8
April	216<br>91.5%	20<br>8.5%	236	176.8<br>92.2%	14.9<br>7.8%	191.7
May	154<br>89.0%	19<br>11.0%	173	154.4<br>92.1%	13.3<br>7.9%	167.7
June	37<br>38.1%	60<br>61.9%	97	29.3<br>27.4%	77.7<br>72.6%	107.0
Total	791<br>70.8%	327<br>29.2%	1,118	759.9<br>68.0%	358.4<br>32.0%	1,118.3<br>100.0%

Row results are N (Top) and % (Bottom) in each cell.

Table S3. Clinical presentations of pediatric CV19 positive deaths

Table S3a. Clinical presentation of COVID-19+ facility deaths in children aged 0-19 years

No.	Age in years	Respiratory Symptoms	Gastrointestinal Symptoms	Fever	Cause of Death*
1	<1	difficulty breathing	diarrhea	X	sepsis
2	<1	cough		X	pneumonia
3	<1	cough, difficulty breathing, shortness of breath	diarrhea, vomiting	X	pneumonia
4	<1	difficulty breathing			sepsis
5	<1	cough, difficulty breathing		X	COVID-19 pneumonia
6	<1	difficulty breathing			sepsis
7	<1	difficulty breathing			pneumonia
8	<1	difficulty breathing			sepsis
9	<1	runny nose			sepsis
10	<1	difficulty breathing			sepsis
11	<1	cough	vomiting		sepsis
12	<1	difficulty breathing		X	sepsis
13	<1		vomiting	X	sepsis
14	<1			X	sepsis
15	<1			X	sepsis
16	<1				sepsis
17	<1	difficulty breathing, shortness of breath		X	sepsis
18	<1			X	sepsis
19	<1			X	sepsis
20	<1	cough, difficulty breathing		X	sepsis
21	<1	difficulty breathing			sepsis
22	<1				hypothermia
23	<1			X	sepsis
24	<1	difficulty breathing			pneumonia
25	<1	cough, difficulty breathing		X	pneumonia
26	1-5	shortness of breath		X	sepsis
27	1-5		diarrhea, vomiting		diarrhea
28	1-5				dehydration
29	1-5		diarrhea	X	malnutrition
30	1-5		diarrhea	X	sepsis
31	1-5			X	respiratory failure
32	6-10				diarrhea
33	6-10	difficulty breathing			sepsis
34	11-15				intracranial abscess

* Cause of death from the official death certificate completed by the UTH medical examiner.

Table S3b. Clinical presentation of COVID-19+ community deaths in children aged 0-19 years

No.	Age in years	Respiratory Symptoms	Gastrointestinal Symptoms	Fever
1	<1	difficulty breathing		
2	<1	cough		X
3	<1	cough, difficulty breathing	vomiting	
4	<1		vomiting	
5	<1			X
6	<1		diarrhea, vomiting	X
7	<1	cough, difficulty breathing, fast breathing, shortness of breath		X
8	<1			
9	<1			
10	<1	cough, fast breathing		X
11	<1		diarrhea, vomiting	X
12	<1	cough, difficulty breathing	diarrhea	X
13	1-5	cough		X
14	1-5		diarrhea, vomiting	
15	1-5		diarrhea, vomiting	X
16	1-5		diarrhea	
17	1-5		diarrhea, vomiting	X
18	1-5			
19	1-5			X
20	1-5		diarrhea	
21	1-5	cough	diarrhea	
22	1-5			
23	1-5	cough, difficulty breathing		
24	6-10			
25	6-10		diarrhea	
26	16-19	difficulty breathing		
27	16-19	cough, difficulty breathing		
28	16-19			X
29	16-19			
30	16-19			

We do not include a cause of death for the community deaths. A death certificate is still issued for each case, but these are not based on information from medical providers and are not considered to be accurate.

Supplementary Table S4. The relationship between age and PCR results and viral load as measured through cycle threshold values (Ct).

Age at death (years)	Ct≥40 (Neg)	Weaker (30≤Ct<40)	Strong-est (Ct<30)	Ratio of Weaker to Strongest	Ct≥40 (Neg)	Weaker (30≤Ct<40)	Strong-est (Ct<30)	Ratio of Weaker to Strongest
	**Unweighted**				**Weighted**			
0-19	304<br>82.8	54<br>14.7	9<br>2.5	6.0	203.4<br>79.4	45.8<br>17.9	6.9<br>2.7	6.6
20-39	158<br>66.7	55<br>23.2	24<br>10.1	2.3	184.2<br>67.2	62.3<br>22.7	27.7<br>10.1	2.3
40-59	171<br>74.0	36<br>15.6	24<br>10.4	1.7	190.1<br>75.2	39.4<br>15.6	23.4<br>9.3	1.7
60-79	107<br>58.5	45<br>24.6	31<br>16.9	1.4	119.8<br>56.1	54.8<br>25.7	38.9<br>18.2	1.4
80-99	42<br>52.5	19<br>23.8	19<br>23.8	1.0	55.4<br>52.0	26.1<br>24.5	25.0<br>23.5	1.0
100+	0<br>0.0	1<br>50.0	1<br>50.0	1.0	0<br>0.0	1.6<br>50.0	1.6<br>50.0	1.0
Total	782	210	108	1.9	752.9	230.0	123.5	1.9

*4 had missing age data and 12 had no Ct value (tested antemortem with rapid test).

Row results are N (Top) and % (Bottom) in each cell.

Within each age strata we plot the proportion of decedents with a ‘negative’ test, defined as Ct >40, or a positive test, stratified by Ct strata. Ct is the inverse of the viral load, hence low Ct equals a stronger PCR result and a higher vial load. What we observe is an age dependent effect on the ratio of weaker to strongest PCR strata. Among younger individuals ≤19 years, the ratio of weaker to strongest PCR results was 6.6:1, after weighting. This ratio declines as a function of increasing age. If we assume that viral load is reflective of pathology, then the higher ratios seen in the youngest group indicate a greater proportion of cases where COVID-19 was a co-incidental finding, whereas those in the older age groups, indicate that COVID-19 was more likely to have been a causal factor in those deaths.

Figure S1. Comparison of death by age distributions for the total deceased cohort, the enrolled cohort, and the CV19 positive enrolled cohort.

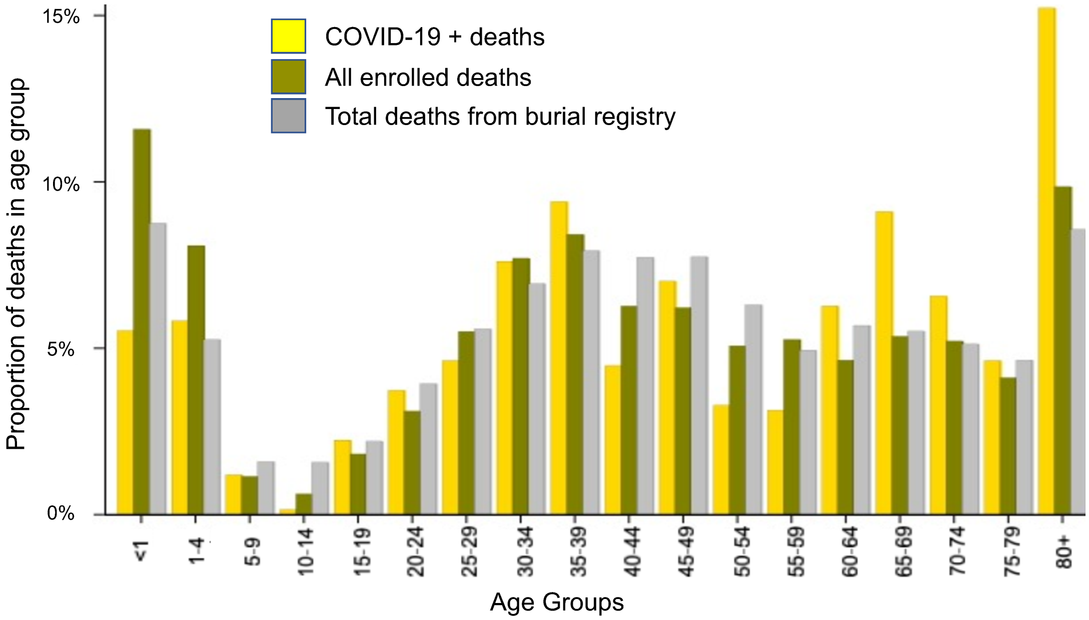

Supplementary Figure S1. Comparison of death by age distributions for the total deceased cohort, the enrolled cohort, and the COVID-19 positive enrolled cohort.

Data for the total death cohort were from Lusaka's burial registry logs maintained by the Zambian Ministry of Health. From each entry, we extracted data of death, age, and sex. We then extracted those deaths that corresponded to the periods of surveillance to capture the age by death distribution for the total cohort (enrolled and unenrolled). The second two sets correspond to the total enrolled cohort (N=1,118 person) and the subset who were COVID-19+ (N=327).

Figure S2. Distribution of cycle threshold values for PCR results targeting the N1 and N2 nucleocapsid proteins.

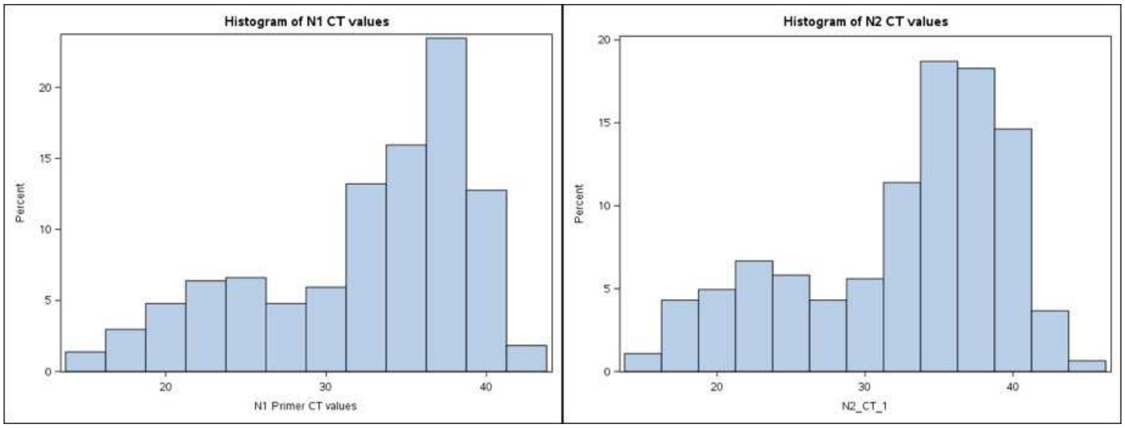

Supplementary Figure S2. Distribution of cycle threshold values for the N1 and N2 PCR reactions

The median Ct results were 32 and 33, respectively for the N1 and N2 targets. Results for detections between >40 and 45 are noted in text but are not included in any of our summary results otherwise.

Supplementary Figure S3

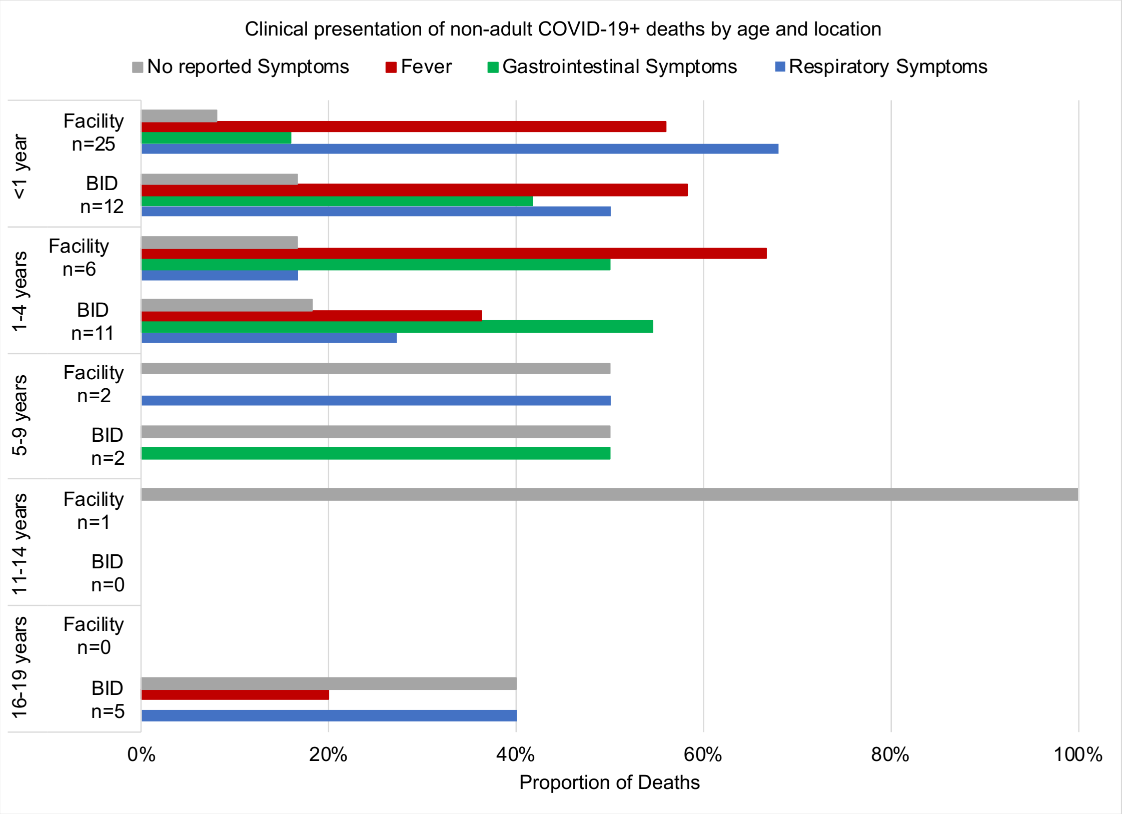

Supplementary Figure S3. Clinical presentations among COVID-19 positive children ≤19 years

For this analysis, we extracted the data about clinical presentation of each case from the medical charts (facility deaths) or verbal autopsy data (community deaths). We then clustered these syndromically into those that were ‘respiratory’ vs. ‘gastrointestinal’ vs. ‘other’. Since ‘fever’ could occur in any of these, or by itself, we summarized this separately. As can be seen, the syndromic presentation of COVID-19+ infants <1 is distinct from that in older children. In the infants, we see a high proportion with gastrointestinal complaints and a relative paucity with respiratory disease. In the older children, this pattern reverses, and in adolescents the pattern is consistent with that seen in adults, i.e., various combinations of respiratory symptoms, often accompanied by fever. Since this is an analysis by individual, there was no need to make weighting adjustments to account for enrollment ratios.
