# Supplementary material for: What is the prevalence of COVID-19 detection by PCR among deceased individuals in Lusaka, Zambia? A postmortem surveillance study
Source: BMJ Open. 2022 Dec 6;12(12):e066763. doi: 10.1136/bmjopen-2022-066763 (PMC9729848; doi:10.1136/bmjopen-2022-066763)
Supplement: Author's manuscript [file bmjopen-2022-066763.draft_revisions.pdf]

BMJ Open is committed to open peer review. As part of this commitment we make the peer review history of every article we publish publicly available.

When an article is published we post the peer reviewers' comments and the authors' responses online. We also post the versions of the paper that were used during peer review. These are the versions that the peer review comments apply to.

The versions of the paper that follow are the versions that were submitted during the peer review process. They are not the versions of record or the final published versions. They should not be cited or distributed as the published version of this manuscript.

BMJ Open is an open access journal and the full, final, typeset and author-corrected version of record of the manuscript is available on our site with no access controls, subscription charges or pay-per-view fees (http://bmjopen.bmj.com).

If you have any questions on BMJ Open's open peer review process please email info.bmjopen@bmj.com

BMJ Open

Sustained high prevalence of COVID-19 deaths from a systematic post-mortem study in Lusaka, Zambia: one year later

Journal:	*BMJ Open*
Manuscript ID	bmjopen-2022-066763
Article Type:	Original research
Date Submitted by the Author:	20-Jul-2022
Complete List of Authors:	Gill, Christopher; Boston University School of Public Health, Department of International Health<br>Mwananyanda, Lawrence; Boston University School of Public Health, Research<br>MacLeod, William; Boston University School of Public Health, Global Health<br>Kwenda, Geoffrey; University of Zambia - Ridgeway Campus, Biomedical Sciences<br>Pieciak, Rachel; Boston University School of Public Health, Global Health<br>Etter, Lauren; Boston University School of Public Health, Department of Global Health<br>Bridges, Daniel; Program For Applied Technology In Health (PATH)<br>Chikoti, Chilufya; Avencion Limited<br>Chirwa, Sarah; Avencion Limited<br>Chimoga, Charles; Avencion Limited<br>Forman, Leah; Boston University School of Public Health, Department of Global Health; Boston University School of Public Health<br>Katowa, Ben; Macha Research Trust<br>Lapidot, Rotem; Boston Medical Center, pediatric infectious diseases<br>Lungu, James; Avencion Limited<br>Matoba, Japhet; Macha Research Trust<br>Mwinga, Gift; Avencion Limited<br>Mubemba, Benjamin; The Copperbelt University, Wildlife Sciences<br>Mupila, Zachariah; Avencion<br>Muleya, Walter; University of Zambia School of Veterinary Medicine, Biomedical Sciences<br>Mwenda, Mulenga; Program for Applied Technology in Health<br>Ngoma, Benard; Avencion Limited<br>Nakazwe, Ruth; University of Zambia University Teaching Hospital, Biomedical sciences<br>Nzara, Diana; Avencion Limited<br>Pawlak, Natalie; Tufts University School of Medicine<br>Pemba, Lillian; Avencion Limited<br>Saasa, Ngonda; University of Zambia School of Veterinary Medicine<br>Simulundu, Edgar; Macha Research Trust<br>Yankonde, Baron; Avencion Limited<br>Thea, Donald; Boston University School of Public Health, Global Health
Keywords:	COVID-19, Epidemiology < INFECTIOUS DISEASES, PUBLIC HEALTH

SCHOLARONE™
Manuscripts

I, the Submitting Author has the right to grant and does grant on behalf of all authors of the Work (as defined in the below author licence), an exclusive licence and/or a non-exclusive licence for contributions from authors who are: i) UK Crown employees; ii) where BMJ has agreed a CC-BY licence shall apply, and/or iii) in accordance with the terms applicable for US Federal Government officers or employees acting as part of their official duties; on a worldwide, perpetual, irrevocable, royalty-free basis to BMJ Publishing Group Ltd ("BMJ") its licensees and where the relevant Journal is co-owned by BMJ to the co-owners of the Journal, to publish the Work in this journal and any other BMJ products and to exploit all rights, as set out in our licence.

The Submitting Author accepts and understands that any supply made under these terms is made by BMJ to the Submitting Author unless you are acting as an employee on behalf of your employer or a postgraduate student of an affiliated institution which is paying any applicable article publishing charge ("APC") for Open Access articles. Where the Submitting Author wishes to make the Work available on an Open Access basis (and intends to pay the relevant APC), the terms of reuse of such Open Access shall be governed by a Creative Commons licence – details of these licences and which Creative Commons licence will apply to this Work are set out in our licence referred to above.

Other than as permitted in any relevant BMJ Author's Self Archiving Policies, I confirm this Work has not been accepted for publication elsewhere, is not being considered for publication elsewhere and does not duplicate material already published. I confirm all authors consent to publication of this Work and authorise the granting of this licence.

For peer review only

TITLE: Sustained high prevalence of COVID-19 deaths from a systematic post-mortem study in Lusaka, Zambia: one year later

AUTHORS

Christopher J Gill [1], Lawrence Mwananyanda [1,2], William MacLeod [1], Geoffrey Kwenda [3], Rachel Pieciak [1], Lauren Etter [1], Daniel Bridges [4], Chilufya Chikoti [2], Sarah Chirwa [2], Charles Chimoga [2], Leah Forman [5], Ben Katowa [6], Rotem Lapidot [1], James Lungu [2], Japhet Matoba [6], Gift Mwinga [2], Benjamin Mubemba [7], Zachariah Mupila [2], Walter Muleya [8], Mulenga Mwenda [8], Benard Ngoma [2], Ruth Nakazwe [2, 9], Diana Nzara [2], Natalie Pawlak [10], Lillian Pemba [2], Ngonda Saasa [8], Edgar Simulundu [6], Baron Yankonde [2] and Donald M. Thea [1].

Corresponding Author: Christopher J. Gill, MD MS, Professor, Dept. of Global Health, Boston University School of Public Health, cgill@bu.edu

AFFILIATIONS

1. Boston University School of Public Health, Department of Global Health, Boston, Massachusetts, USA
2. Avencion Limited, Lusaka, Zambia
3. University of Zambia, School of Health Sciences, Department of Biomedical Sciences, Lusaka, Zambia
4. Program for Applied Technology in Health (PATH), Malaria Control and Elimination Partnership for Africa, Lusaka, Zambia
5. Biostatistics and Epidemiology Data Analytics Center, Boston University School of Public Health, Boston, Massachusetts, USA
6. Macha Research Trust, Choma, Zambia
7. Copperbelt University, School of Natural Resources, Department of Wildlife Sciences, Kitwe, Zambia
8. University of Zambia, School of Veterinary Medicine, Department of Biomedical Sciences, Lusaka, Zambia
9. University Teaching Hospital, Department of Microbiology, Lusaka, Zambia
10. Tufts University school of Medicine, Boston, Massachusetts, USA

For peer review only

What is already known on this topic

- Previously, we reported that COVID-19 was present among 15-19% of all decedents passing through a busy city morgue in Lusaka.
- Data documenting the mortal impact of COVID-19 in Africa remain sparse.
- Several modeling groups have also argued that COVID-19's impact in Africa has been underreported and hence underestimated.

What this study adds

- Antemortem testing for COVID-19 captured only ~10% of COVID-19 positive individuals indicating a substantial gap in surveillance.
- During peak transmission periods, ~90% of all deceased individuals tested positive for COVID-19.
- Most COVID-19 positive deceased adults presented with symptoms typical of COVID-19, arguing that COVID-19 caused their deaths and was not a co-incidental finding.
- Deaths occurred across the age spectrum, including among young children, indicating a different pattern of impact from what has been seen in high income country settings.
- We document three waves of transmission, attributable to the AE.1 lineage, and the Beta and Delta variants, respectively.

For peer review only

ABSTRACT

Objectives

Sparse data documenting the impact of COVID-19 in Africa fostered the belief that COVID-19 'skipped Africa'. We previously published systematic postmortem surveillance at a busy inner-city morgue in Lusaka, Zambia where COVID-19 was detected in 15-19% of all deaths. Because the decedents had rarely been tested in life, we concluded that cases had been undercounted. This conclusion rested on a relatively small cohort. Thus we conducted a longer larger follow-on survey to measure the prevalence of COVID-19 among deceased individuals in Lusaka.

Methods

Enrolling at the University Teaching Hospital Morgue, we obtained a nasopharyngeal swab from each decedent and tested these using quantitative polymerase chain reaction (RT-qPCR). We defined a positive if the PCR assay yielded cycle threshold values <40 for both the N1 and N2 SARS-2 gene. A subset of samples underwent genotyping. We weighted results to adjust for enrollment ratios and stratified by setting (facility vs. community), time of year, age, and location.

Results

From 1,118 decedents enrolled between Jan-June 2021, COVID-19 was detected among 32.0% (358/1,116). Roughly four COVID-19+ community deaths occurred for every facility death. Antemortem testing occurred for 52.6% (302/574) of facility deaths but only 1.8% (10/544) of community deaths and overall, only ~10% of COVID-19+ deaths were identified in life. During peak transmission periods, COVID-19 was detected in ~90% of all deaths. We observed three waves of transmission that peaked in July 2020, January 2021, and ~June 2021: the AE.1 lineage and the Beta and Delta variants, respectively.

Conclusions

COVID-19 was widely prevalent among deceased individuals in Lusaka and occurred in all age groups. Antemortem testing was performed for ~10% for the COVID-19 deaths, and almost never for community deaths, which comprised the majority by a four to one ratio. This indicates that the burden of COVID-19 has been substantially underestimated in Lusaka.

For peer review only - http://bmjopen.bmj.com/site/about/guidelines.xhtml

INTRODUCTION

Despite the evident harm from the COVID-19 pandemic globally, accurate data about the pandemic's mortal impact in Africa remain sparse. A recent multi-site data identified the severe impact of COVID-19 to deaths in African intensive care units, which argues for impact. However, the ICU population is not broadly generalizable, and such observations do not yield a direct estimate of the overall prevalence of COVID-19 or its impact at a population level.[1] In early 2021, our team reported results from systematic COVID-19 post-mortem surveillance conducted from June to October 2020 at the central morgue in Lusaka, Zambia.[2] Using polymerase chain reaction (PCR), we detected COVID-19 in nearly 20% of decedents, most of whom had died in the community. In that group, none had been tested for COVID-19 prior to death. Yet, even among facility deaths antemortem testing was uncommon. Hence, COVID-19 deaths only appeared rare in Lusaka because testing was rarely done. Consequently, we theorized that the so-called 'Africa Paradox'[3-8] was a myth born from insufficient surveillance data. Other groups have reached similar conclusions.[9 10]

Our analysis yielded other provocative findings. In contrast with the US and other high-income country (HIC) settings where deaths are concentrated in the elderly, COVID-19+ deaths in Lusaka occurred more evenly across the age spectrum. Further, 10% of COVID-19+ deaths occurred in children, a surprising finding given how rare pediatric COVID-19 deaths are in HICs. It remains an open question whether these pediatric deaths were caused by COVID-19 or whether the virus was a coincidental finding.

These conclusions were conservative given that our analysis rested on only seventy COVID-19+ decedents. Subsequently, our team conducted a second round of post-mortem COVID-19 surveillance, spanning the period January through June 2021. In this larger study we again quantified the burden of fatal COVID-19 by setting (community vs. facility deaths), calendar date, and geography; and observed the impact and patterns of clinical presentation among children vs. adults. In addition, we aligned our prevalence data with genetic sequencing to characterize shifts in viral lineages/variants over time.

METHODS

Overview

Our COVID-19 surveillance work builds upon a larger body of postmortem surveillance work initiated to define the fatal burden of Respiratory Syncytial Virus and *Bordetella pertussis*. With the pandemic, we amended our protocols to expand surveillance to all age ranges and to test for COVID-19. The RSV results have now been published,[11-13] and the pertussis results are currently under review.

We direct the readers to our prior publication for complete details of the surveillance methods.[2] Throughout this paper, we refer to several rounds of surveillance. Round 1 ran from June-Oct 2020; Round 2, comprising the new data in this paper, ran from Jan-June 2021. Round 3 is ongoing, was initiated early in 2022, and is planned to run through January 2023. These surveillance windows were the consequence of funding cycles and not based on assumptions about COVID-19 prevalence over time.

For peer review only - http://bmjopen.bmj.com/site/about/guidelines.xhtml

The research was conducted with the approval of the ethical review boards from Boston University (reference number H-36469) and the University of Zambia (reference number 015-11-18); written informed consent was obtained from the next of kin or family representative in all cases.

Sampling strategy

When capturing a high proportion of all deaths, postmortem surveillance is robust against ascertainment biases and provides a direct and unambiguous measure of a disease's prevalence, though inferring causality due to the disease may still be challenging. We enrolled deceased individuals at the University Teaching Hospital (UTH) morgue. Given concerns about viral RNA degradation leading to false negative results, enrollment was restricted to deaths within the preceding 48 hours.

UTH is the main teaching hospital for the University of Zambia Medical School. Its morgue is the largest in the city and contributes/accounts for 80% of deaths of burial certificates issued by the council office in the city, irrespective of socio economic status of the decedent. This, plus the legal requirement to obtain a burial certificate, nearly all of which are obtained from the UTH medical examiner's office, makes the UTH morgue highly representative of all deaths in Lusaka.

Due to the high volume in the morgue and finite capacity of our team, we capped enrollments at ~5-6 deaths per day. Also due to high volume, we enrolled community deaths at a 1:3 ratio, while enrolling the less common facility deaths at a 1:1 ratio. In response to the surprising high proportion of pediatric deaths in surveillance Round 1, we elected to also oversample this group enrolling infants (<1 year) at a 1:1 ratio. This required that we adjust for these ratios in our total population prevalence estimates (see statistical analysis). Our team had no access to the clinical data about each decedent and could not know the PCR results prior to consent and enrollment. Therefore, we were confident that our enrolment strategy was robust against selection biases with respect to COVID-19 status.

Concurrently, we extracted total deaths by age stratum from the official government burial registry. This allowed us to estimate the contemporaneous proportion of all deaths represented by our sample, and, by comparing the age by death distribution of the two groups, assess whether the enrolled sample was representative of the total.

Data collection and case definitions

We defined a 'facility death' as one that occurred under care at UTH or a referring facility. We defined a community death as one that occurred outside of medical care. We defined pediatric deaths as those occurring between 0-≤19 years, recognizing that some may choose to define the end of adolescence differently, and that such definitions may not align with official age of emancipation laws in different parts of the world.

Following enrollment, individual and household demographic data were collected on all decedents, along with any antemortem COVID-19 testing results that were documented in the medical chart (for facility deaths) or reported by the next of kin/representative of the decedent during the verbal autopsy (for community deaths).

For peer review only - http://bmjopen.bmj.com/site/about/guidelines.xhtml

A key challenge in this work is determining the causal role of COVID-19 in each death. Inferring causality is a challenge in post mortem studies: the SARS-CV19 virus may have a direct or indirect role in deaths, or could be coincidental. The US CDC has issued guidance stating that in deaths where the it is detected, the virus should be assumed to be the direct or underlying cause of death, absent exonerating circumstances.[14] That notwithstanding, to better infer the causal role of COVID-19 in deaths, we used the clinical data describing symptoms during the fatal event. For facility deaths, these data were obtained from a medical chart extraction focusing on symptoms at initial presentation to UTH; for community deaths, we conducted a verbal autopsy using the abbreviated tool validated by the Institute for Health Metrics Evaluation (IHME).[15] From these syndromic data, using the same approach as was used in our initial publication,[2] we classified COVID-19 PCR+ adult decedents (>19 years) into the following categories:

1) Probable COVID-19 deaths, where the individual presented with any combination of fever and/or respiratory symptoms;
2) Possible COVID-19 deaths, where the individual presented without classic respiratory symptoms, but rather with known sequalae of COVID-19 suggestive of a heart attack, stroke, or other acute vascular event related to COVID-19's propensity to increase the risk of clotting disorders;[16]
3) Probably not COVID-19 deaths, where the individual had an exonerating cause of death suggesting that the COVID-19 finding on PCR was incidental.
4) Unknown, where the data were insufficient to adjudicate.

The reasons we only applied this strategy to adults is that it quickly proved ill-suited for children. Unlike adults, where fever and/or respiratory symptoms predominated, the pediatric presentations were more variable and respiratory symptoms less common. Since we were (and remain) uncertain about how COVID-19 should present clinically in African children, we were concerned that creating *a priori* case definitions based on how the virus behaves in adults could be misleading. Similarly, applying a new definition developed iteratively from the constellation of symptoms observed in children and then applying that definition forwards without validation against an external gold standard (such as histopathology coupled with PCR), constitutes a logical circularity.

Facing this uncertainty, we opted simply to describe the pediatric case presentations without attempting to infer causality for those aged ≤19 years.

Biological sample collection

From each deceased individual, we obtained posterior nasopharyngeal (NP) samples. We used flocked-tipped NP swabs (Copan Diagnostics, Murietta, CA, USA),[17 18] inserting the swab into both nares and then rotating 180 degrees in both directions and collected into 3mL vials of universal transport media. Samples were stored cold or on ice until processing at our onsite molecular lab.

Laboratory procedures

PCR testing for COVID-19

After vortexing to elute samples, total nucleic acid for PCR was extracted using the EasyMAG system (Biomerieux, Marcy L'Etoile, France).[19] We ran PCR for 45 cycles

For peer review only - http://bmjopen.bmj.com/site/about/guidelines.xhtml

using the US CDC's COVID-19 testing kit, which targets the N1 and N2 nucleocapsid proteins.[20] We also ran PCR on each sample against the constitutive human enzyme RNAseP. The presence of RNAseP indicates that the NP swab made effective contact with the respiratory mucosa and that there were no PCR inhibitors.

We defined a COVID-19 test result as positive if the PCR cycle threshold (Ct) was <40 for both the N1 and N2 genes, if each assay demonstrated a logarithmic fluorescence amplification curve, had RNAseP detectable at Ct<40, and had positive and negative plate controls performing as anticipated.

However, Ct is a continuous variable, and the selection of any Ct cut point is arbitrary and hence controversial.[21] While a lower Ct, reflecting a higher viral load, is often associated with the degree of clinical severity, most COVID-19 surveillance studies assess individuals when they presented acutely at such a time when viral loads, and hence PCR signals, are likely at peak intensity. By contrast, the population in this study were all tested after they had died. At that point, the viral load and PCR signal intensity could have declined (or be undetectable), even if COVID-19 had set in motion a set of events culminating in death. For this reason we accepted a Ct <40 as positive, even though lower Ct values have often been used for defining COVID-19 status at the time of initial presentation. But since this substitutes one arbitrary cut point (<40) for another arbitrary cut point (<30), we also report the number of decedents with COVID-19 detectable between Ct >40 to ≤ 45, though we have not included these in our summary analyses.

Genetic sequencing of selected samples

To characterize the distributions of novel COVID-19 variants, we conducted genetic sequencing to detect viral variants on the subset of samples with a Ct <30. Prior experience has shown that nucleic acid concentrations are rarely sufficient for sequencing above this threshold. For this, we tested samples both from surveillance Rounds 1 and 2, describing shifts in dominant COVID-19 variants in Lusaka over the cumulative period of surveillance.

Total RNA was re-extracted from vortexed NP swab specimens using the QIAamp® Viral RNA Mini Kit (Qiagen, Hilden, Germany) as prescribed by the manufacturer. Complementary DNA was synthesized using LunaScript® RT SuperMix Kit (New England Biolabs, Ipswich, Massachusetts, USA) according to the manufacturer's protocol and multiplex PCR was conducted using custom primers, which were designed using Geneious software version 10.0.9, as used previously to sequence the first COVID-19 case in Zambia using the Sanger method.[22 23] The multiplex PCR generated overlapping amplicons which were then sequenced on the MinION (Oxford Nanopore Technology, United Kingdom) platform. The samples were multiplexed using the Oxford Nanopore native barcoding expansion kits 1–12 and 13–24 in combination with the ligation sequencing kit 109 (Oxford Nanopore). The data generated through the MinION was processed using the standard ARTIC bioinformatic pipeline (https://artic.network/ncov-2019/ncov2019-bioinformatics-sop.html). After removal of sequencing primers, the consensus sequences were deposited in the GISAID database (https://www.gisaid.org/). PANGO lineages were then determined for each genome using Pangolin v3.1.16 (https://pangolin.cog-uk.io/).

For peer review only - http://bmjopen.bmj.com/site/about/guidelines.xhtml

Statistical analysis

Given the uncertainties of future detection rates for COVID-19, our sampling was not based on a *priori* power assumptions. Rather, we sought to enroll as many decedents as our team could reasonably accommodate. Our statistical analysis was a straightforward comparison of proportions that did not require statistical modeling or imputation. COVID-19 status, based on PCR, was stratified by setting (community vs. facility deaths), by age groups, by calendar date, and by geography (city ward).

Where indicated, we have adjusted prevalence estimates by calculating weighted outputs to account for the different sampling ratios used during enrolment. We calculated weights so that the sum of the weighted population would sum to the sampled population: unweighted sample (1,118) = weighted sample (1,118). This allowed us to calculate the relative frequency of our unweighted sample in the four weighted groups: (1) facility deaths < 1 year, (2) all other facility deaths, (3) community deaths < 1 year of age, and (4) all other community deaths. We sampled the facility deaths < 1 year, all other facility deaths, and community deaths in infants < 1 year at a 1:1 ratio, and the community deaths ≥ 1 year at a 1:3 ratio. We then calculated the weighted sample by multiplying the inverse of the sampling ratio and calculated the relative frequency of the weighted sample. Setting the weighted sample size at the actual enrolled sample size of 1,118, yielded sample weights of 0.53 for the portion of the sample that we recruited as 1:1 and the sample weight of 1.60 for the portion of the sample that we recruited as 1:3. Hence, the ratio of these sample weights, adjusting for rounding, is 1.60/0.53 =~3:1. The reason for this weighting should be self-evident, since without such weighting the prevalence estimates will necessarily over or under estimate the true prevalence across different groups of decedents.

For analyses of COVID-19 deaths over time and place and for the genetic sequence data, we combined results from Rounds 1 and 2 of surveillance. Effectively this provides a time series analysis over a 1-year period minus the 3-month gap from October 2020-Jan 2021 defined by the end and start of adjacent funding cycles. Where appropriate, we reference our results from Round 1 to compare and contextualize results from Round 2. For all statistical analyses and most data manipulations, we used SAS software (Cary, North Carolina, USA).

For the geospatial analyses, we used ArcGIS software (ESRI, Redlands, California, USA). We pulled population size data from the Zambia Data Hub: https://zambia-open-data-nsdi-mlnr.hub.arcgis.com/, which is managed by the Government of Zambia through the Ministry of Lands and Natural Resources, and the Zambia Statistics Agency. We downloaded these ArcGIS layers to the level of Lusaka's city wards. We then created a 2-dimensional heat map that indexed the total number of CV19+ deaths against total enrolled deaths across each of Lusaka's main city wards.

Patient and Public Involvement

The participants in this study were deceased. There was no involvement of patients or the public in the design of this study. The results of this research have already been made publicly available via a posting on MedRXiv.

For peer review only - http://bmjopen.bmj.com/site/about/guidelines.xhtml

RESULTS

Study overview and summary of PCR results

This second round of surveillance spanned January-June 2021. The newly enrolled cohort included 1,118 deceased individuals ranging in age from <1 to 102 years. Contemporaneously, 6,270 deaths were reported in the Lusaka burial registries. Thus, our sample represented 17.8% of all deaths in this period. The age distribution of the enrolled vs the total deceased population were similar, arguing to representativeness (**Supplementary Figure S1**).

PCR was successfully conducted on 100% of the NP swabs, and 1,116 had an RNAseP <40, indicating adequacy of sample collection. COVID-19 was detected among 29.3% (327/1,116) of decedents without weighting, and 32.0% (effective sample size = 358/1,116) after adjusting for weighting (**Table 1**).

Table 1. Postmortem COVID-19 PCR results, stratified by setting

Setting of death	Negative, CT≥40	Positive, CT<40	Totals
**Unweighted**			
**Community % (n/N)**	66% (358/543)	34% (185/543)	49% (543/1,116)
**Facility % (n/N)**	75% (431/573)	25% (142/573)	51% (573/1,116)
**Both settings combined**	71% (789/1,116	29% (327/1,116)	100% (1,116/1,116)
**Weighted**			
**Community % (n/N)**	65% 528.2	35% 282.7	72.7% 811
**Facility % (n/N)**	75% 229.5	25% 75.6	27.3% 305
**Both settings combined**	68% (757.7/1,116)	32% (358.3/1,116)	100% (1,116/1,116)

Thus, the overall prevalence of COVID-19 in Round 2 was roughly double what we observed in Round 1 (16%, 58/364). The median Ct values for the N1 and N2 targets were ~33 in both cases (**Supplementary Figure S2)**. We note that an additional 58 samples (45.8 weighted) had COVID-19 detectable at a Ct ≥40-45 but were not included in subsequent analyses.

The weighted proportion of females among the COVID-19+ and COVID-19- groups was nearly identical (40.6%, 453/1,116). By contrast, the COVID-19+ decedents were significantly older than those without COVID-19 ((median 48 years (IQR 30-70 years) vs. 39 years (IQR 0-58 years), Wilcoxon Ranked Sum test *p*-value < 0.001 (**Table 2**)).

For peer review only - http://bmjopen.bmj.com/site/about/guidelines.xhtml

Table 2. Basic demographics of the postmortem cohort, stratified by COVID-19 status

Demographic characteristic	Negative, CT≥40 (n=789)	Positive, CT<40 (n=327)	All Deaths (n=1,116)
**Unweighted percent Females (n/N)**	42.3% (334/789)	41.0% (134/327)	41.9% (468/1,116)
**Weighted percent female (n/N)**	41.0% 309/758	39.9% 144/358	40.6% 453/1,116
**Unweighted median age at death, years (IQR)**	32.5 (0 - 54)	44.0 (27 - 68)	36.0 (1 - 58)
**Weighted median age at death (IQR)**	39.0 (10-58)	48 (30-70)	41 (23-64)

Proportion of deaths and COVID-19+ deaths by facility vs. community setting

Within the 1,116/1,118 (99.8%) of total deaths with a valid test result, there were 573 facility deaths and 543 community deaths. While this implies that the ratio of facility to community deaths was similar, this does not account for the different enrollment ratios applied to each group. After weighting, 73% of all deaths occurred in the community vs. 27% at a facility, for a ratio of ~three community deaths for each facility death.

Similarly, COVID-19 was detected among 24.8% (142/573) of facility deaths and 34.0% (185/543) of community deaths. Considering the contribution of deaths by setting, among the 327 decedents with COVID-19, 56.6% (185/327) were community deaths and 43.4% (142/327) were facility deaths. After weighting to adjust for the differing enrollment ratios, community deaths accounted for 79% and facility deaths for 21% of total COVID-19+ deaths. Stated another way, for each COVID-19+ death at a facility, ~four COVID-19+ deaths occurred in the community.

COVID-19 deaths by age group

During Round 1, the proportion of decedents with COVID-19 was similar across all age groups at, and 10% of all COVID-19+ deaths were in children ≤19 years.

Round 2 yielded a similar pattern with COVID-19+ deaths occurring across all age groups and with relatively similar proportions out of total deaths in each group (see **Figure 1**). After weighting, adults aged >19 years comprised 86.2% (300/358) of COVID-19+ deaths and children 0 to ≤19 years for 14.9% (53/358) (**Table 3**).

For peer review only - http://bmjopen.bmj.com/site/about/guidelines.xhtml

Table 3. Distribution of CV19 positive deaths by 20-year increments

Age at death (years)	Ct>=40 (Neg)	Ct<40 (Pos)	Total	Ct>=40 (Neg)	Ct<40 (Pos)	Total
	**Unweighted**			**Weighted**		
0-19	308<br>82.8%	64<br>17.2%	372	205.6<br>79.4%	53.3<br>20.6%	261.0
20-39	159<br>66.5%	80<br>33.5%	239	184.8<br>67.1%	90.5<br>32.9%	275.3
40-59	171<br>73.4%	62<br>26.6%	233	190.1<br>74.8%	63.9<br>25.2%	253.9
60-79	108<br>58.1%	78<br>41.9%	186	120.3<br>55.9%	94.8<br>44.1%	215.1
80-99	42<br>52.5%	38<br>47.5%	80	55.4<br>52.0%	51.1<br>48.0%	106.5
100+	0<br>0.0%	2<br>100.0%	2	0<br>0.0%	3.2<br>100.0%	3.2
Total	788*	324*	1,112*	757.7*	358.4*	1,112.1*

*4 had missing age data, of which 3 were CV19 positive and 1 was CV19 negative, for 789, 327 and 1,116, respectively

Row results are N (Top) and % (Bottom) in each cell.

Overall, 78.1% (208/358, weighted) of COVID-19+ decedents were aged <60 years. Contrasting the death by age distributions for the COVID-19+ decedents relative to the age distributions for the total population from the burial registries, the COVID-19+ deaths were relatively underrepresented among children and relatively over represented in the very elderly. Outside of the age extremes, the age by death distributions for the two cohorts were similar (**Supplemental Figure S1**).

Antemortem testing for COVID-19

During Round 1, antemortem testing for COVID-19 was rarely done, occurring in only 10% of facility deaths and none of the majority of deaths that were in the community.

In Round 2, testing rates had improved but only among facility deaths. Within this group, 52.6% (302/574) were tested antemortem, of which 25 were reportedly positive (see **Supplementary Table S1**). During this period, nearly all COVID-19 testing at UTH relied on rapid antigen tests. However, among the community deaths, we were still only able to document antemortem testing for 1.8% (10/544) of COVID-19+ decedents.

Temporal shifts in COVID-19 prevalence, dominant variants, and geography

Given these changes between Round 1 and Round 2, we further explored the relationship between total deaths, COVID-19 prevalence, viral lineages, and geographic distribution in time series analyses. These results, combining Round 1 and 2 data, are presented in **Figure 2**.

For peer review only - http://bmjopen.bmj.com/site/about/guidelines.xhtml
For peer review only

Several features are noteworthy. First, the data collected in Round 1 document what in hindsight was likely the first, and smallest, of three waves of COVID-19 to occur in Lusaka during our surveillance period (**Panel A**). Noting the gap in surveillance from October 2020 to January 2021, wave 1 occurred in June-October 2020; wave 2 in January-February 2021; and wave 3 in May-June 2021. Our surveillance ended in June 2021, precluding observations about when wave 3 peaked and receded.

Second, during waves 2 and 3, the proportion of decedents who tested positive for COVID-19 peaked at 91.7% prevalence in January and 83.8% in June of 2021. Both represented a statistically significant increase over the average prevalence during Rounds 1 and 2 (ANOVA, P<0.001).

Third, each wave presented a distinct pattern in terms of prevalent viral lineages (**Panel B**). Combining Rounds 1 and 2 and selecting those with a Ct<30, we successfully sequenced 96 isolates. From these, during wave 1 lineage AE.1 was dominant. During wave 2 in Jan-Feb, the B.1.351 variant (the Beta variant) was dominant. This was supplanted by the emergence of the B1.617 variant (Delta) as wave 3, which accounted for 100% of all sequenced isolates during this period.

Concurrently, we mapped the distribution of COVID-19+ cases indexed against total deaths over time across Lusaka's major city wards (**Panel C**). The areas shaded dark blue represent areas where COVID-19 deaths were disproportionately high relative to total deaths, while those in light pink, light blue, or white are areas where COVID-19 deaths were underrepresented relative to total deaths. Within this taxonomy, the heaviest burden of COVID-19 fell in Lusaka's poorest and most densely populated areas that constitute the peripheral ring of peri-urban compounds to the city's north, west, and south, and largely spared the more affluent neighborhoods that cluster in the center of the city.

Clinical presentation of COVID-19 positive children and adolescents

Recalling that infants <1 year were oversampled in our cohort requiring weighting to draw overall prevalence estimates, most deaths in children and adolescents ≤19 y were in infants. With that caveat, **Supplementary Figure S3** summarizes the syndromic presentations for the COVID-19 positive deceased children <19 years, stratifying these by age groups and community/facility settings. Since we are describing these on a per individual basis, there was no need to weight these results.

The syndromic categorizations are not mutually exclusive. Clinical presentations appeared to vary by age and setting. Among infants who died at a facility, respiratory symptoms and fever predominated, whereas fever, respiratory and gastrointestinal symptoms occurred in similar proportions among those who died in the community. Among children 1-4 years, fever and gastrointestinal symptoms were more common than respiratory symptoms. Very few COVID-19+ deaths occurred in children aged 5-14 years (N=5). Among children 16-19 years (N=5, all from the community), the only reported symptoms were fever and respiratory distress, consistent with COVID-19's presentation in adults. In both community and facility settings, there were several children with no reported symptoms, though this may simply reflect a lack of documentation. Acknowledging the sparse data from older children, these results are broadly consistent with our observations in Round 1 where we had noted the relative

For peer review only - http://bmjopen.bmj.com/site/about/guidelines.xhtml

infrequency of respiratory symptoms and commonality of gastrointestinal symptoms, particularly among children ≤ 5 years. **Supplementary Table S2** provides a detailed line listing summarizing the clinical presentation of each CV19+ pediatric death by setting (facility deaths **Table S2a**, community deaths **Table S2b**).

Inferring the causal role of COVID-19 in these deaths

During Round 1, among those who had sufficient clinical data to allow an assessment of causality, nearly all were judged to have probably or possibly died from COVID-19.

In Round 2, 89.3% (292/327) of deaths had sufficient data for clinical adjudication, leaving 10.7% (45/327) that could not be adjudicated. Excluding the pediatric cases and those with insufficient data for analysis, we were able to adjudicate 219 deceased adults. Of these, 155/219 (70.8%) were considered ‘probably’ or ‘possibly’ due to COVID-19, and 29.2% ‘probably not’ due to COVID-19. When adjusted for weighting, this proportion rose to 73.9% (181/244) (see **Table 4**).

We recognized that the categories used for adjudication (probable, possible, probably not) rested on clinical information that may have been incomplete, a particular problem with the verbal autopsy data given that the respondent providing the information was necessarily relaying these data second hand. A gold standard for asserting causality would have been PCR coupled with histology showing end organ damage consistent with SARS-2 infections. Indeed, we had previously published such pathology based on minimally invasive tissue sampling (MITS) among several of the cases here that were diagnosed antemortem. As reported in Mudenda et al, 2021, these individuals showed evidence of diffuse organ injury to the lungs, kidneys and liver in patterns consistent with COVID-19 disease.[24] However, the identification of these decedents in life was unlikely done at random but rather based on clinical suspicion, creating the potential for ascertainment bias.

Given these limitations, we conducted causal inference analyses around the cycle threshold data for the full cohort, which we felt were robust against selection bias. Here, we reasoned that higher viral loads (and hence lower Ct values) would generally align with increased severity of disease. If so, we hypothesized that decedents deemed ‘probable COVID-19’, reflecting a high suspicion that COVID-19 was the cause of death, would on average have lower Ct values (reflecting higher viral loads) than those whose death was deemed ‘probably not’ COVID-19. The data in **Table 4** supported that hypothesis.

For peer review only - http://bmjopen.bmj.com/site/about/guidelines.xhtml

Table 4. Causal role of COVID-19 among PCR(+) deceased adults*, >19 years

Role of COVID-19 in death of patient	Median Ct (IQR)	Cases (N)	Percent	Weighted cases (N)	Weighted percent	Cumulative total for cases that could be adjudicated	Cumulative percent (n/N) for cases that could be adjudicated
**Probably due to COVID-19**	31.6 (24.2-35.5)	127	48.7%	142.2	46.6%	142	58.2% (142/244)
**Possibly due to COVID-19**	30.2 (23.2-35.7)	28	10.7%	38.3	12.6%	180	73.8% (180/244)
**Probably not due to COVID-19**	34.9 (28.0-37.3)	64	24.5%	63.9	20.9%	244	100% (244/244)
**Insufficient data to adjudicate causality**	32.5 (23.9-35.5)	42	16.1%	60.7	19.9%	Not applicable	Not applicable

* Clinical presentations of children with COVID-19 (n= 63) were variable and often lacked typical symptoms of respiratory distress. Since we were uncertain how COVID-19 should behave in children, we did not attempt to adjudicate these cases in terms of presumed causality. The results above have been weighted to adjust for the different enrollment ratios

For peer review only

For peer review only - http://bmjopen.bmj.com/site/about/guidelines.xhtml

6

For peer review only

Among those whose deaths were adjudicated as ‘probably’ due to COVID-19, the median Ct values were 31.6 (IQR CI 24.2-35.5) whereas those adjudicated as ‘probably not’ COVID-19 had a median Ct value of 34.9 (IQR 28-37.3), or a ten-fold increase in viral load. Thus individuals whose clinical data were most suggestive of a COVID-19 death had the highest viral loads.

Following this reasoning further, we conducted a similar analysis looking at the distribution of Ct values by ascending age groups. As noted previously, we were skeptical that COVID-19 was the cause of death in young children, largely because the clinical presentations in children, and especially children <5 years, differed from adults. Therefore we reasoned that COVID-19 was more likely a coincidental finding in children. To test that contention, we stratified the proportion of PCR results by age group sorting them into two groups: those with the strongest signals (i.e., Ct<30) and those with weaker signals (i.e., Ct 30≤Ct≤40). We then calculated the ratio of the proportions of weaker to strongest Ct values within each 20-year age stratum. We hypothesized that if COVID-19 was a co-incidental finding, we would observe a higher ratio of weaker to strong results than if COVID-19 was the cause of death, in which case we would see a much lower ratio. The results shown in **Supplementary Table S3** supported that hypothesis. After adjusting for weighting, the ratio of weaker to strongest Ct results was 6.6:1 for those aged 0-19 years, compared with a ratio of 1.9:1 across all ages combined. With each increase in age stratum, the proportion of weak to strong PCR signals declined and did so in a stepwise fashion, such that for those 80 years and older, it had fallen to a ratio of 1:1. We drew two conclusions from this analysis. First, the results supported our assumption that COVID-19 was likely a coincidental finding among children. Second, by extension, COVID-19 likely played a causal role in the deaths of older individuals.

DISCUSSION

This second round of postmortem surveillance for COVID-19 reinforces and expands upon our previous observations. First, even if we base our conclusions solely on those who were deemed ‘probably due to COVID-19’ based on clinical presentation, our data suggests that COVID-19 had a severe impact in Lusaka with much loss of life. Second, COVID-19 deaths were concentrated in the community where testing for COVID-19 was absent. Third, rather than being concentrated in the elderly, COVID-19+ deaths were distributed widely across the age spectrum, and most (~80%) were in individuals aged <60 years. Fourth, COVID-19 was frequently identified among children. Among children ≤5 years, gastrointestinal complaints were common and respiratory symptoms comparatively uncommon, and we suspect that COVID-19 was more likely a co-incidental finding in these younger individuals. Lastly, the emergence of the Beta and Delta variants coincided with marked increases in the proportion of COVID-19+ deaths, reaching ~90% peak prevalence at the peak of those waves. Conservatively, COVID-19 was identified in 30% of all decedents in our cohort from January to June 2021.

A consistent finding was the low proportion of decedents with COVID-19 who were diagnosed in life. We hypothesize that this can be explained as follows: 1) the majority of COVID-19+ deaths (~80%) occurred in the community where, with few exceptions, testing was unavailable; 2) among the hospital deaths, only half were tested; and 3) most of the testing done at facilities relied on rapid antigen tests, which are far less

For peer review only - http://bmjopen.bmj.com/site/about/guidelines.xhtml

sensitive than PCR.[25] Putting these in sequence, from the 1,118 decedents tested in our study we identified COVID-19 in 327 (unweighted). Of these, only 25 had been identified antemortem at UTH, or <10% of the total. Since these hospital data feed into the national COVID-19 surveillance data, we conclude that the impact of COVID-19 has been systematically and significantly under-represented. While it is certainly possible that individuals in the community might have self-tested for COVID using a rapid test, such results would unlikely be reported officially to the government of Zambia, and hence fail to be captured in official government reports. In that sense the extent of underreporting we identify could itself be an underestimate.

As in our prior analysis, COVID-19+ deaths were concentrated in Lusaka's most densely populated wards where Lusaka's poorest and most vulnerable citizens reside. Access to care is challenging in these communities, and we have described how delays in seeking care may contribute to infant deaths in the community.[26] This highlights a sad element to the global COVID-19 narrative, which is that COVID-19's impact is not shared equally. Even within a poor country like Zambia, there is a gradient of impact that falls hardest on those with the least resources to protect themselves.

There are several important distinctions from our findings in Round 1. First, the numbers of COVID-19 deaths and the proportion of deceased individuals who tested positive for COVID-19 both increased substantially.

Second, the proportion of these deaths that were probably or possibly judged as 'probably' or 'possibly' due to COVID-19 declined from ~100% in Round 1 to ~70 % in Round 2. We acknowledge that the assessment of causality was limited by the evidence present in the medical chart (facility deaths) or reported through the verbal autopsy (community deaths). With that caveat, our procedures and tools and field team for collecting these data were the same in rounds 1 and 2, leading us to conclude that this was a real shift and not an artifact of shifting study procedures.

Our data do not provide an explanation for this decline, but several explanations are plausible. One is that earlier population exposure to COVID-19 (following the first wave) might have provided increased resistance to the SARS-CoV2 virus, i.e., the change is explained by natural immunity from prior infection. COVID-19 vaccines were virtually unavailable during this period, so vaccine derived immunity cannot explain this. A second possibility is that the Beta and Delta variants dominated the second and third waves of transmission. Both variants exhibited increased transmissibility, which plausibly accounts for the high proportions of deaths with COVID-19 at the peak of each wave. As seen with the Omicron variant, a gain in transmissibility could yield a reciprocal loss of virulence, i.e., the change is explained by virology.[27 28] However, recent epidemiologic and *in vitro* data suggest the opposite, that the Beta and particularly Delta variants were more, not less, pathogenic.[29-31] Thus, we believe that the former explanation is more plausible.

Strengths and limitations

A strength was that our data were collected prospectively, capturing a wide spectrum of ages in community and facility settings and without prior knowledge of clinical presentation or antemortem testing results. While our capacity only allowed us to enroll a subset of all deaths at the morgue, enrollment was conducted without prior knowledge

For peer review only - http://bmjopen.bmj.com/site/about/guidelines.xhtml

of clinical presentation or PCR status, and thus should not introduce bias affecting overall prevalence estimates after adjusting for weighting. And while all came from the UTH morgue, 80% of all deaths in the city transit this facility, making it highly representative. Additionally, we were able to combine multiple data elements on the same individual: clinical presentation, molecular testing, geography, and viral lineage, to provide an overall picture of the impact of the COVID-19 pandemic in Lusaka during these periods.

Limitations include that there was a three-month gap in surveillance between Rounds 1 and 2, and that Round 2 ended prior to the resolution of Wave 3 caused by the Delta variant. These periods were defined by funding for the research, not by knowledge of COVID-19 prevalence. Our adjudications were necessarily limited by the completeness of clinical data for the facility deaths and by the accuracy of recall from non-medical persons through the verbal autopsy for community deaths. That could lead to some misclassification in our adjudications. Our data describe impact but cannot directly provide a case fatality rate since we lack concurrent incidence data for the general population. Lastly, while Round 2 data confirm identify a high proportion of pediatric deaths with COVID-19 and again show that gastrointestinal symptoms are common in children <5 years, we are cautious about inferring causality. In our judgement, resolving this question requires a higher gold standard, such as PCR coupled with histopathology. Our data do not address the impact of COVID-19 in terms of a case or infection fatality rate, but this question is being examined in an ongoing excess mortality analysis to be published separately.

The most challenging aspect of this work was inferring the causal role of COVID-19 in each death. We note that the clinical definitions applied during adjudication are themselves of limited precision. However, these are the same definitions we had used in our original report in The BMJ 2021, and we believe our approach here was defensible for several reasons. First, it is supported by guidance from the US CDC: given a patient who presented with respiratory distress and fever and died with a positive PCR test for COVID-19, it is no great leap of faith to infer that the death was due to COVID-19. Second, we conducted minimally invasive tissue sampling on a subset of decedents who had been identified with COVID-19 in life. As previously published, the pathology from these individuals was consistent with known patterns of COVID-19 disease, with evidence of lung and other organ dysfunction.[24] Additionally, we have shown that viral loads were higher among individuals whose deaths were deemed ‘probably’ due to COVID-19 than those deemed ‘probably not’ due to COVID-19. And we demonstrated that the ratio of weak to strong PCR signals was age dependent, with much higher ratios in those children and adolescents than older age groups. Combined, these results argue that COVID-19 was a causal factor for deaths in older individuals and more likely a co-incidental finding in those ≤19 years. Our confidence is lower for those deemed ‘possibly’ due to COVID-19. But such cases comprised a small proportion of all COVID-19 positive deaths in this analysis.

Conclusions

COVID-19 was highly prevalent in the post-mortem setting in Lusaka during the first half of 2021 reaching ~90% of deceased individuals during peak periods of transmission and 30% over the six months of observation. Despite increased rates of antemortem testing

For peer review only - http://bmjopen.bmj.com/site/about/guidelines.xhtml

for COVID-19, there was little commensurate increase in antemortem testing for those who died in the community, who constituted a 4:1 majority of COVID-19 positive deaths. Overall, only about 10% of those who died with COVID-19 were known have been infected in life yielding a ten-fold undercount. Considering recent reports demonstrating examples of underreporting of COVID-19 elsewhere, including inferential methods from excess mortality analyses,[10 32-37] we believe that our results are more likely typical than exceptional. If so, COVID-19's impact in Africa has been underestimated.

ACKNOWLEDGEMENTS

We wish to thank our team at the Bill & Melinda Gates Foundation for their support and encouragement: Prachi Vora, Padmini Srikanthia, and Keith Klugman.

For peer review only

For peer review only - http://bmjopen.bmj.com/site/about/guidelines.xhtml

FIGURE LEGENDS

Figure 1. Enrolled decedents and COVID-19+ deaths by 5-year age strata

Each stacked bar in the histogram shows the total number of enrolled decedents within each age stratum. The portion in yellow represents those who tested positive by PCR (Ct <40) for COVID-19 and those in grey those who tested negative (Ct ≥40). The % of positives is indicated at the top of each column. The totals have been adjusted to account for differing enrolment ratios.

Figure 2. Proportion of COVID-19+ deaths and viral lineages/variants, by time and geography

Panel A. The histograms represent the total number of COVID-19 positive deaths identified during the two rounds of surveillance. The gap from October 2020 to January 2021 represents the end and start of funding cycles. It is apparent that there were at least three waves of transmission in 2020-2021. Since our enrollment strategy shifted between Round 1 and Round 2, we have adjusted the proportions for the Round 2 data by weighting to account for the different enrolment ratios applied by age and setting.

Panel B. Combing the samples corresponding to each of the three waves, and the period in between waves 2 and 3, we clustered the viral lineages as shown on the top half of the figure. As can be seen, wave 1 was dominated by lineage AE.1, wave 2 by the ‘Beta’ variant, and wave 3 by the ‘Delta’ variant. For these data, we present the results without adjustments for weighting.

Panel C. Over these same time intervals, we summarize the distribution of cases indexed against total population within the major city wards of Lusaka. The colors correspond to a heat map defined by the union of ‘total deaths’ in pink and ‘COVID-19+’ deaths in blue, and therefore provide an indexed metric of COVID-19 deaths against all deaths. Those with darker shading indicate that COVID-19 deaths were disproportionately high relative to all deaths; those that are lightly shaded or white are where COVID-19 deaths were disproportionately low, relative to total deaths. As can be seen, the highest concentration of cases clustered in the peripheral areas of the city, which correspond to the poorest and most densely populated parts of the city. Whereas the wealthier sections, clustering in the middle of Lusaka, were relatively spared. Round 2 results have also been adjusted for enrollment ratios as in Panel A.

For peer review only

For peer review only - http://bmjopen.bmj.com/site/about/guidelines.xhtml

DECLARATIONS

Dissemination of results

Round 2 data were presented to members of the Zambian Ministry of Health and the Zambian Medical Association in July 2021, and the final manuscript was shared with the Zambian National Health Research Authority (NHRA) prior to submission. The results have been presented to our team at the Bill & Melinda Gates Foundation and to representatives from the US CDC and the Swiss Tropical Medicine Institute. These data have been shared with a modeling team at the Imperial College London to support our ongoing excess mortality analyses. Data from Round 1 were published previously in the BMJ.

Funding

The original ZPRIME study and this COVID-19 expansion were made possible through the generous support of the Bill & Melinda Gates Foundation (OPP 1163027). The funders had no role in designing the study; in the collection and analysis of data; or in the decision to submit the article for publication.

Data sharing

All data from this analysis can be shared based on a reasonable request that includes a statement of the proposed study's objectives and an analysis plan through a formal data sharing agreement between both parties. Similarly, we are willing to share biological samples based on any reasonable request and proposal and pending the completion of an appropriate materials transfer agreement authorized by the Government of Zambia.

Community engagement

Due to the time constraints around the pandemic, we did not engage the community on this project.

Data provenance

The lead authors (CJG and LM) affirm that the manuscript is an honest, accurate, and transparent account of the study being reported; that no important aspects of the study have been omitted; and that any discrepancies from the study as originally planned (and, if relevant, registered) have been explained.

Manuscript contributions

CJG was the principle investigator, conceived the project, secured the funding, assisted with the protocol and led on writing the manuscript; **LM** was the Zambian PI, assisted with protocol and tool development, and oversaw the field team and study implementation; **WBM** was the study statistician, developed the analysis plan and conducted the analysis; **GK** was the technical lead for the PCR lab in Lusaka; **RP** was

For peer review only

the project manager and a research fellow, developed the study proposal, oversaw study management, and contributed to key aspects of data analysis and presentation; **LE** was a research fellow and contributed to key aspects of data analysis and presentation; **RN, Chilufya Chikoti (CC#1**) and **BY** were the PCR technicians for our lab; **SC, LP, GM, DN** were the field data collection team under team leaders **BN** and **Charles Chimoga (CC#2, unrelated to CC#1**), who conducted grief counseling for the families, obtained consent, and collected all of the clinical data and the biological samples: **LF** supervised creation of the RedCAP data collection tools and oversaw data management, cleaning and retention; **RL** contributed to data interpretation and analysis; **JL** provided information technology support to the field team; **BK, JM, BM,** and **WM** conducted the genetic sequencing analyses under the supervision of **DB** and **ES**; **ZM** was the PCR lab manager; **NP** was a research fellow who supported some of the analyses; and **DMT** was our senior co-investigator who contributed to data analysis and interpretation.

Ethical review:

The research was conducted with the approval of the ethical review boards from Boston University (reference number H-36469) and the University of Zambia (reference number 015-11-18); written informed consent was obtained from the next of kin or family representative in all cases.

Conflict of Interest:

All authors declare that they have no conflicts of interest relevant to this publication.

For peer review only

REFERENCES

1. African C-CCOSI. Patient care and clinical outcomes for patients with COVID-19 infection admitted to African high-care or intensive care units (ACCCOS): a multicentre, prospective, observational cohort study. *Lancet* 2021;397(10288):1885-94. doi: 10.1016/S0140-6736(21)00441-4 [published Online First: 2021/05/24]
2. Mwananyanda L, Gill CJ, MacLeod W, et al. Covid-19 deaths in Africa: prospective systematic postmortem surveillance study. *Bmj* 2021;372:n334. doi: 10.1136/bmj.n334 [published Online First: 2021/02/19]
3. Zerfu TA, Tareke AA. What could be the potential reasons for relatively low coronavirus disease 2019 (COVID-19) fatality rates in Africa? The case for Ethiopia. *J Glob Health* 2021;11:03057. doi: 10.7189/jogh.11.03057 [published Online First: 2021/04/22]
4. Musa HH, Musa TH, Musa IH, et al. Addressing Africa's pandemic puzzle: Perspectives on COVID-19 transmission and mortality in sub-Saharan Africa. *Int J Infect Dis* 2021;102:483-88. doi: 10.1016/j.ijid.2020.09.1456 [published Online First: 2020/10/04]
5. Lawal Y. Africa's low COVID-19 mortality rate: A paradox? *Int J Infect Dis* 2021;102:118-22. doi: 10.1016/j.ijid.2020.10.038 [published Online First: 2020/10/20]
6. Bamgboye EL, Omiye JA, Afolaranmi OJ, et al. COVID-19 Pandemic: Is Africa Different? *J Natl Med Assoc* 2021;113(3):324-35. doi: 10.1016/j.jnma.2020.10.001 [published Online First: 2020/11/07]
7. Adams J, MacKenzie MJ, Amegah AK, et al. The Conundrum of Low COVID-19 Mortality Burden in sub-Saharan Africa: Myth or Reality? *Glob Health Sci Pract* 2021;9(3):433-43. doi: 10.9745/GHSP-D-21-00172 [published Online First: 2021/10/02]
8. Ntoumi F, Velavan TP. COVID-19 in Africa: between hope and reality. *Lancet Infect Dis* 2020 doi: 10.1016/S1473-3099(20)30465-5 [published Online First: 2020/06/20]
9. Warsame A, Bashiir F, Freemantle T, et al. Excess mortality during the COVID-19 pandemic: a geospatial and statistical analysis in Mogadishu, Somalia. *Int J Infect Dis* 2021;113:190-99. doi: 10.1016/j.ijid.2021.09.049 [published Online First: 2021/09/28]
10. Watson OJ, Alhaffar M, Mehchy Z, et al. Leveraging community mortality indicators to infer COVID-19 mortality and transmission dynamics in Damascus, Syria. *Nat Commun* 2021;12(1):2394. doi: 10.1038/s41467-021-22474-9 [published Online First: 2021/04/24]
11. Gill CJ, Mwananyanda L, MacLeod WB, et al. Infant deaths from respiratory syncytial virus in Lusaka, Zambia from the ZPRIME study: a 3-year, systematic, post-mortem surveillance project. *Lancet Glob Health* 2022;10(2):e269-e77. doi: 10.1016/S2214-109X(21)00518-0 [published Online First: 2022/01/23]
12. Murphy C, MacLeod WB, Forman LS, et al. Risk Factors for Respiratory Syncytial Virus-Associated Community Deaths in Zambian Infants. *Clin Infect Dis*

For peer review only

2021;73(Suppl_3):S187-S92. doi: 10.1093/cid/ciab453 [published Online First: 2021/09/03]

13. Forman LS, Macleod W, Mwananyanda L, et al. Association of Respiratory Syncytial Virus Infection and Underlying Risk Factors for Death Among Young Infants Who Died at University Teaching Hospital, Lusaka Zambia. *Clin Infect Dis* 2021;73(Suppl_3):S180-S86. doi: 10.1093/cid/ciab466 [published Online First: 2021/09/03]
14. Centers for Disease Control and Prevention. Guidance for certifying deaths due to coronavirus disease 2919 (COVID-19). Vital Statistics Reporting Guidance. Hyattsville, Maryland: National Center for Health Statistics, 2020.
15. Serina P, Riley I, Stewart A, et al. A shortened verbal autopsy instrument for use in routine mortality surveillance systems. *BMC Med* 2015;13:302. doi: 10.1186/s12916-015-0528-8 [published Online First: 2015/12/17]
16. Ramlall V, Thangaraj PM, Meydan C, et al. Immune complement and coagulation dysfunction in adverse outcomes of SARS-CoV-2 infection. *Nat Med* 2020;26(10):1609-15. doi: 10.1038/s41591-020-1021-2 [published Online First: 2020/08/05]
17. Van Horn KG, Audette CD, Sebeck D, Tucker KA. Comparison of the Copan ESwab system with two Amies agar swab transport systems for maintenance of microorganism viability. *J Clin Microbiol* 2008;46(5):1655-8. doi: 10.1128/JCM.02047-07 [published Online First: 2008/03/21]
18. Van Horn KG, Audette CD, Tucker KA, Sebeck D. Comparison of 3 swab transport systems for direct release and recovery of aerobic and anaerobic bacteria. *Diagn Microbiol Infect Dis* 2008;62(4):471-3. doi: 10.1016/j.diagmicrobio.2008.08.004 [published Online First: 2008/09/26]
19. Yang G, Erdman DE, Kodani M, et al. Comparison of commercial systems for extraction of nucleic acids from DNA/RNA respiratory pathogens. *J Virol Methods* 2011;171(1):195-9. doi: 10.1016/j.jviromet.2010.10.024 [published Online First: 2010/11/03]
20. Lu X, Wang L, Sakthivel SK, et al. US CDC Real-Time Reverse Transcription PCR Panel for Detection of Severe Acute Respiratory Syndrome Coronavirus 2. *Emerg Infect Dis* 2020;26(8) doi: 10.3201/eid2608.201246 [published Online First: 2020/05/13]
21. Gill CJ, Gunning CE, MacLeod WB, et al. Asymptomatic Bordetella pertussis infections in a longitudinal cohort of young African infants and their mothers. *Elife* 2021;10 doi: 10.7554/eLife.65663 [published Online First: 2021/06/08]
22. Mwenda M, Saasa N, Sinyange N, et al. Detection of B.1.351 SARS-CoV-2 Variant Strain - Zambia, December 2020. *MMWR Morb Mortal Wkly Rep* 2021;70(8):280-82. doi: 10.15585/mmwr.mm7008e2 [published Online First: 2021/02/26]
23. Simulundu E, Mupeta F, Chanda-Kapata P, et al. First COVID-19 case in Zambia - Comparative phylogenomic analyses of SARS-CoV-2 detected in African countries. *Int J Infect Dis* 2021;102:455-59. doi: 10.1016/j.ijid.2020.09.1480 [published Online First: 2020/10/10]
24. Mudenda V, Mumba C, Pieciak RC, et al. Histopathological Evaluation of Deceased Persons in Lusaka, Zambia With or Without Coronavirus Disease 2019 (COVID-19) Infection: Results Obtained From Minimally Invasive Tissue Sampling. *Clin*

For peer review only

For peer review only - http://bmjopen.bmj.com/site/about/guidelines.xhtml

Infect Dis 2021;73(Suppl_5):S465-S71. doi: 10.1093/cid/ciab858 [published Online First: 2021/12/16]
25. Scheiblauer H, Filomena A, Nitsche A, et al. Comparative sensitivity evaluation for 122 CE-marked rapid diagnostic tests for SARS-CoV-2 antigen, Germany, September 2020 to April 2021. *Euro Surveill* 2021;26(44) doi: 10.2807/1560-7917.ES.2021.26.44.2100441 [published Online First: 2021/11/06]
26. Lapidot R, Larson Williams A, MacLeod WB, et al. Verbal Autopsies for Out-of-Hospital Infant Deaths in Zambia. *Pediatrics* 2021;147(4) doi: 10.1542/peds.2020-1767 [published Online First: 2021/03/06]
27. Johnson AG, Amin AB, Ali AR, et al. COVID-19 Incidence and Death Rates Among Unvaccinated and Fully Vaccinated Adults with and Without Booster Doses During Periods of Delta and Omicron Variant Emergence - 25 U.S. Jurisdictions, April 4-December 25, 2021. *MMWR Morb Mortal Wkly Rep* 2022;71(4):132-38. doi: 10.15585/mmwr.mm7104e2 [published Online First: 2022/01/28]
28. Shuai H, Chan JF, Hu B, et al. Attenuated replication and pathogenicity of SARS-CoV-2 B.1.1.529 Omicron. *Nature* 2022 doi: 10.1038/s41586-022-04442-5 [published Online First: 2022/01/22]
29. Fisman DN, Tuite AR. Evaluation of the relative virulence of novel SARS-CoV-2 variants: a retrospective cohort study in Ontario, Canada. *Cmaj* 2021;193(42):E1619-E25. doi: 10.1503/cmaj.211248 [published Online First: 2021/10/07]
30. Zhang J, Xiao T, Cai Y, et al. Membrane fusion and immune evasion by the spike protein of SARS-CoV-2 Delta variant. *Science* 2021;374(6573):1353-60. doi: 10.1126/science.abl9463 [published Online First: 2021/10/27]
31. Tao K, Tzou PL, Nouhin J, et al. The biological and clinical significance of emerging SARS-CoV-2 variants. *Nat Rev Genet* 2021;22(12):757-73. doi: 10.1038/s41576-021-00408-x [published Online First: 2021/09/19]
32. Walker PGT, Whittaker C, Watson OJ, et al. The impact of COVID-19 and strategies for mitigation and suppression in low- and middle-income countries. *Science* 2020;369(6502):413-22. doi: 10.1126/science.abc0035 [published Online First: 2020/06/14]
33. Russell TW, Golding N, Hellewell J, et al. Reconstructing the early global dynamics of under-ascertained COVID-19 cases and infections. *BMC Med* 2020;18(1):332. doi: 10.1186/s12916-020-01790-9 [published Online First: 2020/10/23]
34. Koum Besson ES, Norris A, Bin Ghouth AS, et al. Excess mortality during the COVID-19 pandemic: a geospatial and statistical analysis in Aden governorate, Yemen. *BMJ Glob Health* 2021;6(3) doi: 10.1136/bmjgh-2020-004564 [published Online First: 2021/03/25]
35. Karlinsky A, Kobak D. Tracking excess mortality across countries during the COVID-19 pandemic with the World Mortality Dataset. *Elife* 2021;10 doi: 10.7554/eLife.69336 [published Online First: 2021/07/01]
36. Alves THE, de Souza TA, Silva SA, Ramos NA, de Oliveira SV. Underreporting of Death by COVID-19 in Brazil's Second Most Populous State. *Front Public Health* 2020;8:578645. doi: 10.3389/fpubh.2020.578645 [published Online First: 2021/01/02]

For peer review only

383 37. Van Gordon MM, Mwananyanda L, Gill CJ, McCarthy KA. Regional comparisons of
384 COVID reporting rates, burden, and mortality age-structure using auxiliary data
385 sources. *MedRxiv (preprint)* 2021 doi: 2021.08.18.212622482021

386

For peer review only

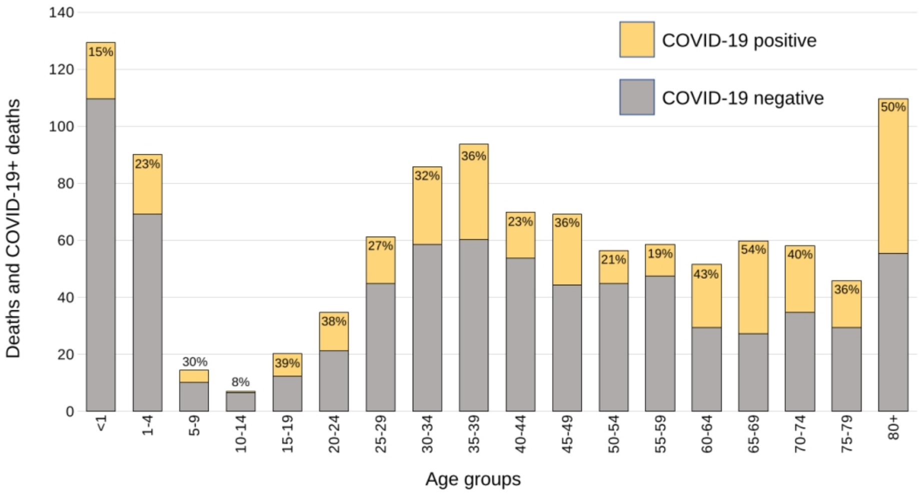

Figure 1. Enrolled decedents and COVID-19+ deaths by 5-year age strata

37058x20828mm (1 x 1 DPI)

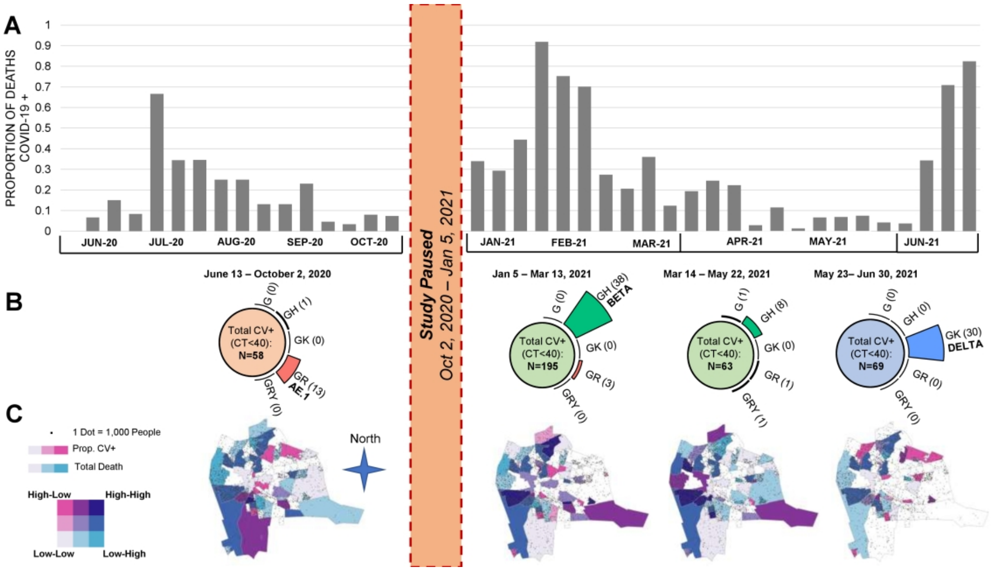

Figure 2. Proportion of COVID-19+ deaths and viral lineages/variants, by time and geography

338x190mm (300 x 300 DPI)

SUPPLEMENTARY DATA

Table S1. Proportion of the deceased who had been tested antemortem, by setting

	Facility	Community	All deaths
Tested antemortem	47.4% (272)	1.8% (10)	282
Not tested antemortem	52.6% (302)	97.2% (534)	836
Total	574	544	1,118

Note: We have elected not to conduct a detailed analysis of the concordance between the off study antemortem (clinical care) and postmortem (research) testing. Our team had no role in how those off study samples were collected, what swabs were used, how staff were trained, what test kits were used over time, what quality control measures were used, and so forth. Hence, we have no provenance over the non-study data and do not vouch for their accuracy.

With that caveat, we identified 35 individuals for whom a positive test result was recorded or reported (25 facility and 10 community deaths). Obviously, this is far fewer than what we observed in our postmortem testing. For the reasons outlined above, we can only hypothesize explanations for this discrepancy. Yet, the simplest explanation is plausible: most of the clinical samples were evaluated using rapid antigen kits, which are far less sensitive than PCR.

Please also note that this Table does not account for the different enrollment ratios between the facility and community deaths, which will under-represent the community deaths by three-fold.

For peer review only

Table S2. Proportion of the deceased who tested positive for CV19 by study month

	Ct>=40 (Neg)	Ct<40 (Pos)	Total	Ct>=40 (Neg)	Ct<40 (Pos)	Total
**Month**	**Unweighted**			**Weighted**		
January	77<br>47.8%	84<br>52.2%	161	108.1<br>49.4%	110.8<br>50.6%	218.8
February	118<br>57.0%	89<br>43.0%	207	118.2<br>57.1%	88.9<br>42.9%	207.2
March	189<br>77.5%	55<br>22.5%	244	173.1<br>76.7%	52.7<br>23.4%	225.8
April	216<br>91.5%	20<br>8.5%	236	176.8<br>92.2%	14.9<br>7.8%	191.7
May	154<br>89.0%	19<br>11.0%	173	154.4<br>92.1%	13.3<br>7.9%	167.7
June	37<br>38.1%	60<br>61.9%	97	29.3<br>27.4%	77.7<br>72.6%	107.0
Total	791<br>70.8%	327<br>29.2%	1,118	759.9<br>68.0%	358.4<br>32.0%	1,118.3<br>100.0%

Row results are N (Top) and % (Bottom) in each cell.

For peer review only

Table S3. Clinical presentations of pediatric CV19 positive deaths

Table S3a. Clinical presentation of COVID-19+ facility deaths in children aged 0-19 years

No.	Age in years	Respiratory Symptoms	Gastrointestinal Symptoms	Fever	Cause of Death*
1	<1	difficulty breathing	diarrhea	X	sepsis
2	<1	cough		X	pneumonia
3	<1	cough, difficulty breathing, shortness of breath	diarrhea, vomiting	X	pneumonia
4	<1	difficulty breathing			sepsis
5	<1	cough, difficulty breathing		X	COVID-19 pneumonia
6	<1	difficulty breathing			sepsis
7	<1	difficulty breathing			pneumonia
8	<1	difficulty breathing			sepsis
9	<1	runny nose			sepsis
10	<1	difficulty breathing			sepsis
11	<1	cough	vomiting		sepsis
12	<1	difficulty breathing		X	sepsis
13	<1		vomiting	X	sepsis
14	<1			X	sepsis
15	<1			X	sepsis
16	<1				sepsis
17	<1	difficulty breathing, shortness of breath		X	sepsis
18	<1			X	sepsis
19	<1			X	sepsis
20	<1	cough, difficulty breathing		X	sepsis
21	<1	difficulty breathing			sepsis
22	<1				hypothermia
23	<1			X	sepsis
24	<1	difficulty breathing			pneumonia
25	<1	cough, difficulty breathing		X	pneumonia
26	1-5	shortness of breath		X	sepsis
27	1-5		diarrhea, vomiting		diarrhea
28	1-5				dehydration
29	1-5		diarrhea	X	malnutrition
30	1-5		diarrhea	X	sepsis
31	1-5			X	respiratory failure
32	6-10				diarrhea
33	6-10	difficulty breathing			sepsis
34	11-15				intracranial abscess

* Cause of death from the official death certificate completed by the UTH medical examiner.

For peer review only

Table S3b. Clinical presentation of COVID-19+ community deaths in children aged 0-19 years

No.	Age in years	Respiratory Symptoms	Gastrointestinal Symptoms	Fever
1	<1	difficulty breathing		
2	<1	cough		X
3	<1	cough, difficulty breathing	vomiting	
4	<1		vomiting	
5	<1			X
6	<1		diarrhea, vomiting	X
7	<1	cough, difficulty breathing, fast breathing, shortness of breath		X
8	<1			
9	<1			
10	<1	cough, fast breathing		X
11	<1		diarrhea, vomiting	X
12	<1	cough, difficulty breathing	diarrhea	X
13	1-5	cough		X
14	1-5		diarrhea, vomiting	
15	1-5		diarrhea, vomiting	X
16	1-5		diarrhea	
17	1-5		diarrhea, vomiting	X
18	1-5			
19	1-5			X
20	1-5		diarrhea	
21	1-5	cough	diarrhea	
22	1-5			
23	1-5	cough, difficulty breathing		
24	6-10			
25	6-10		diarrhea	
26	16-19	difficulty breathing		
27	16-19	cough, difficulty breathing		
28	16-19			X
29	16-19			
30	16-19			

We do not include a cause of death for the community deaths. A death certificate is still issued for each case, but these are not based on information from medical providers and are not considered to be accurate.

For peer review only

Supplementary Table S4. The relationship between age and PCR results and viral load as measured through cycle threshold values (Ct).

Age at death (years)	Ct≥40 (Neg)	Weaker (30≤Ct<40)	Strong-est (Ct<30)	Ratio of Weaker to Strongest	Ct≥40 (Neg)	Weaker (30≤Ct<40)	Strong-est (Ct<30)	Ratio of Weaker to Strongest
	Unweighted				Weighted			
0-19	304<br>82.8	54<br>14.7	9<br>2.5	6.0	203.4<br>79.4	45.8<br>17.9	6.9<br>2.7	6.6
20-39	158<br>66.7	55<br>23.2	24<br>10.1	2.3	184.2<br>67.2	62.3<br>22.7	27.7<br>10.1	2.3
40-59	171<br>74.0	36<br>15.6	24<br>10.4	1.7	190.1<br>75.2	39.4<br>15.6	23.4<br>9.3	1.7
60-79	107<br>58.5	45<br>24.6	31<br>16.9	1.4	119.8<br>56.1	54.8<br>25.7	38.9<br>18.2	1.4
80-99	42<br>52.5	19<br>23.8	19<br>23.8	1.0	55.4<br>52.0	26.1<br>24.5	25.0<br>23.5	1.0
100+	0<br>0.0	1<br>50.0	1<br>50.0	1.0	0<br>0.0	1.6<br>50.0	1.6<br>50.0	1.0
Total	782	210	108	1.9	752.9	230.0	123.5	1.9

*4 had missing age data and 12 had no Ct value (tested antemortem with rapid test).

Row results are N (Top) and % (Bottom) in each cell.

Within each age strata we plot the proportion of decedents with a 'negative' test, defined as Ct >40, or a positive test, stratified by Ct strata. Ct is the inverse of the viral load, hence low Ct equals a stronger PCR result and a higher vial load. What we observe is an age dependent effect on the ratio of weaker to strongest PCR strata. Among younger individuals ≤19 years, the ratio of weaker to strongest PCR results was 6.6:1, after weighting. This ratio declines as a function of increasing age. If we assume that viral load is reflective of pathology, then the higher ratios seen in the youngest group indicate a greater proportion of cases where COVID-19 was a co-incidental finding, whereas those in the older age groups, indicate that COVID-19 was more likely to have been a causal factor in those deaths.

For peer review only

Figure S1. Comparison of death by age distributions for the total deceased cohort, the enrolled cohort, and the CV19 positive enrolled cohort.

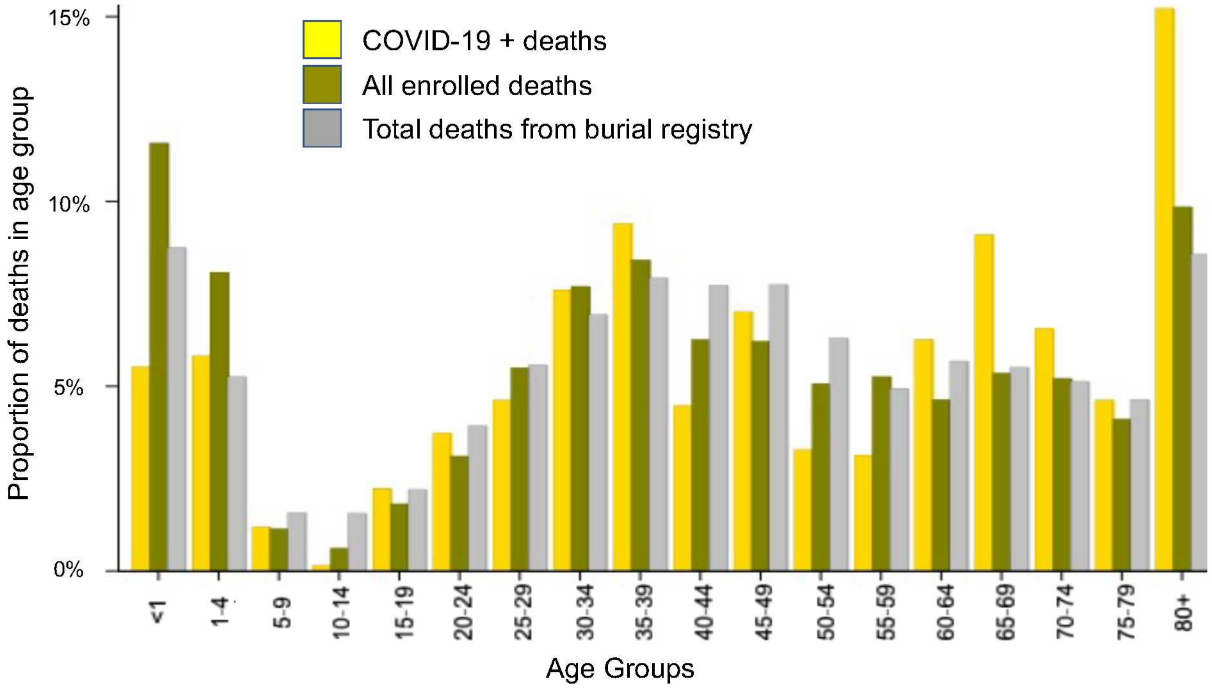

Supplementary Figure S1. Comparison of death by age distributions for the total deceased cohort, the enrolled cohort, and the COVID-19 positive enrolled cohort.

Data for the total death cohort were from Lusaka's burial registry logs maintained by the Zambian Ministry of Health. From each entry, we extracted data of death, age, and sex. We then extracted those deaths that corresponded to the periods of surveillance to capture the age by death distribution for the total cohort (enrolled and unenrolled). The second two sets correspond to the total enrolled cohort (N=1,118 person) and the subset who were COVID-19+ (N=327).

Figure S2. Distribution of cycle threshold values for PCR results targeting the N1 and N2 nucleocapsid proteins.

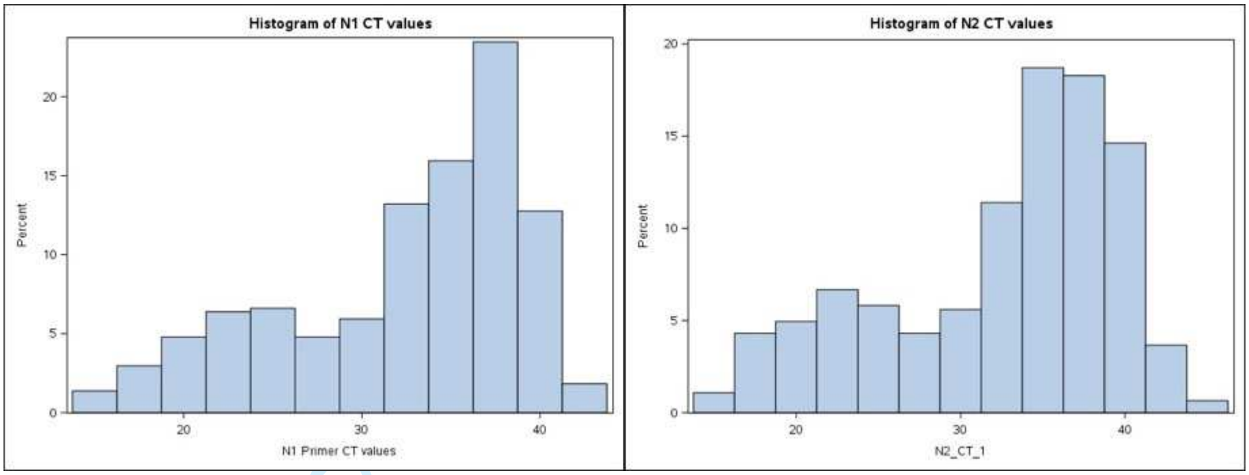

Supplementary Figure S2. Distribution of cycle threshold values for the N1 and N2 PCR reactions

The median Ct results were 32 and 33, respectively for the N1 and N2 targets. Results for detections between >40 and 45 are noted in text but are not included in any of our summary results otherwise.

For peer review only

Supplementary Figure S3

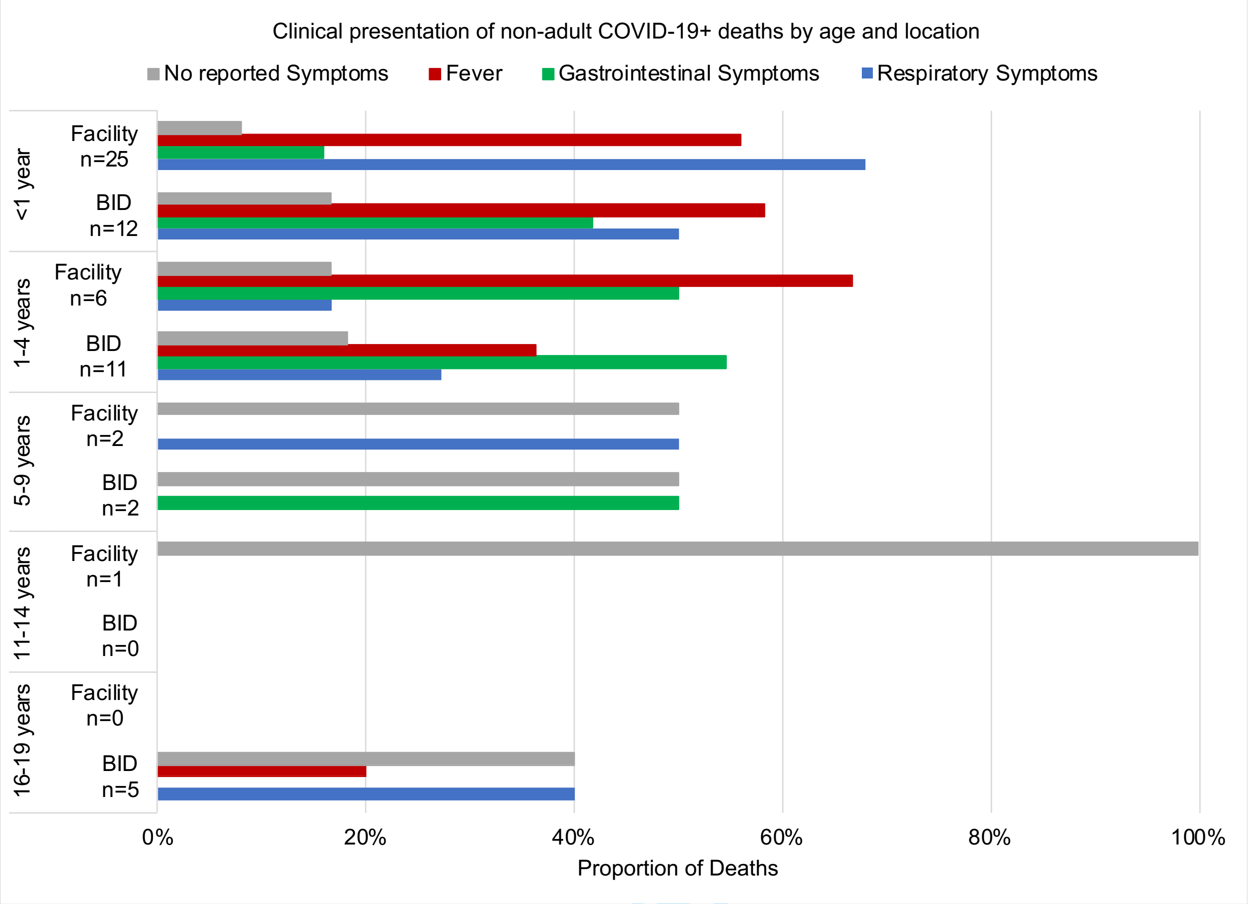

Supplementary Figure S3. Clinical presentations among COVID-19 positive children ≤19 years

For this analysis, we extracted the data about clinical presentation of each case from the medical charts (facility deaths) or verbal autopsy data (community deaths). We then clustered these syndromically into those that were ‘respiratory’ vs. ‘gastrointestinal’ vs. ‘other’. Since ‘fever’ could occur in any of these, or by itself, we summarized this separately. As can be seen, the syndromic presentation of COVID-19+ infants <1 is distinct from that in older children. In the infants, we see a high proportion with gastrointestinal complaints and a relative paucity with respiratory disease. In the older children, this pattern reverses, and in adolescents the pattern is consistent with that seen in adults, i.e., various combinations of respiratory symptoms, often accompanied by fever. Since this is an analysis by individual, there was no need to make weighting adjustments to account for enrollment ratios.

STROBE Statement—Checklist of items that should be included in reports of ***cross-sectional studies***

	Item No	Recommendation
**Title and abstract**	1	(*a*) Indicate the study's design with a commonly used term in the title or the abstract***Done pg 1
		(*b*) Provide in the abstract an informative and balanced summary of what was done and what was found***Done
**Introduction**		
Background/rationale	2	Explain the scientific background and rationale for the investigation being reported*** Done
Objectives	3	State specific objectives, including any prespecified hypotheses*** Done
**Methods**		
Study design	4	Present key elements of study design early in the paper*** Done
Setting	5	Describe the setting, locations, and relevant dates, including periods of recruitment, exposure, follow-up, and data collection*** Done
Participants	6	(*a*) Give the eligibility criteria, and the sources and methods of selection of participants*** Done
Variables	7	Clearly define all outcomes, exposures, predictors, potential confounders, and effect modifiers. Give diagnostic criteria, if applicable*** Done
Data sources/ measurement	8*	For each variable of interest, give sources of data and details of methods of assessment (measurement). Describe comparability of assessment methods if there is more than one group*** Done pg 3
Bias	9	Describe any efforts to address potential sources of bias*** Done pg 5
Study size	10	Explain how the study size was arrived at***Done pg 7
Quantitative variables	11	Explain how quantitative variables were handled in the analyses. If applicable, describe which groupings were chosen and why*** not applicable – we did not do modeling
Statistical methods	12	(*a*) Describe all statistical methods, including those used to control for confounding*** Not applicable to this study design
		(*b*) Describe any methods used to examine subgroups and interactions*** Not applicable, there was no modelling in this analysis
		(*c*) Explain how missing data were addressed*** We did not use imputation so this did not apply.
		(*d*) If applicable, describe analytical methods taking account of sampling strategy*** Not applicable
		(*e*) Describe any sensitivity analyses*** Not applicable
**Results**		
Participants	13*	(a) Report numbers of individuals at each stage of study—eg numbers potentially eligible, examined for eligibility, confirmed eligible, included in the study, completing follow-up, and analysed*** Not applicable -this was a cross sectional study, so attrition does not apply
		(b) Give reasons for non-participation at each stage*** Not applicable
		(c) Consider use of a flow diagram*** Also necessary. This was a prevalence study conducted among postmortem participants. There is no 'flow' to report under this methodology
Descriptive data	14*	(a) Give characteristics of study participants (eg demographic, clinical, social) and information on exposures and potential confounders*** Done pg10

For peer review only

		(b) Indicate number of participants with missing data for each variable of interest*** Not applicable
Outcome data	15*	Report numbers of outcome events or summary measures*** Done pg 9 and actually throughout the results
Main results	16	(*a*) Give unadjusted estimates and, if applicable, confounder-adjusted estimates and their precision (eg, 95% confidence interval). Make clear which confounders were adjusted for and why they were included*** Not applicable. We did not do modelling.
		(*b*) Report category boundaries when continuous variables were categorized***Not applicable
		(*c*) If relevant, consider translating estimates of relative risk into absolute risk for a meaningful time period*** Not applicable. We do not report on relative risks.
Other analyses	17	Report other analyses done—eg analyses of subgroups and interactions, and sensitivity analyses*** not applicable
**Discussion**		
Key results	18	Summarise key results with reference to study objectives*** Done pg 16
Limitations	19	Discuss limitations of the study, taking into account sources of potential bias or imprecision. Discuss both direction and magnitude of any potential bias*** Done pgs 17-18
Interpretation	20	Give a cautious overall interpretation of results considering objectives, limitations, multiplicity of analyses, results from similar studies, and other relevant evidence*** Done. pg 18
Generalisability	21	Discuss the generalisability (external validity) of the study results*** Done pg 18
**Other information**		
Funding	22	Give the source of funding and the role of the funders for the present study and, if applicable, for the original study on which the present article is based*** Done pg 20

*Give information separately for exposed and unexposed groups.

Note: An Explanation and Elaboration article discusses each checklist item and gives methodological background and published examples of transparent reporting. The STROBE checklist is best used in conjunction with this article (freely available on the Web sites of PLoS Medicine at http://www.plosmedicine.org/, Annals of Internal Medicine at http://www.annals.org/, and Epidemiology at http://www.epidem.com/). Information on the STROBE Initiative is available at www.strobe-statement.org.

For peer review only

BMJ Open

What is the prevalence of COVID-19 detection by PCR among deceased individuals in Lusaka, Zambia? A systematic post-mortem study

Journal:	*BMJ Open*
Manuscript ID	bmjopen-2022-066763.R1
Article Type:	Original research
Date Submitted by the Author:	14-Oct-2022
Complete List of Authors:	Gill, Christopher; Boston University School of Public Health, Department of International Health<br>Mwananyanda, Lawrence; Boston University School of Public Health, Research<br>MacLeod, William; Boston University School of Public Health, Global Health<br>Kwenda, Geoffrey; University of Zambia - Ridgeway Campus, Biomedical Sciences<br>Pieciak, Rachel; Boston University School of Public Health, Global Health<br>Etter, Lauren; Boston University School of Public Health, Department of Global Health<br>Bridges, Daniel; Program For Applied Technology In Health (PATH)<br>Chikoti, Chilufya; Avencion Limited<br>Chirwa, Sarah; Avencion Limited<br>Chimoga, Charles; Avencion Limited<br>Forman, Leah; Boston University School of Public Health, Department of Global Health; Boston University School of Public Health<br>Katowa, Ben; Macha Research Trust<br>Lapidot, Rotem; Boston Medical Center, pediatric infectious diseases<br>Lungu, James; Avencion Limited<br>Matoba, Japhet; Macha Research Trust<br>Mwinga, Gift; Avencion Limited<br>Mubemba, Benjamin; The Copperbelt University, Wildlife Sciences<br>Mupila, Zachariah; Avencion<br>Muleya, Walter; University of Zambia School of Veterinary Medicine, Biomedical Sciences<br>Mwenda, Mulenga; Program for Applied Technology in Health<br>Ngoma, Benard; Avencion Limited<br>Nakazwe, Ruth; University of Zambia University Teaching Hospital, Biomedical sciences<br>Nzara, Diana; Avencion Limited<br>Pawlak, Natalie; Tufts University School of Medicine<br>Pemba, Lillian; Avencion Limited<br>Saasa, Ngonda; University of Zambia School of Veterinary Medicine<br>Simulundu, Edgar; Macha Research Trust<br>Yankonde, Baron; Avencion Limited<br>Thea, Donald; Boston University School of Public Health, Global Health
Primary Subject	Global health

Heading:	
Secondary Subject Heading:	Infectious diseases
Keywords:	COVID-19, Epidemiology < INFECTIOUS DISEASES, PUBLIC HEALTH

SCHOLARONE™
Manuscripts

I, the Submitting Author has the right to grant and does grant on behalf of all authors of the Work (as defined in the below author licence), an exclusive licence and/or a non-exclusive licence for contributions from authors who are: i) UK Crown employees; ii) where BMJ has agreed a CC-BY licence shall apply, and/or iii) in accordance with the terms applicable for US Federal Government officers or employees acting as part of their official duties; on a worldwide, perpetual, irrevocable, royalty-free basis to BMJ Publishing Group Ltd ("BMJ") its licensees and where the relevant Journal is co-owned by BMJ to the co-owners of the Journal, to publish the Work in this journal and any other BMJ products and to exploit all rights, as set out in our licence.

The Submitting Author accepts and understands that any supply made under these terms is made by BMJ to the Submitting Author unless you are acting as an employee on behalf of your employer or a postgraduate student of an affiliated institution which is paying any applicable article publishing charge ("APC") for Open Access articles. Where the Submitting Author wishes to make the Work available on an Open Access basis (and intends to pay the relevant APC), the terms of reuse of such Open Access shall be governed by a Creative Commons licence – details of these licences and which Creative Commons licence will apply to this Work are set out in our licence referred to above.

Other than as permitted in any relevant BMJ Author's Self Archiving Policies, I confirm this Work has not been accepted for publication elsewhere, is not being considered for publication elsewhere and does not duplicate material already published. I confirm all authors consent to publication of this Work and authorise the granting of this licence.

For peer review only

TITLE

What is the prevalence of COVID-19 detection by PCR among deceased individuals in Lusaka, Zambia? A systematic post-mortem study

AUTHORS

Christopher J Gill [1], Lawrence Mwananyanda [1,2], William MacLeod [1], Geoffrey Kwenda [3], Rachel Pieciak [1], Lauren Etter [1], Daniel Bridges [4], Chilufya Chikoti [2], Sarah Chirwa [2], Charles Chimoga [2], Leah Forman [5], Ben Katowa [6], Rotem Lapidot [1], James Lungu [2], Japhet Matoba [6], Gift Mwinga [2], Benjamin Mubemba [7], Zachariah Mupila [2], Walter Muleya [8], Mulenga Mwenda [8], Benard Ngoma [2], Ruth Nakazwe [2, 9], Diana Nzara [2], Natalie Pawlak [10], Lillian Pemba [2], Ngonda Saasa [8], Edgar Simulundu [6], Baron Yankonde [2], and Donald M. Thea [1].

Corresponding Author: Christopher J. Gill, MD MS, Professor, Dept. of Global Health, Boston University School of Public Health, cgill@bu.edu

AFFILIATIONS

1. Boston University School of Public Health, Department of Global Health, Boston, Massachusetts, USA
2. Avencion Limited, Lusaka, Zambia
3. University of Zambia, School of Health Sciences, Department of Biomedical Sciences, Lusaka, Zambia
4. Program for Applied Technology in Health (PATH), Malaria Control and Elimination Partnership for Africa, Lusaka, Zambia
5. Biostatistics and Epidemiology Data Analytics Center, Boston University School of Public Health, Boston, Massachusetts, USA
6. Macha Research Trust, Choma, Zambia
7. Copperbelt University, School of Natural Resources, Department of Wildlife Sciences, Kitwe, Zambia
8. University of Zambia, School of Veterinary Medicine, Department of Biomedical Sciences, Lusaka, Zambia
9. University Teaching Hospital, Department of Microbiology, Lusaka, Zambia
10. Tufts University school of Medicine, Boston, Massachusetts, USA

For peer review only - http://bmjopen.bmj.com/site/about/guidelines.xhtml

ABSTRACT

Objectives. To determine the prevalence of COVID-19 post-mortem setting in Lusaka, Zambia.
Design. A systematic, post-mortem prevalence study.
Setting. A busy, inner-city morgue in Lusaka.
Participants. We sampled a random subset of all decedents who transited the UTH morgue. We sampled the posterior nasopharynx of decedents using quantitative PCR. Prevalence was weighted to account for age-specific enrollment strategies.
Interventions. Not applicable – this was an observational study.
Primary outcomes. Prevalence of COVID-19 detections by PCR. Results were stratified by setting (facility vs. community deaths), age, demographics, and geography, and time.
Secondary outcomes. Shifts in viral variants; causal inferences based on Ct values and other features; antemortem testing rates.
Results. From 1,118 decedents enrolled between Jan-June 2021, COVID-19 was detected among 32.0% (358/1,116). Roughly four COVID-19+ community deaths occurred for every facility death. Antemortem testing occurred for 52.6% (302/574) of facility deaths but only 1.8% (10/544) of community deaths and overall, only ~10% of COVID-19+ deaths were identified in life. During peak transmission periods, COVID-19 was detected in ~90% of all deaths. We observed three waves of transmission that peaked in July 2020, January 2021, and ~June 2021: the AE.1 lineage and the Beta and Delta variants, respectively. PCR signals were strongest among those whose deaths were deemed ‘probably due to COVID-19’, and weakest among children, with an age-dependent increase in PCR signal intensity.
Conclusions. COVID-19 was common among deceased individuals in Lusaka. Antemortem testing was rarely done, and almost never for community deaths. Suspicion that COVID-19 was the cause of deaths was highest for those with a respiratory syndrome and lowest for individuals <19 years.

For peer review only

Strengths

- We present results obtained systematically in the post-mortem setting; such prevalence data have rarely been reported elsewhere.
- Our results provide rich-meta data allowing us to describe COVID-19 prevalence over time, by city ward, setting of death (community vs. facility), and age stratum.
- We were able to overlay genetic sequencing data, allowing us to observe changes in viral variants over time.

Limitations

- The causal role of COVID-19 infection was not obvious in all cases, thus we can only report the prevalence of COVID-19 among deceased individuals, acknowledging that some may have died with, rather than due, to COVID-19.
- Our data all come from one city in one country, so we cannot directly generalize our results.

For peer review only

INTRODUCTION

Despite the evident harm from the COVID-19 pandemic globally, accurate data about the pandemic's impact in Africa remain sparse. A recent multi-site data identified the severe impact of COVID-19 in terms of deaths in African intensive care units. However, the ICU population is not broadly generalizable, and such observations do not yield a direct estimate of the overall prevalence of COVID-19 or its impact at a population level.[1] In early 2021, our team reported results from systematic COVID-19 post-mortem surveillance conducted from June to October 2020 at the central morgue in Lusaka, Zambia.[2] Using polymerase chain reaction (PCR), we detected COVID-19 in nearly 20% of decedents, most of whom had died in the community. In that group, none had been tested for COVID-19 prior to death. Yet, even among facility deaths, antemortem testing was uncommon. Hence, COVID-19+ deaths appeared rare in Lusaka because testing was rarely done in life. We theorized that the so-called 'Africa Paradox'[3-8] was a myth born from insufficient surveillance data. Other groups have since reached similar conclusions.[9-12]

Our analysis yielded other provocative findings. In contrast with the US and other high-income country (HIC) settings where deaths are concentrated in the elderly, COVID-19+ deaths in Lusaka occurred more evenly across the age spectrum. Further, 10% of COVID-19+ deaths occurred in children, a surprising finding given how rare pediatric COVID-19 deaths are in HICs. It remains an open question whether these pediatric deaths were caused by COVID-19 or whether the virus was a coincidental finding.

These conclusions were conservative given that our analysis rested on only seventy COVID-19+ decedents. Subsequently, our team conducted a second round of post-mortem COVID-19 surveillance, spanning the period January through June 2021. In this longer and larger study, we again quantified the burden of fatal COVID-19 by setting (community vs. facility deaths), calendar date, and geography; and observed the impact and patterns of clinical presentation among children vs. adults. In addition, we aligned our prevalence data with genetic sequencing to characterize shifts in viral lineages/variants over time.

METHODS

Overview

Our COVID-19 surveillance work builds upon a larger body of postmortem surveillance work initiated to define the fatal burden of Respiratory Syncytial Virus and *Bordetella pertussis*. With the onset of the pandemic, the presence of this established infrastructure and team allowed us to quickly amend our protocols to shift from an exclusive focus on RSV and pertussis in infants to measure COVID-19 prevalence across all ages. The RSV results have been published,[13-15] and the pertussis results are currently under review.

We direct the readers to our prior publication for complete details of the surveillance methods.[2] Throughout this paper, we refer to several rounds of surveillance. Round 1 ran from June-Oct 2020; Round 2, comprising the new data in this paper, ran from Jan-June 2021. Round 3 is ongoing, was initiated early in 2022, and is planned to run

For peer review only - http://bmjopen.bmj.com/site/about/guidelines.xhtml

through January 2023. These surveillance windows were the solely due to funding and not based on assumptions about COVID-19 prevalence over time.

The research was conducted with the approval of the ethical review boards from Boston University (reference number H-36469) and the University of Zambia (reference number 015-11-18); written informed consent was obtained from the next of kin or family representative in all cases.

Sampling strategy

When capturing a high proportion of all deaths, postmortem surveillance is robust against ascertainment biases and provides a direct and unambiguous measure of a disease's prevalence, though inferring causality due to the disease may still be challenging. Given concerns about viral RNA degradation leading to false negative results, enrollment was restricted to deaths within the preceding 48 hours.

We enrolled deceased individuals at the University Teaching Hospital (UTH) morgue. UTH is the main teaching hospital for the University of Zambia Medical School. Its morgue is the largest in the city and where ~80% of decedents are passed on to their families, irrespective of socio-economic status of the decedent. This, plus the legal requirement to obtain a burial certificate before interring a body, most of which are issued at the UTH morgue, makes it highly representative of all deaths in Lusaka.

Due to the high volume in the morgue and finite capacity of our team, we capped enrollments at ~5-6 deaths per day. Also due to high volume, we enrolled community deaths at a 1:3 ratio, while enrolling the less common facility deaths at a 1:1 ratio. In response to the surprising high proportion of pediatric deaths in surveillance Round 1, we elected to also oversample this group enrolling infants (<1 year) at a 1:1 ratio. This required that we adjust for these ratios in our total population prevalence estimates (see statistical analysis). Our team had no access to the clinical data about each decedent and could not know the PCR results prior to consent and enrollment. Therefore, we were confident that our enrolment strategy was robust against selection biases with respect to COVID-19 status.

Concurrently, we extracted total deaths by age stratum from the official government burial registry. This allowed us to estimate the contemporaneous proportion of all deaths represented by our sample, and, by comparing the age by death distribution of the two groups, assess whether the enrolled sample was representative of the total.

Data collection and case definitions

We defined a 'facility death' as one that occurred under care at UTH or a referring facility. We defined a community death as one that occurred outside of medical care. We defined pediatric deaths as those occurring between 0-≤19 years, recognizing that some may choose to define the end of adolescence differently, and that such definitions may not align with official age of emancipation laws in different parts of the world.

Following enrollment, individual and household demographic data were collected on all decedents, along with any antemortem COVID-19 testing results that were documented in the medical chart (for facility deaths) or reported by the next of kin/representative of the decedent during the verbal autopsy (for community deaths).

For peer review only - http://bmjopen.bmj.com/site/about/guidelines.xhtml

The most difficult challenge was inferring the causal role of COVID-19 in each death: the SARS-CoV-2 virus may have a direct or indirect role in deaths or could be a coincidental finding. The US CDC has issued guidance stating that in deaths where COVID-19 is detected, the virus should be assumed to be the direct or underlying cause of death, absent exonerating circumstances.[16] That notwithstanding, to better infer the causal role of COVID-19 in deaths, we used the clinical data describing symptoms during the fatal event to categorize the syndromic presentation of each death. For facility deaths, these data were obtained from a medical chart extraction focusing on symptoms at initial presentation to UTH; for community deaths, we conducted a verbal autopsy using the abbreviated tool validated by the Institute for Health Metrics Evaluation (IHME).[17] From these syndromic data, using the same approach as in our initial publication,[2] we classified COVID-19 PCR+ adult decedents (>19 years) into the following categories:

1) Probable COVID-19 deaths, where the individual had presented with any combination of fever and/or respiratory symptoms.
2) Possible COVID-19 deaths, where the individual presented without classic respiratory symptoms, but rather with known sequalae of COVID-19 suggestive of a heart attack, stroke, or other acute vascular event related to COVID-19's propensity to increase the risk of clotting disorders.[18]
3) Probably not COVID-19 deaths, where the individual had an exonerating cause of death suggesting that the COVID-19 finding on PCR was co-incidental.
4) Unknown, where the data were insufficient to adjudicate.

The reasons we only applied this strategy to adults is that this taxonomy quickly proved ill-suited for younger individuals. Unlike adults, where fever and/or respiratory symptoms predominated, the pediatric presentations were more variable and respiratory symptoms less common. Since we were (and remain) uncertain about how COVID-19 should present clinically in children, we worried that creating *a priori* case definitions based on how the virus behaves in adults could be misleading. Similarly, applying a new definition developed iteratively from the constellation of symptoms observed in children and then applying that definition forwards without validation against an external gold standard (such as histopathology coupled with PCR), constitutes a logical circularity.

Facing this uncertainty, we opted simply to describe the pediatric case presentations without attempting to infer causality for decedents aged $\leq$19 years.

Biological sample collection

From each deceased individual, we obtained posterior nasopharyngeal (NP) samples. We used flocked-tipped NP swabs (Copan Diagnostics, Murietta, CA, USA),[19 20] inserting the swab into both nares and then rotating 180 degrees in both directions and collected into 3mL vials of universal transport media. Samples were stored cold or on ice until processing at our onsite molecular lab.

For peer review only - http://bmjopen.bmj.com/site/about/guidelines.xhtml

Laboratory procedures

PCR testing for COVID-19

After vortexing to elute samples, total nucleic acid for PCR was extracted using the EasyMAG system (Biomerieux, Marcy L'Etoile, France).[21] We ran PCR for 45 cycles using the US CDC's COVID-19 testing kit, which targets the N1 and N2 nucleocapsid proteins.[22] We also ran PCR on each sample against the constitutive human enzyme RNAseP. The presence of RNAseP indicates that the NP swab made effective contact with the respiratory mucosa and that there were no PCR inhibitors.

Per the instructions in the CDC's protocol, we defined a COVID-19 test result as positive if the PCR cycle threshold (Ct) was <40 for both the N1 and N2 genes, provided that each assay demonstrated a logarithmic fluorescence amplification curve, had RNAseP detectable at Ct<40, and had positive and negative plate controls performing as anticipated.

However, Ct is a continuous variable, and the selection of any Ct cut point is arbitrary and hence controversial.[23] While a lower Ct, reflecting a higher viral load, is often associated with increased clinical severity, most COVID-19 surveillance studies assess individuals when they present acutely. In the acute setting, viral loads, and hence PCR signals, are likely at peak intensity. By contrast, the population in this study were all tested after they had died, and currently there are few data about what PCR signals should look like at the end of an arc of infection. At that point, the viral load and PCR signal intensity could have declined (or be undetectable), even if COVID-19 had set in motion a set of events culminating in death. For this reason, we accepted a Ct <40 as positive, even though lower Ct values have often been used for defining COVID-19 status at the time of initial presentation. But since this substitutes one arbitrary cut point (<40) for another arbitrary cut point (<30), we also report the number of decedents with COVID-19 detectable between Ct >40 to ≤ 45, though we have not included these in our summary analyses.

Genetic sequencing of selected samples

To characterize the distributions of novel COVID-19 variants, we conducted genetic sequencing to detect viral variants on the subset of samples with a Ct <30. Prior experience has shown that nucleic acid concentrations are rarely sufficient for sequencing above this threshold. For this, we tested samples both from surveillance Rounds 1 and 2, describing shifts in dominant COVID-19 variants in Lusaka over the cumulative period of surveillance.

Total RNA was re-extracted from vortexed NP swab specimens using the QIAamp® Viral RNA Mini Kit (Qiagen, Hilden, Germany) as prescribed by the manufacturer. Complementary DNA was synthesized using LunaScript® RT SuperMix Kit (New England Biolabs, Ipswich, Massachusetts, USA) according to the manufacturer's protocol and multiplex PCR was conducted using custom primers, which were designed using Geneious software version 10.0.9, as used previously to sequence the first COVID-19 case in Zambia using the Sanger method.[24 25] The multiplex PCR generated overlapping amplicons which were then sequenced on the MinION (Oxford Nanopore Technology, United Kingdom) platform. The samples were multiplexed using the Oxford

For peer review only - http://bmjopen.bmj.com/site/about/guidelines.xhtml

Nanopore native barcoding expansion kits 1–12 and 13–24 in combination with the ligation sequencing kit 109 (Oxford Nanopore). The data generated through the MinION was processed using the standard ARTIC bioinformatic pipeline (https://artic.network/ncov-2019/ncov2019-bioinformatics-sop.html). After removal of sequencing primers, the consensus sequences were deposited in the GISAID database (https://www.gisaid.org/). PANGO lineages were then determined for each genome using Pangolin v3.1.16 (https://pangolin.cog-uk.io/).

Statistical analysis

Given the uncertainties of future detection rates for COVID-19, our sampling was not based on *a priori* power assumptions. Rather, we sought to enroll as many decedents as our team could reasonably accommodate. Our statistical analysis was a straightforward comparison of proportions that did not require statistical modeling or imputation. COVID-19 status, based on PCR, was stratified by setting (community vs. facility deaths), by age groups, by calendar date, and by geography (city ward).

Where indicated, we have adjusted prevalence estimates by calculating weighted outputs to account for the different sampling ratios used during enrolment. We calculated weights so that the sum of the weighted population would sum to the sampled population: unweighted sample (1,118) = weighted sample (1,118). This allowed us to calculate the relative frequency of our unweighted sample in the four weighted groups: (1) facility deaths < 1 year, (2) all other facility deaths, (3) community deaths < 1 year of age, and (4) all other community deaths. We sampled the facility deaths < 1 year, all other facility deaths, and community deaths in infants < 1 year at a 1:1 ratio, and the community deaths ≥ 1 year at a 1:3 ratio. We then calculated the weighted sample by multiplying the inverse of the sampling ratio and calculated the relative frequency of the weighted sample. Setting the weighted sample size at the actual enrolled sample size of 1,118, yielded sample weights of 0.53 for the portion of the sample that we recruited as 1:1 and the sample weight of 1.60 for the portion of the sample that we recruited as 1:3. Hence, the ratio of these sample weights, adjusting for rounding, is 1.60/0.53 =~3:1. The reason for this weighting should be self-evident, since without such weighting the prevalence estimates will necessarily over or under estimate the true prevalence across different groups of decedents.

For analyses of COVID-19 deaths over time and place and for the genetic sequence data, we combined results from Rounds 1 and 2 of surveillance. Effectively this provides a time series analysis over a 1-year period minus the 3-month gap from October 2020-Jan 2021 defined by the end and start of adjacent funding cycles. Where appropriate, we reference our results from Round 1 to compare and contextualize results from Round 2. For all statistical analyses and most data manipulations, we used SAS software (Cary, North Carolina, USA).

For the geospatial analyses, we used ArcGIS software (ESRI, Redlands, California, USA). We pulled population size data from the Zambia Data Hub: https://zambia-open-data-nsdi-mlnr.hub.arcgis.com/, which is managed by the Government of Zambia through the Ministry of Lands and Natural Resources, and the Zambia Statistics Agency. We downloaded these ArcGIS layers to the level of Lusaka's city wards. We

For peer review only - http://bmjopen.bmj.com/site/about/guidelines.xhtml

then created a 2-dimensional heat map that indexed the total number of COVID-19+ deaths against total enrolled deaths across each of Lusaka's main city wards.

Patient and Public Involvement

The participants in this study were all deceased. There was no involvement of patients or the public in the design of this study. The results of this research have already been made publicly available via a posting on MedRXiv.

RESULTS

Study overview and summary of PCR results

This second round of surveillance spanned January-June 2021. The newly enrolled cohort included 1,118 deceased individuals ranging in age from <1 to 102 years. Contemporaneously, 6,270 deaths were reported in the Lusaka burial registries. Thus, our sample represented 17.8% of all deaths in this period. The age distribution of the enrolled vs the total deceased population were similar, arguing to representativeness (**Supplementary Figure S1**).

PCR was successfully conducted on 100% of the NP swabs, and 1,116 had an RNAseP <40, indicating adequacy of sample collection. COVID-19 was detected among 29.3% (327/1,116) of decedents without weighting, and 32.0% (effective sample size = 358/1,116) after adjusting for weighting (**Table 1**).

Table 1. Postmortem COVID-19 PCR results, stratified by setting

Setting of death	Negative, CT≥40	Positive, CT<40	Totals
**Unweighted**			
**Community % (n/N)**	66% (358/543)	34% (185/543)	49% (543/1,116)
**Facility % (n/N)**	75% (431/573)	25% (142/573)	51% (573/1,116)
**Both settings combined**	71% (789/1,116	29% (327/1,116)	100% (1,116/1,116)
**Weighted**			
**Community % (n/N)**	65% 528.2	35% 282.7	72.7% 811
**Facility % (n/N)**	75% 229.5	25% 75.6	27.3% 305
**Both settings combined**	68% (757.7/1,116)	32% (358.3/1,116)	100% (1,116/1,116)

Thus, the overall prevalence of COVID-19 in Round 2 was roughly double what we observed in Round 1 (16%, 58/364). The median Ct values for the N1 and N2 targets were ~33 in both cases (**Supplementary Figure S2)**. We note that an additional 58 samples (45.8 weighted) had COVID-19 detectable at a Ct ≥40-45 but were not included in subsequent analyses.

For peer review only - http://bmjopen.bmj.com/site/about/guidelines.xhtml

The weighted proportion of females among the COVID-19+ and COVID-19- groups was nearly identical (40.6%, 453/1,116). By contrast, the COVID-19+ decedents were significantly older than those without COVID-19 (median 48 years (IQR 30-70 years) vs. 39 years (IQR 0-58 years)), Wilcoxon Ranked Sum test *p*-value < 0.001 (**Table 2**)).

Table 2. Basic demographics of the postmortem cohort, stratified by COVID-19 status

	**Negative, CT≥40**	**Positive, CT<40**	**All Deaths**
**Demographic characteristic**	(n=789)	(n=327)	(n=1,116)
**Unweighted percent Females (n/N)**	42.3% (334/789)	41.0% (134/327)	41.9% (468/1,116)
**Weighted percent female (n/N)**	41.0% 309/758	39.9% 144/358	40.6% 453/1,116
**Unweighted median age at death, years (IQR)**	32.5 (0 - 54)	44.0 (27 - 68)	36.0 (1 - 58)
**Weighted median age at death (IQR)**	39.0 (10-58)	48 (30-70)	41 (23-64)

Proportion of deaths and COVID-19+ deaths by facility vs. community setting

Within the 1,116/1,118 (99.8%) of total deaths with a valid test result, there were 573 facility deaths and 543 community deaths. While this implies that the ratio of facility to community deaths was similar, this does not account for the different enrollment ratios applied to each group. After weighting, 73% of all deaths occurred in the community vs. 27% at a facility, for a ratio of ~three community deaths for each COVID-19+ facility death.

Similarly, COVID-19 was detected among 24.8% (142/573) of facility deaths and 34.0% (185/543) of community deaths. Considering the contribution of deaths by setting, among the 327 COVID-19+ decedents, 56.6% (185/327) were community deaths and 43.4% (142/327) were facility deaths. After weighting to adjust for enrollment ratios, community deaths accounted for 79% and facility deaths for 21% of total COVID-19+ deaths. Stated another way, for each COVID-19+ decedent detected at a facility, ~four COVID-19+ decedents were identified in the community.

COVID-19 deaths by age group

During Round 1, the proportion of COVID-19+ individuals was similar across all age groups and 10% of all COVID-19+ deaths were in children ≤19 years.

Round 2 yielded a similar pattern with COVID-19+ deaths occurring across all age groups and with relatively similar proportions out of total deaths in each group (see **Figure 1**). After weighting, adults aged >19 years comprised 86.2% (300/358) of COVID-19+ deaths and children 0 to ≤19 years for 14.9% (53/358) (**Table 3**).

For peer review only

Table 3. Distribution of COVID-19 positive deaths by 20-year increments

Age at death (years)	Ct>=40 (Neg)	Ct<40 (Pos)	Total	Ct>=40 (Neg)	Ct<40 (Pos)	Total
	**Unweighted**			**Weighted**		
0-19	308<br>82.8%	64<br>17.2%	372	205.6<br>79.4%	53.3<br>20.6%	261.0
20-39	159<br>66.5%	80<br>33.5%	239	184.8<br>67.1%	90.5<br>32.9%	275.3
40-59	171<br>73.4%	62<br>26.6%	233	190.1<br>74.8%	63.9<br>25.2%	253.9
60-79	108<br>58.1%	78<br>41.9%	186	120.3<br>55.9%	94.8<br>44.1%	215.1
80-99	42<br>52.5%	38<br>47.5%	80	55.4<br>52.0%	51.1<br>48.0%	106.5
100+	0<br>0.0%	2<br>100.0%	2	0<br>0.0%	3.2<br>100.0%	3.2
Total	788*	324*	1,112*	757.7*	358.4*	1,112.1*

*4 had missing age data, of which 3 were COVID-19 positive and 1 was COVID-19 negative, for 789, 327 and 1,116, respectively
Row results are N (Top) and % (Bottom) in each cell.

Overall, 78.1% (208/358, weighted) of COVID-19+ decedents were aged <60 years. Contrasting the death by age distributions for the COVID-19+ decedents relative to the age distributions for the total population from the burial registries, the COVID-19+ deaths were relatively underrepresented among children and relatively over represented in the elderly. Outside of these age extremes, the age by death distributions for the two cohorts were similar (**Supplemental Figure S1**).

Antemortem testing for COVID-19

During Round 1, antemortem testing for COVID-19 was rarely done, occurring in only 10% of facility deaths and none of the majority of deaths that were in the community.

In Round 2, antemortem testing rates improved but only among facility deaths. Within this group, 52.6% (302/574) were tested antemortem, of whom 25 were reportedly positive (see **Supplementary Table S1**). During this period, nearly all COVID-19 testing at UTH relied on rapid antigen tests. However, among the community deaths, we were still only able to document antemortem testing for 1.8% (10/544) of COVID-19+ decedents.

Temporal shifts in COVID-19 prevalence, dominant variants, and geography

Given these changes between Round 1 and Round 2, we further explored the relationship between total deaths, COVID-19 prevalence, viral lineages, and geographic

For peer review only - http://bmjopen.bmj.com/site/about/guidelines.xhtml

distribution in time series analyses. These results, combining Round 1 and 2 data, are presented in **Figure 2**.

Several features are noteworthy. First, the data collected in Round 1 document what in hindsight was likely the first, and smallest, of three waves of COVID-19 to occur in Lusaka during our surveillance period (**Panel A**). Noting the gap in surveillance (between adjacent funding cycles) from October 2020 to January 2021, wave 1 occurred in June-October 2020; wave 2 in January-February 2021; and wave 3 in May-June 2021. Round 2 surveillance ended in June 2021, precluding observations about when wave 3 peaked and receded.

Second, during waves 2 and 3, the proportion of decedents who tested positive for COVID-19 peaked at 91.7% prevalence in January and 83.8% in June of 2021. Both represented a statistically significant increase over the average prevalence during Rounds 1 and 2 (ANOVA, P<0.001). The proportion of deceased who tested positive for COVID-19 by month are summarized in **supplementary Table S2**.

Third, each wave presented a distinct pattern in terms of prevalent viral lineages (**Panel B**). Combining Rounds 1 and 2 and selecting those with a Ct<30, we successfully sequenced 96 isolates. From these, lineage AE.1 was dominant during wave 1. During wave 2 in Jan-Feb, the B.1.351 variant (the Beta variant) was dominant. Beta was supplanted by the emergence of the B1.617 variant (Delta) during wave 3, which accounted for 100% of all sequenced isolates during this period.

Concurrently, we mapped the distribution of COVID-19+ cases indexed against total deaths over time across Lusaka's major city wards (**Panel C**). The areas shaded dark blue represent areas where COVID-19+ deaths were disproportionately high relative to total deaths, while those in light pink, light blue, or white are areas where COVID-19+ deaths were underrepresented relative to total deaths. Within this taxonomy, the highest burden of COVID-19 was in Lusaka's poorest and most densely populated areas that constitute the peripheral ring of peri-urban compounds to the city's north, west, and south, and largely spared the more affluent neighborhoods that cluster in the center of the city.

Clinical presentation of COVID-19 positive children and adolescents

Recalling that infants <1 year were intentionally oversampled, requiring weighting to draw overall prevalence estimates, most deaths in children and adolescents ≤19 y were in infants. With that caveat, **Supplementary Figure S3** summarizes the syndromic presentations for the COVID-19 positive deceased children <19 years, stratifying these by age groups and community/facility settings. Since we are describing these on a per individual basis, there was no need to weight these results.

The syndromic categorizations are not mutually exclusive. Clinical presentations appeared to vary by age and setting. Among infants who died at a facility, respiratory symptoms and fever predominated, whereas fever, respiratory and gastrointestinal symptoms occurred in similar proportions among those who died in the community. Among children 1-4 years, fever and gastrointestinal symptoms were more common than respiratory symptoms. Very few COVID-19+ deaths were detected among children aged 5-14 years (N=5). Among children 16-19 years (N=5, all from the community), the

For peer review only - http://bmjopen.bmj.com/site/about/guidelines.xhtml

only reported symptoms were fever and respiratory distress, consistent with COVID-19's presentation in adults. In both community and facility settings, there were several children with no reported symptoms, though this may simply reflect a lack of documentation. Acknowledging the sparse data from older children, these results are broadly consistent with our observations in Round 1 where we had noted the relative infrequency of respiratory symptoms and commonality of gastrointestinal symptoms, particularly among children ≤ 5 years. **Supplementary Table S3** provides a detailed line listing summarizing the clinical presentation of each COVID-19+ pediatric death by setting (facility deaths **Table S3a**, community deaths **Table S3b**).

Inferring the causal role of COVID-19 in these deaths

During Round 1, among those who had sufficient clinical data to allow an assessment of causality, nearly all were judged to have 'probably' or 'possibly' died from COVID-19.

In Round 2, 89.3% (292/327) of deaths had sufficient data for clinical adjudication, leaving 10.7% (45/327) that could not be adjudicated. Excluding the pediatric cases and those with insufficient data for analysis, we were able to adjudicate 219 deceased adults. Of these, 155/219 (70.8%) were considered 'probably' or 'possibly' due to COVID-19, and 29.2% 'probably not' due to COVID-19. When adjusted for weighting, this combined proportion rose to 73.9% (181/244) (see **Table 4**).

We recognized that the categories used for adjudication (probable, possible, probably not) rested on clinical information that may have been incomplete, a particular problem with the verbal autopsy data given that the respondent providing the information was necessarily relaying these data second hand. A gold standard for asserting causality would have been PCR coupled with histology showing end organ damage consistent with SARS-2 infections. Indeed, we had previously published such pathology based on minimally invasive tissue sampling (MITS) among several of the cases here that had been diagnosed antemortem. As reported in that paper, these individuals showed evidence of diffuse organ injury to the lungs, kidneys and liver in patterns consistent with COVID-19 disease.[26] However, the identification of these decedents in life was unlikely done at random but rather based on clinical suspicion, creating the potential for ascertainment bias.

Given these limitations, we conducted additional causal inference analyses around the Ct data for the full cohort, which we felt would be more robust against selection bias. We reasoned that higher viral loads (and hence lower Ct values) should align with increased severity of disease. If so, we hypothesized that decedents deemed 'probable COVID-19', reflecting a high suspicion that COVID-19 was the cause of death, would on average have lower Ct values (reflecting higher viral loads) than those whose death was deemed 'probably not' COVID-19. The data in **Table 4** supported that hypothesis.

For peer review only

Table 4. Causal role of COVID-19 among PCR(+) deceased adults*, >19 years

Role of COVID-19 in death of patient	Median Ct (IQR)	Cases (N)	Percent	Weighted cases (N)	Weighted percent	Cumulative total for cases that could be adjudicated	Cumulative percent (n/N) for cases that could be adjudicated
**Probably due to COVID-19**	31.6 (24.2-35.5)	127	48.7%	142.2	46.6%	142	58.2% (142/244)
**Possibly due to COVID-19**	30.2 (23.2-35.7)	28	10.7%	38.3	12.6%	180	73.8% (180/244)
**Probably not due to COVID-19**	34.9 (28.0-37.3)	64	24.5%	63.9	20.9%	244	100% (244/244)
**Insufficient data to adjudicate causality**	32.5 (23.9-35.5)	42	16.1%	60.7	19.9%	Not applicable	Not applicable

* Clinical presentations of children with COVID-19 (n= 63) were variable and often lacked typical symptoms of respiratory distress. Since we were uncertain how COVID-19 should behave in children, we did not attempt to adjudicate these cases in terms of presumed causality. The results above have been weighted to adjust for the different enrollment ratios

For peer review only - http://bmjopen.bmj.com/site/about/guidelines.xhtml

For peer review only

6

For peer review only

Among those whose deaths were adjudicated as ‘probably’ due to COVID-19, the median Ct values were 31.6 (IQR CI 24.2-35.5) whereas those adjudicated as ‘probably not’ COVID-19 had a median Ct value of 34.9 (IQR 28-37.3), or a ~ten-fold increase in viral load. Thus, COVID-19+ individuals whose clinical data were most suggestive of a COVID-19 illness by presentation had the highest viral loads.

Following this reasoning further, we conducted an analysis exploring the distribution of Ct values by age groups. As noted previously, we were skeptical that COVID-19 was the cause of death in young children, largely because the clinical presentations in children, and especially children <5 years, differed from adults. Therefore, we reasoned that COVID-19 was more likely to be a coincidental finding in children. To test that contention, we stratified the proportion of PCR results by age group sorting them into two groups: those with the strongest signals (i.e., Ct<30) and those with weaker signals (i.e., Ct $30 \leq Ct \leq 40$). We then calculated the ratio of the proportions of weaker to strongest Ct values within each 20-year age stratum. We hypothesized that if COVID-19 was a co-incidental finding, we would observe a higher ratio of weaker to strong results than if COVID-19 was the cause of death. The results shown in **Supplementary Table S4** supported that hypothesis. After adjusting for weighting, the ratio of weaker to stronger Ct results was 6.6:1 for those aged 0-19 years, compared with a ratio of 1.9:1 across all ages combined. With each increase in age stratum, the proportion of weak to strong PCR signals declined in a stepwise fashion, such that for those 80 years and older, the ratio was 1:1. We draw two conclusions from this analysis. First, the results supported our assumption that COVID-19 was likely a coincidental finding among children. Second, by extension, COVID-19 likely played a causal role in the deaths of older individuals.

DISCUSSION

This second round of postmortem surveillance for COVID-19 reinforces and expands upon our previous observations. First, even if we base our conclusions solely on those who were deemed ‘probably due to COVID-19’ based on clinical presentation, our data suggests that COVID-19 had a severe impact in Lusaka with much loss of life. Second, COVID-19+ deaths were concentrated in the community where testing for COVID-19 was absent. Third, rather than being concentrated in the elderly, COVID-19+ deaths were distributed widely across the age spectrum, and most (~80%) were in individuals aged <60 years. Fourth, COVID-19 was frequently identified among children. Among children ≤5 years, gastrointestinal complaints were common and respiratory symptoms comparatively uncommon, and we suspect that COVID-19 was more likely a co-incidental finding in these younger individuals. Lastly, the emergence of the Beta and Delta variants coincided with marked increases in the prevalence of COVID-19+ deaths, reaching ~90% at the peak of those two waves. Conservatively, COVID-19 was identified in 30% of all decedents between January and June 2021.

A consistent finding was the low proportion of decedents with COVID-19 who were diagnosed in life. We hypothesize that this can be explained as follows: 1) the majority of COVID-19+ deaths (~80%) occurred in the community where, with few exceptions, testing was unavailable; 2) among the hospital deaths, only half were tested; and 3) most of that testing done at facilities used rapid antigen tests, which are less sensitive than PCR.[27] Putting these in sequence, from the 1,118 decedents tested in our study

For peer review only - http://bmjopen.bmj.com/site/about/guidelines.xhtml

we identified COVID-19 in 327 (unweighted). Of these, only 25 had been identified antemortem at UTH, or <10% of the total based on PCR. Since these hospital data feed into the national COVID-19 surveillance data, we conclude that the prevalence COVID-19 was systematically and significantly under-represented. While it is certainly possible that individuals in the community might have self-tested for COVID using a rapid test, it is very unlikely that such results would be reported to the government of Zambia and hence fail to be captured in official government reports.

As in our prior analysis, COVID-19+ deaths were concentrated in Lusaka's most densely populated wards where Lusaka's poorest and most vulnerable citizens reside. Access to care is challenging in these communities, and we have described elsewhere how delays in seeking care may contribute to infant deaths in the community.[28] This highlights a sad element to the global COVID-19 narrative. Even within a poor country like Zambia, there is a gradient of impact that appears to fall hardest on those with the least resources.

There are several important distinctions from our findings in Round 1. First, the numbers of COVID-19 deaths and the proportion of deceased individuals who tested positive for COVID-19 both increased substantially. This coincided with the emergence of the Beta and Delta variants.

Second, the proportion of these deaths that were judged as 'probably' or 'possibly' due to COVID-19 declined from ~100% in Round 1 to ~70 % in Round 2. We acknowledge that the assessment of causality was limited by the evidence present in the medical chart (facility deaths) or reported through the verbal autopsy (community deaths). With that caveat, our procedures and tools and field team for collecting these data were the same in rounds 1 and 2, leading us to conclude that this was a real shift and not an artifact of shifting study procedures.

Our data do not provide an explanation for this decline, but several explanations are plausible. One is that earlier population exposure to COVID-19 (following the first wave) might have provided increased resistance to the SARS-CoV2 virus, i.e., the change is explained by naturally acquired herd immunity from prior exposure. COVID-19 vaccines were virtually unavailable during this period, so vaccine-derived immunity cannot explain this. A second possibility is that the Beta and Delta variants dominated the second and third waves of transmission. Both variants exhibited increased transmissibility, which plausibly accounts for the high proportions of deaths with COVID-19 at the peak of each wave. As seen with the Omicron variant, a gain in transmissibility could yield a reciprocal loss of virulence, i.e., the change is explained by virology.[29 30] However, recent epidemiologic and *in vitro* data suggest the opposite, that the Beta and particularly Delta variants were more, not less, pathogenic.[31-33] Thus, we the former explanation is more plausible.

Strengths and limitations

A strength was that our data were collected prospectively, capturing a wide spectrum of ages in community and facility settings and without prior knowledge of clinical presentation or antemortem testing results. While our capacity only allowed us to enroll a subset of all deaths at the morgue, enrollment was conducted without prior knowledge of clinical presentation or PCR status, and thus should not introduce bias affecting

For peer review only - http://bmjopen.bmj.com/site/about/guidelines.xhtml

overall prevalence estimates after adjusting for weighting. And while all came from the UTH morgue, 80% of all deaths in the city transit this facility, making it highly representative of Lusaka at large. Additionally, we were able to combine multiple data elements on the same individual: clinical presentation, molecular testing, geography, and viral lineage, to provide an overall picture of the impact of the COVID-19 pandemic in Lusaka during these periods.

Limitations include that there was a three-month gap in surveillance between Rounds 1 and 2, and that Round 2 ended prior to the resolution of Wave 3 from the Delta variant. These surveillance periods were defined by funding for the research, not by knowledge of COVID-19 prevalence. Our adjudications were necessarily limited by the completeness of clinical data for the facility deaths and by the accuracy of recall from non-medical persons through the verbal autopsy for community deaths. That could lead to misclassification during our adjudications. Our data describe prevalence but cannot provide a case fatality rate since we lack concurrent incidence data for the general population. Lastly, while Round 2 data confirm identify a high proportion of pediatric deaths with COVID-19 and again show that gastrointestinal symptoms are common in children <5 years, we are very reluctant to inferring causality in this age group. In our judgement, resolving this question requires a higher gold standard, such as PCR coupled with histopathology. Our data do not address the impact of COVID-19 in terms of a case or infection fatality rate, but this question is being examined in an ongoing excess mortality analysis to be published separately.

The most challenging aspect of this work was inferring the causal role of COVID-19 in each death. We note that the clinical definitions applied during adjudication are themselves of limited precision. However, these are the same definitions we had used in our original report in The BMJ 2021, and we believe our approach here is defensible for several reasons. First, it is supported by guidance from the US CDC: given a patient who presented with respiratory distress and fever and died with a positive PCR test for COVID-19, it is no great leap of faith to infer that the death was probably due to COVID-19. Second, we had conducted minimally invasive tissue sampling on a subset of decedents who had been identified with COVID-19 in life. As previously published, the pathology from these individuals was consistent with known patterns of COVID-19 disease, with evidence of lung and other organ dysfunction.[26] Additionally, we have shown that viral loads were higher among individuals whose deaths were deemed ‘probably’ due to COVID-19 than those deemed ‘probably not’ due to COVID-19. And we demonstrated that the ratio of weak to strong PCR signals is age dependent, with much higher ratios in those children and adolescents than older age groups. Combined, these results argue that COVID-19 was a causal factor for deaths in older individuals and more likely a co-incidental finding in those ≤19 years. Causality is far less likely for those deemed ‘possibly’ due to COVID-19. But such cases comprised a small proportion of all COVID-19 positive deaths in this analysis.

Conclusions

COVID-19 was highly prevalent in the post-mortem setting in Lusaka during the first half of 2021 reaching ~90% of deceased individuals during peak periods of transmission and 30% prevalence over the six months of observation. Despite increased rates of antemortem testing for COVID-19, there was little commensurate increase in

For peer review only - http://bmjopen.bmj.com/site/about/guidelines.xhtml

antemortem testing for those who died in the community, who constituted a 4:1 majority of COVID-19+ deaths. Overall, only about 10% of COVID-19+ decedents had been identified in life, yielding a ~ten-fold undercount. Considering recent reports demonstrating examples of underreporting of COVID-19 elsewhere, including inferential methods from excess mortality analyses,[9-12 34-39] we believe that our results are more likely typical than exceptional. If so, COVID-19's impact in Africa has been underestimated.

ACKNOWLEDGEMENTS

We wish to thank our team at the Bill & Melinda Gates Foundation for their support and encouragement: Prachi Vora, Padmini Srikanthia, and Keith Klugman.

For peer review only

FIGURE LEGENDS

Figure 1. Enrolled decedents and COVID-19+ deaths by 5-year age strata

Each stacked bar in the histogram shows the total number of enrolled decedents within each age stratum. The portion in yellow represents those who tested positive by PCR (Ct <40) for COVID-19 and those in grey those who tested negative (Ct ≥40). The % of positives is indicated at the top of each column. The totals have been adjusted to account for differing enrolment ratios.

Figure 2. Proportion of COVID-19+ deaths and viral lineages/variants, by time and geography

Panel A. The histograms represent the total number of COVID-19 positive deaths identified during the two rounds of surveillance. The gap from October 2020 to January 2021 represents the end and start of funding cycles. It is apparent that there were at least three waves of transmission in 2020-2021. Since our enrollment strategy shifted between Round 1 and Round 2, we have adjusted the proportions for the Round 2 data by weighting to account for the different enrolment ratios applied by age and setting.

Panel B. Combing the samples corresponding to each of the three waves, and the period in between waves 2 and 3, we clustered the viral lineages as shown on the top half of the figure. As can be seen, wave 1 was dominated by lineage AE.1, wave 2 by the 'Beta' variant, and wave 3 by the 'Delta' variant. For these data, we present the results without adjustments for weighting.

Panel C. Over these same time intervals, we summarize the distribution of cases indexed against total population within the major city wards of Lusaka. The colors correspond to a heat map defined by the union of 'total deaths' in pink and 'COVID-19+' deaths in blue, and therefore provide an indexed metric of COVID-19 deaths against all deaths. Those with darker shading indicate that COVID-19 deaths were disproportionately high relative to all deaths; those that are lightly shaded or white are where COVID-19 deaths were disproportionately low, relative to total deaths. As can be seen, the highest concentration of cases clustered in the peripheral areas of the city, which correspond to the poorest and most densely populated parts of the city. Whereas the wealthier sections, clustering in the middle of Lusaka, were relatively spared. Round 2 results have also been adjusted for enrollment ratios as in Panel A.

For peer review only

For peer review only - http://bmjopen.bmj.com/site/about/guidelines.xhtml

DECLARATIONS

Dissemination of results

Round 2 data were presented to members of the Zambian Ministry of Health and the Zambian Medical Association in July 2021, and the final manuscript was shared with the Zambian National Health Research Authority (NHRA) prior to submission. The results have been presented to our team at the Bill & Melinda Gates Foundation and to representatives from the US CDC and the Swiss Tropical Medicine Institute. These data have been shared with a modeling team at the Imperial College London to support our ongoing excess mortality analyses. Data from Round 1 were published previously in the BMJ.

Funding

The original ZPRIME study and this COVID-19 expansion were made possible through the generous support of the Bill & Melinda Gates Foundation (OPP 1163027). The funders had no role in designing the study; in the collection and analysis of data; or in the decision to submit the article for publication.

Data sharing

Data are available upon reasonable request

Community engagement

Due to the time constraints around the pandemic, we did not engage the community on this project.

Data provenance

The lead authors (CJG and LM) affirm that the manuscript is an honest, accurate, and transparent account of the study being reported; that no important aspects of the study have been omitted; and that any discrepancies from the study as originally planned (and, if relevant, registered) have been explained.

Manuscript contributions

CJG was the principle investigator, conceived the project, secured the funding, assisted with the protocol and led on writing the manuscript; LM was the Zambian PI, assisted with protocol and tool development, and oversaw the field team and study implementation; WBM was the study statistician, developed the analysis plan and conducted the analysis; GK was the technical lead for the PCR lab in Lusaka; RP was the project manager and a research fellow, developed the study proposal, oversaw study management, and contributed to key aspects of data analysis and presentation; LE was a research fellow and contributed to key aspects of data analysis and presentation; RN, Chilufya Chikoti (CC#1) and BY were the PCR technicians for our lab;

For peer review only

222 SC, LP, GM, DN were the field data collection team under team leaders BN and Charles Chimoga (CC#2, unrelated to CC#1), who conducted grief counseling for the families, obtained consent, and collected all of the clinical data and the biological samples: LF supervised creation of the RedCAP data collection tools and oversaw data management, cleaning and retention; RL contributed to data interpretation and analysis; JL provided information technology support to the field team; BK, JM, BM, and WM conducted the genetic sequencing analyses under the supervision of DB and ES; ZM was the PCR lab manager; NP was a research fellow who supported some of the analyses; MM and NS contributed to the genetic sequencing analyses; and DMT was our senior co-investigator who contributed to data analysis and interpretation.

Ethical review:

The research was conducted with the approval of the ethical review boards from Boston University (reference number H-36469) and the University of Zambia (reference number 015-11-18); written informed consent was obtained from the next of kin or family representative in all cases.

Conflict of Interest:

All authors declare that they have no conflicts of interest relevant to this publication.

For peer review only - http://bmjopen.bmj.com/site/about/guidelines.xhtml

REFERENCES

1. African C-CCOSI. Patient care and clinical outcomes for patients with COVID-19 infection admitted to African high-care or intensive care units (ACCCOS): a multicentre, prospective, observational cohort study. *Lancet* 2021;397(10288):1885-94. doi: 10.1016/S0140-6736(21)00441-4 [published Online First: 2021/05/24]
2. Mwananyanda L, Gill CJ, MacLeod W, et al. Covid-19 deaths in Africa: prospective systematic postmortem surveillance study. *BMJ* 2021;372:n334. doi: 10.1136/bmj.n334 [published Online First: 2021/02/19]
3. Zerfu TA, Tareke AA. What could be the potential reasons for relatively low coronavirus disease 2019 (COVID-19) fatality rates in Africa? The case for Ethiopia. *J Glob Health* 2021;11:03057. doi: 10.7189/jogh.11.03057 [published Online First: 2021/04/22]
4. Musa HH, Musa TH, Musa IH, et al. Addressing Africa's pandemic puzzle: Perspectives on COVID-19 transmission and mortality in sub-Saharan Africa. *Int J Infect Dis* 2021;102:483-88. doi: 10.1016/j.ijid.2020.09.1456 [published Online First: 2020/10/04]
5. Lawal Y. Africa's low COVID-19 mortality rate: A paradox? *Int J Infect Dis* 2021;102:118-22. doi: 10.1016/j.ijid.2020.10.038 [published Online First: 2020/10/20]
6. Bamgboye EL, Omiye JA, Afolaranmi OJ, et al. COVID-19 Pandemic: Is Africa Different? *J Natl Med Assoc* 2021;113(3):324-35. doi: 10.1016/j.jnma.2020.10.001 [published Online First: 2020/11/07]
7. Adams J, MacKenzie MJ, Amegah AK, et al. The Conundrum of Low COVID-19 Mortality Burden in sub-Saharan Africa: Myth or Reality? *Glob Health Sci Pract* 2021;9(3):433-43. doi: 10.9745/GHSP-D-21-00172 [published Online First: 2021/10/02]
8. Ntoumi F, Velavan TP. COVID-19 in Africa: between hope and reality. *Lancet Infect Dis* 2020 doi: 10.1016/S1473-3099(20)30465-5 [published Online First: 2020/06/20]
9. Warsame A, Bashiir F, Freemantle T, et al. Excess mortality during the COVID-19 pandemic: a geospatial and statistical analysis in Mogadishu, Somalia. *Int J Infect Dis* 2021;113:190-99. doi: 10.1016/j.ijid.2021.09.049 [published Online First: 2021/09/28]
10. Watson OJ, Alhaffar M, Mehchy Z, et al. Leveraging community mortality indicators to infer COVID-19 mortality and transmission dynamics in Damascus, Syria. *Nat Commun* 2021;12(1):2394. doi: 10.1038/s41467-021-22474-9 [published Online First: 2021/04/24]
11. Kogan NE, Gantt S, Swerdlow D, et al. Leveraging Serosurveillance and Postmortem Surveillance to Quantify the Impact of COVID-19 in Africa. *Clin Infect Dis* 2022 doi: 10.1093/cid/ciac797 [published Online First: 20221005]
12. Lewis HC, Ware H, Whelan M, et al. SARS-CoV-2 infection in Africa: a systematic review and meta-analysis of standardised seroprevalence studies, from January

For peer review only

2020 to December 2021. *BMJ Glob Health* 2022;7(8) doi: 10.1136/bmjgh-2022-008793
13. Gill CJ, Mwananyanda L, MacLeod WB, et al. Infant deaths from respiratory syncytial virus in Lusaka, Zambia from the ZPRIME study: a 3-year, systematic, post-mortem surveillance project. *Lancet Glob Health* 2022;10(2):e269-e77. doi: 10.1016/S2214-109X(21)00518-0 [published Online First: 2022/01/23]
14. Murphy C, MacLeod WB, Forman LS, et al. Risk Factors for Respiratory Syncytial Virus-Associated Community Deaths in Zambian Infants. *Clin Infect Dis* 2021;73(Suppl_3):S187-S92. doi: 10.1093/cid/ciab453 [published Online First: 2021/09/03]
15. Forman LS, Macleod W, Mwananyanda L, et al. Association of Respiratory Syncytial Virus Infection and Underlying Risk Factors for Death Among Young Infants Who Died at University Teaching Hospital, Lusaka Zambia. *Clin Infect Dis* 2021;73(Suppl_3):S180-S86. doi: 10.1093/cid/ciab466 [published Online First: 2021/09/03]
16. Centers for Disease Control and Prevention. Guidance for certifying deaths due to coronavirus disease 2919 (COVID-19). Vital Statistics Reporting Guidance. Hyattsville, Maryland: National Center for Health Statistics, 2020.
17. Serina P, Riley I, Stewart A, et al. A shortened verbal autopsy instrument for use in routine mortality surveillance systems. *BMC Med* 2015;13:302. doi: 10.1186/s12916-015-0528-8 [published Online First: 2015/12/17]
18. Ramlall V, Thangaraj PM, Meydan C, et al. Immune complement and coagulation dysfunction in adverse outcomes of SARS-CoV-2 infection. *Nat Med* 2020;26(10):1609-15. doi: 10.1038/s41591-020-1021-2 [published Online First: 2020/08/05]
19. Van Horn KG, Audette CD, Sebeck D, Tucker KA. Comparison of the Copan ESwab system with two Amies agar swab transport systems for maintenance of microorganism viability. *J Clin Microbiol* 2008;46(5):1655-8. doi: 10.1128/JCM.02047-07 [published Online First: 2008/03/21]
20. Van Horn KG, Audette CD, Tucker KA, Sebeck D. Comparison of 3 swab transport systems for direct release and recovery of aerobic and anaerobic bacteria. *Diagn Microbiol Infect Dis* 2008;62(4):471-3. doi: 10.1016/j.diagmicrobio.2008.08.004 [published Online First: 2008/09/26]
21. Yang G, Erdman DE, Kodani M, et al. Comparison of commercial systems for extraction of nucleic acids from DNA/RNA respiratory pathogens. *J Virol Methods* 2011;171(1):195-9. doi: 10.1016/j.jviromet.2010.10.024 [published Online First: 2010/11/03]
22. Lu X, Wang L, Sakthivel SK, et al. US CDC Real-Time Reverse Transcription PCR Panel for Detection of Severe Acute Respiratory Syndrome Coronavirus 2. *Emerg Infect Dis* 2020;26(8) doi: 10.3201/eid2608.201246 [published Online First: 2020/05/13]
23. Gill CJ, Gunning CE, MacLeod WB, et al. Asymptomatic Bordetella pertussis infections in a longitudinal cohort of young African infants and their mothers. *Elife* 2021;10 doi: 10.7554/eLife.65663 [published Online First: 2021/06/08]

For peer review only

24. Mwenda M, Saasa N, Sinyange N, et al. Detection of B.1.351 SARS-CoV-2 Variant Strain - Zambia, December 2020. *MMWR Morb Mortal Wkly Rep* 2021;70(8):280-82. doi: 10.15585/mmwr.mm7008e2 [published Online First: 2021/02/26]
25. Simulundu E, Mupeta F, Chanda-Kapata P, et al. First COVID-19 case in Zambia - Comparative phylogenomic analyses of SARS-CoV-2 detected in African countries. *Int J Infect Dis* 2021;102:455-59. doi: 10.1016/j.ijid.2020.09.1480 [published Online First: 2020/10/10]
26. Mudenda V, Mumba C, Pieciak RC, et al. Histopathological Evaluation of Deceased Persons in Lusaka, Zambia With or Without Coronavirus Disease 2019 (COVID-19) Infection: Results Obtained From Minimally Invasive Tissue Sampling. *Clin Infect Dis* 2021;73(Suppl_5):S465-S71. doi: 10.1093/cid/ciab858 [published Online First: 2021/12/16]
27. Scheiblauer H, Filomena A, Nitsche A, et al. Comparative sensitivity evaluation for 122 CE-marked rapid diagnostic tests for SARS-CoV-2 antigen, Germany, September 2020 to April 2021. *Euro Surveill* 2021;26(44) doi: 10.2807/1560-7917.ES.2021.26.44.2100441 [published Online First: 2021/11/06]
28. Lapidot R, Larson Williams A, MacLeod WB, et al. Verbal Autopsies for Out-of-Hospital Infant Deaths in Zambia. *Pediatrics* 2021;147(4) doi: 10.1542/peds.2020-1767 [published Online First: 2021/03/06]
29. Johnson AG, Amin AB, Ali AR, et al. COVID-19 Incidence and Death Rates Among Unvaccinated and Fully Vaccinated Adults with and Without Booster Doses During Periods of Delta and Omicron Variant Emergence - 25 U.S. Jurisdictions, April 4-December 25, 2021. *MMWR Morb Mortal Wkly Rep* 2022;71(4):132-38. doi: 10.15585/mmwr.mm7104e2 [published Online First: 2022/01/28]
30. Shuai H, Chan JF, Hu B, et al. Attenuated replication and pathogenicity of SARS-CoV-2 B.1.1.529 Omicron. *Nature* 2022 doi: 10.1038/s41586-022-04442-5 [published Online First: 2022/01/22]
31. Fisman DN, Tuite AR. Evaluation of the relative virulence of novel SARS-CoV-2 variants: a retrospective cohort study in Ontario, Canada. *Cmaj* 2021;193(42):E1619-E25. doi: 10.1503/cmaj.211248 [published Online First: 2021/10/07]
32. Zhang J, Xiao T, Cai Y, et al. Membrane fusion and immune evasion by the spike protein of SARS-CoV-2 Delta variant. *Science* 2021;374(6573):1353-60. doi: 10.1126/science.abl9463 [published Online First: 2021/10/27]
33. Tao K, Tzou PL, Nouhin J, et al. The biological and clinical significance of emerging SARS-CoV-2 variants. *Nat Rev Genet* 2021;22(12):757-73. doi: 10.1038/s41576-021-00408-x [published Online First: 2021/09/19]
34. Walker PGT, Whittaker C, Watson OJ, et al. The impact of COVID-19 and strategies for mitigation and suppression in low- and middle-income countries. *Science* 2020;369(6502):413-22. doi: 10.1126/science.abc0035 [published Online First: 2020/06/14]
35. Russell TW, Golding N, Hellewell J, et al. Reconstructing the early global dynamics of under-ascertained COVID-19 cases and infections. *BMC Med* 2020;18(1):332. doi: 10.1186/s12916-020-01790-9 [published Online First: 2020/10/23]
36. Koum Besson ES, Norris A, Bin Ghouth AS, et al. Excess mortality during the COVID-19 pandemic: a geospatial and statistical analysis in Aden governorate,

For peer review only - http://bmjopen.bmj.com/site/about/guidelines.xhtml

Yemen. *BMJ Glob Health* 2021;6(3) doi: 10.1136/bmjgh-2020-004564 [published Online First: 2021/03/25]

37. Karlinsky A, Kobak D. Tracking excess mortality across countries during the COVID-19 pandemic with the World Mortality Dataset. *Elife* 2021;10 doi: 10.7554/eLife.69336 [published Online First: 2021/07/01]

38. Alves THE, de Souza TA, Silva SA, Ramos NA, de Oliveira SV. Underreporting of Death by COVID-19 in Brazil's Second Most Populous State. *Front Public Health* 2020;8:578645. doi: 10.3389/fpubh.2020.578645 [published Online First: 2021/01/02]

39. Van Gordon MM, Mwananyanda L, Gill CJ, McCarthy KA. Regional comparisons of COVID reporting rates, burden, and mortality age-structure using auxiliary data sources. *MedRxiv (preprint)* 2021 doi: 2021.08.18.212622482021

For peer review only

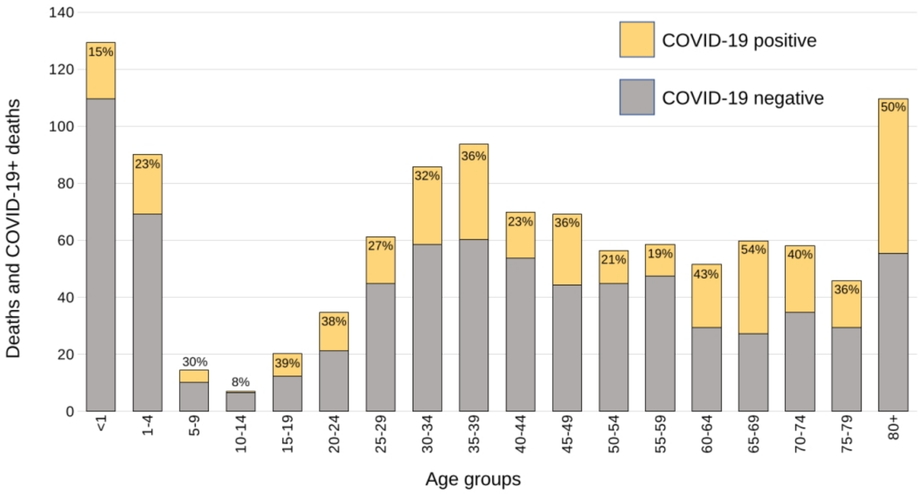

Figure 1. Enrolled decedents and COVID-19+ deaths by 5-year age strata

37058x20828mm (1 x 1 DPI)

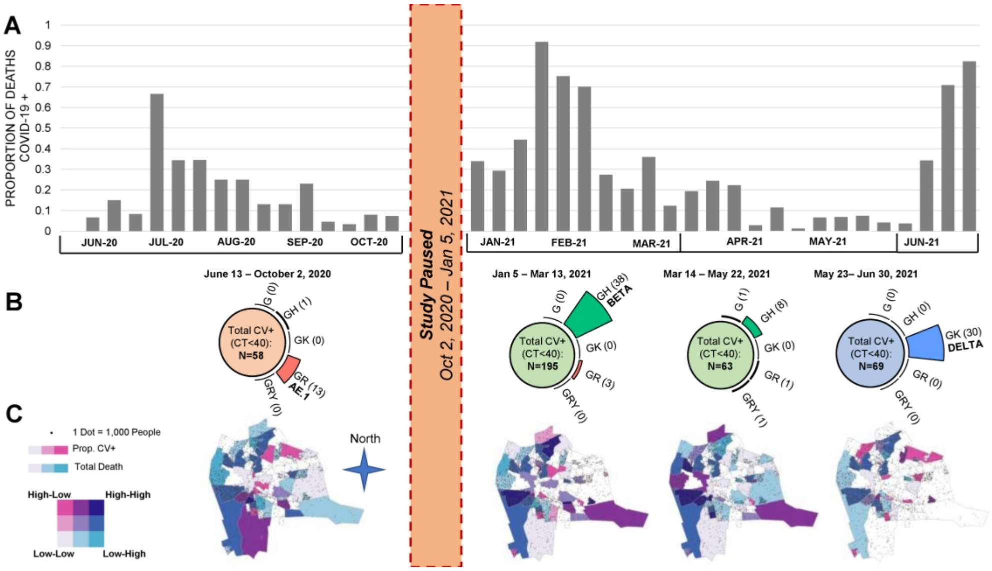

Figure 2. Proportion of COVID-19+ deaths and viral lineages/variants, by time and geography

338x190mm (300 x 300 DPI)

SUPPLEMENTARY DATA

Table S1. Proportion of the deceased who had been tested antemortem, by setting

	Facility	Community	All deaths
Tested antemortem	47.4% (272)	1.8% (10)	282
Not tested antemortem	52.6% (302)	97.2% (534)	836
Total	574	544	1,118

Note: We have elected not to conduct a detailed analysis of the concordance between the off study antemortem (clinical care) and postmortem (research) testing. Our team had no role in how those off study samples were collected, what swabs were used, how staff were trained, what test kits were used over time, what quality control measures were used, and so forth. Hence, we have no provenance over the non-study data and do not vouch for their accuracy.

With that caveat, we identified 35 individuals for whom a positive test result was recorded or reported (25 facility and 10 community deaths). Obviously, this is far fewer than what we observed in our postmortem testing. For the reasons outlined above, we can only hypothesize explanations for this discrepancy. Yet, the simplest explanation is plausible: most of the clinical samples were evaluated using rapid antigen kits, which are far less sensitive than PCR.

Please also note that this Table does not account for the different enrollment ratios between the facility and community deaths, which will under-represent the community deaths by three-fold.

For peer review only

Table S2. Proportion of the deceased who tested positive for CV19 by study month

	Ct>=40 (Neg)	Ct<40 (Pos)	Total	Ct>=40 (Neg)	Ct<40 (Pos)	Total
**Month**	**Unweighted**			**Weighted**		
January	77<br>47.8%	84<br>52.2%	161	108.1<br>49.4%	110.8<br>50.6%	218.8
February	118<br>57.0%	89<br>43.0%	207	118.2<br>57.1%	88.9<br>42.9%	207.2
March	189<br>77.5%	55<br>22.5%	244	173.1<br>76.7%	52.7<br>23.4%	225.8
April	216<br>91.5%	20<br>8.5%	236	176.8<br>92.2%	14.9<br>7.8%	191.7
May	154<br>89.0%	19<br>11.0%	173	154.4<br>92.1%	13.3<br>7.9%	167.7
June	37<br>38.1%	60<br>61.9%	97	29.3<br>27.4%	77.7<br>72.6%	107.0
Total	791<br>70.8%	327<br>29.2%	1,118	759.9<br>68.0%	358.4<br>32.0%	1,118.3<br>100.0%

Row results are N (Top) and % (Bottom) in each cell.

For peer review only

Table S3. Clinical presentations of pediatric CV19 positive deaths

Table S3a. Clinical presentation of COVID-19+ facility deaths in children aged 0-19 years

No.	Age in years	Respiratory Symptoms	Gastrointestinal Symptoms	Fever	Cause of Death*
1	<1	difficulty breathing	diarrhea	X	sepsis
2	<1	cough		X	pneumonia
3	<1	cough, difficulty breathing, shortness of breath	diarrhea, vomiting	X	pneumonia
4	<1	difficulty breathing			sepsis
5	<1	cough, difficulty breathing		X	COVID-19 pneumonia
6	<1	difficulty breathing			sepsis
7	<1	difficulty breathing			pneumonia
8	<1	difficulty breathing			sepsis
9	<1	runny nose			sepsis
10	<1	difficulty breathing			sepsis
11	<1	cough	vomiting		sepsis
12	<1	difficulty breathing		X	sepsis
13	<1		vomiting	X	sepsis
14	<1			X	sepsis
15	<1			X	sepsis
16	<1				sepsis
17	<1	difficulty breathing, shortness of breath		X	sepsis
18	<1			X	sepsis
19	<1			X	sepsis
20	<1	cough, difficulty breathing		X	sepsis
21	<1	difficulty breathing			sepsis
22	<1				hypothermia
23	<1			X	sepsis
24	<1	difficulty breathing			pneumonia
25	<1	cough, difficulty breathing		X	pneumonia
26	1-5	shortness of breath		X	sepsis
27	1-5		diarrhea, vomiting		diarrhea
28	1-5				dehydration
29	1-5		diarrhea	X	malnutrition
30	1-5		diarrhea	X	sepsis
31	1-5			X	respiratory failure
32	6-10				diarrhea
33	6-10	difficulty breathing			sepsis
34	11-15				intracranial abscess

* Cause of death from the official death certificate completed by the UTH medical examiner.

For peer review only

Table S3b. Clinical presentation of COVID-19+ community deaths in children aged 0-19 years

No.	Age in years	Respiratory Symptoms	Gastrointestinal Symptoms	Fever
1	<1	difficulty breathing		
2	<1	cough		X
3	<1	cough, difficulty breathing	vomiting	
4	<1		vomiting	
5	<1			X
6	<1		diarrhea, vomiting	X
7	<1	cough, difficulty breathing, fast breathing, shortness of breath		X
8	<1			
9	<1			
10	<1	cough, fast breathing		X
11	<1		diarrhea, vomiting	X
12	<1	cough, difficulty breathing	diarrhea	X
13	1-5	cough		X
14	1-5		diarrhea, vomiting	
15	1-5		diarrhea, vomiting	X
16	1-5		diarrhea	
17	1-5		diarrhea, vomiting	X
18	1-5			
19	1-5			X
20	1-5		diarrhea	
21	1-5	cough	diarrhea	
22	1-5			
23	1-5	cough, difficulty breathing		
24	6-10			
25	6-10		diarrhea	
26	16-19	difficulty breathing		
27	16-19	cough, difficulty breathing		
28	16-19			X
29	16-19			
30	16-19			

We do not include a cause of death for the community deaths. A death certificate is still issued for each case, but these are not based on information from medical providers and are not considered to be accurate.

For peer review only

Supplementary Table S4. The relationship between age and PCR results and viral load as measured through cycle threshold values (Ct).

Age at death (years)	Ct≥40 (Neg)	Weaker (30≤Ct<40)	Strong-est (Ct<30)	Ratio of Weaker to Strongest	Ct≥40 (Neg)	Weaker (30≤Ct<40)	Strong-est (Ct<30)	Ratio of Weaker to Strongest
	**Unweighted**				**Weighted**			
0-19	304<br>82.8	54<br>14.7	9<br>2.5	6.0	203.4<br>79.4	45.8<br>17.9	6.9<br>2.7	6.6
20-39	158<br>66.7	55<br>23.2	24<br>10.1	2.3	184.2<br>67.2	62.3<br>22.7	27.7<br>10.1	2.3
40-59	171<br>74.0	36<br>15.6	24<br>10.4	1.7	190.1<br>75.2	39.4<br>15.6	23.4<br>9.3	1.7
60-79	107<br>58.5	45<br>24.6	31<br>16.9	1.4	119.8<br>56.1	54.8<br>25.7	38.9<br>18.2	1.4
80-99	42<br>52.5	19<br>23.8	19<br>23.8	1.0	55.4<br>52.0	26.1<br>24.5	25.0<br>23.5	1.0
100+	0<br>0.0	1<br>50.0	1<br>50.0	1.0	0<br>0.0	1.6<br>50.0	1.6<br>50.0	1.0
Total	782	210	108	1.9	752.9	230.0	123.5	1.9

*4 had missing age data and 12 had no Ct value (tested antemortem with rapid test).

Row results are N (Top) and % (Bottom) in each cell.

Within each age strata we plot the proportion of decedents with a 'negative' test, defined as Ct >40, or a positive test, stratified by Ct strata. Ct is the inverse of the viral load, hence low Ct equals a stronger PCR result and a higher vial load. What we observe is an age dependent effect on the ratio of weaker to strongest PCR strata. Among younger individuals ≤19 years, the ratio of weaker to strongest PCR results was 6.6:1, after weighting. This ratio declines as a function of increasing age. If we assume that viral load is reflective of pathology, then the higher ratios seen in the youngest group indicate a greater proportion of cases where COVID-19 was a co-incidental finding, whereas those in the older age groups, indicate that COVID-19 was more likely to have been a causal factor in those deaths.

For peer review only

Figure S1. Comparison of death by age distributions for the total deceased cohort, the enrolled cohort, and the CV19 positive enrolled cohort.

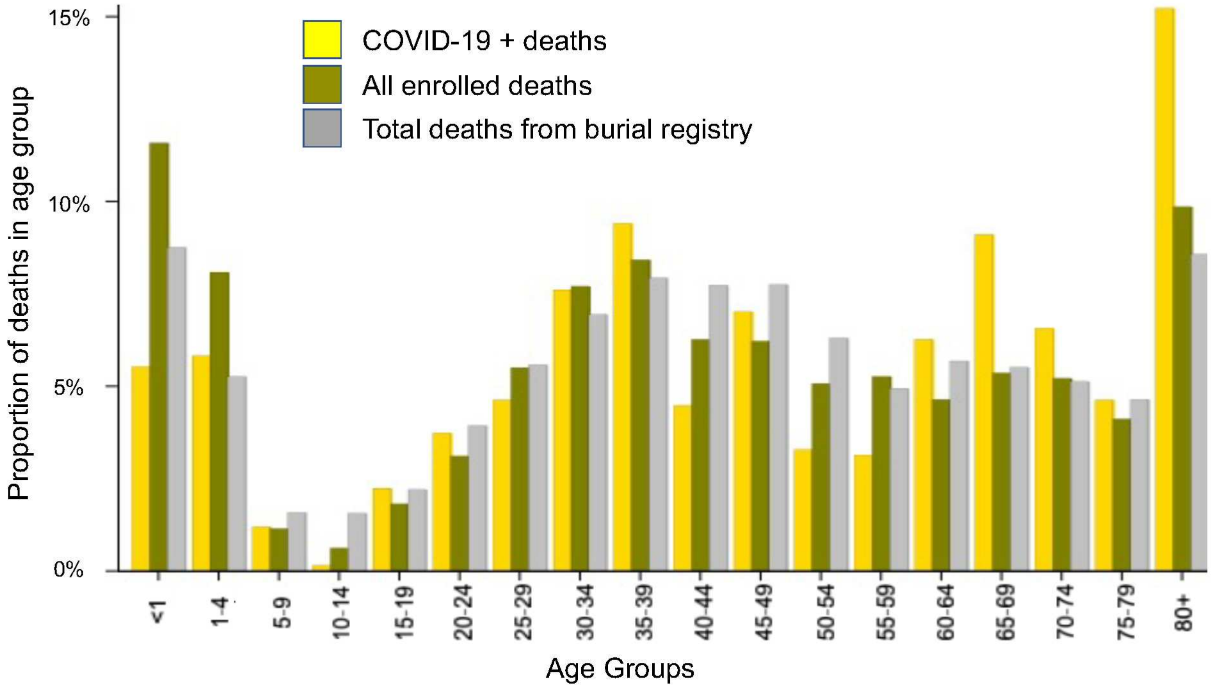

Supplementary Figure S1. Comparison of death by age distributions for the total deceased cohort, the enrolled cohort, and the COVID-19 positive enrolled cohort.

Data for the total death cohort were from Lusaka's burial registry logs maintained by the Zambian Ministry of Health. From each entry, we extracted data of death, age, and sex. We then extracted those deaths that corresponded to the periods of surveillance to capture the age by death distribution for the total cohort (enrolled and unenrolled). The second two sets correspond to the total enrolled cohort (N=1,118 person) and the subset who were COVID-19+ (N=327).

Figure S2. Distribution of cycle threshold values for PCR results targeting the N1 and N2 nucleocapsid proteins.

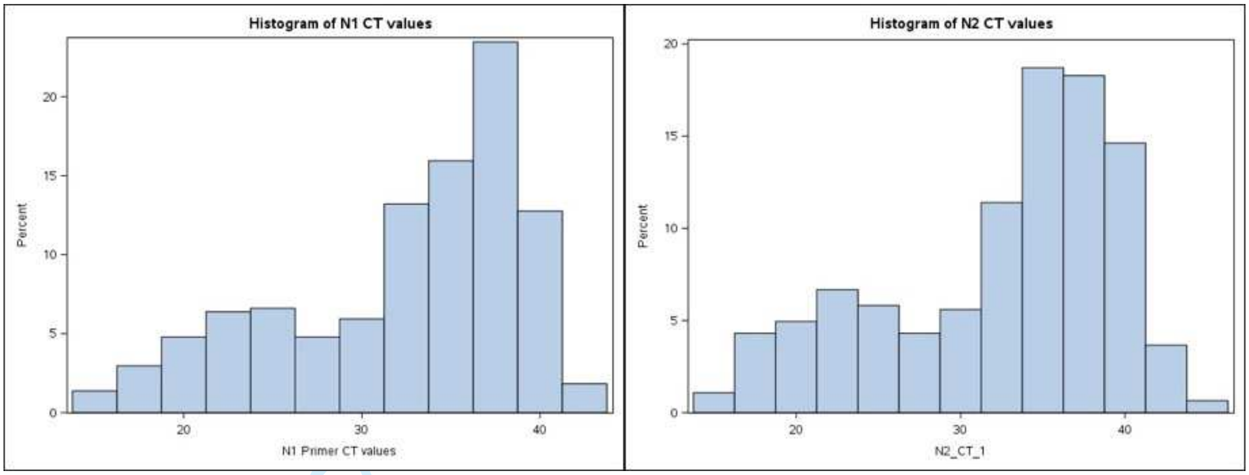

Supplementary Figure S2. Distribution of cycle threshold values for the N1 and N2 PCR reactions

The median Ct results were 32 and 33, respectively for the N1 and N2 targets. Results for detections between >40 and 45 are noted in text but are not included in any of our summary results otherwise.

For peer review only

Supplementary Figure S3

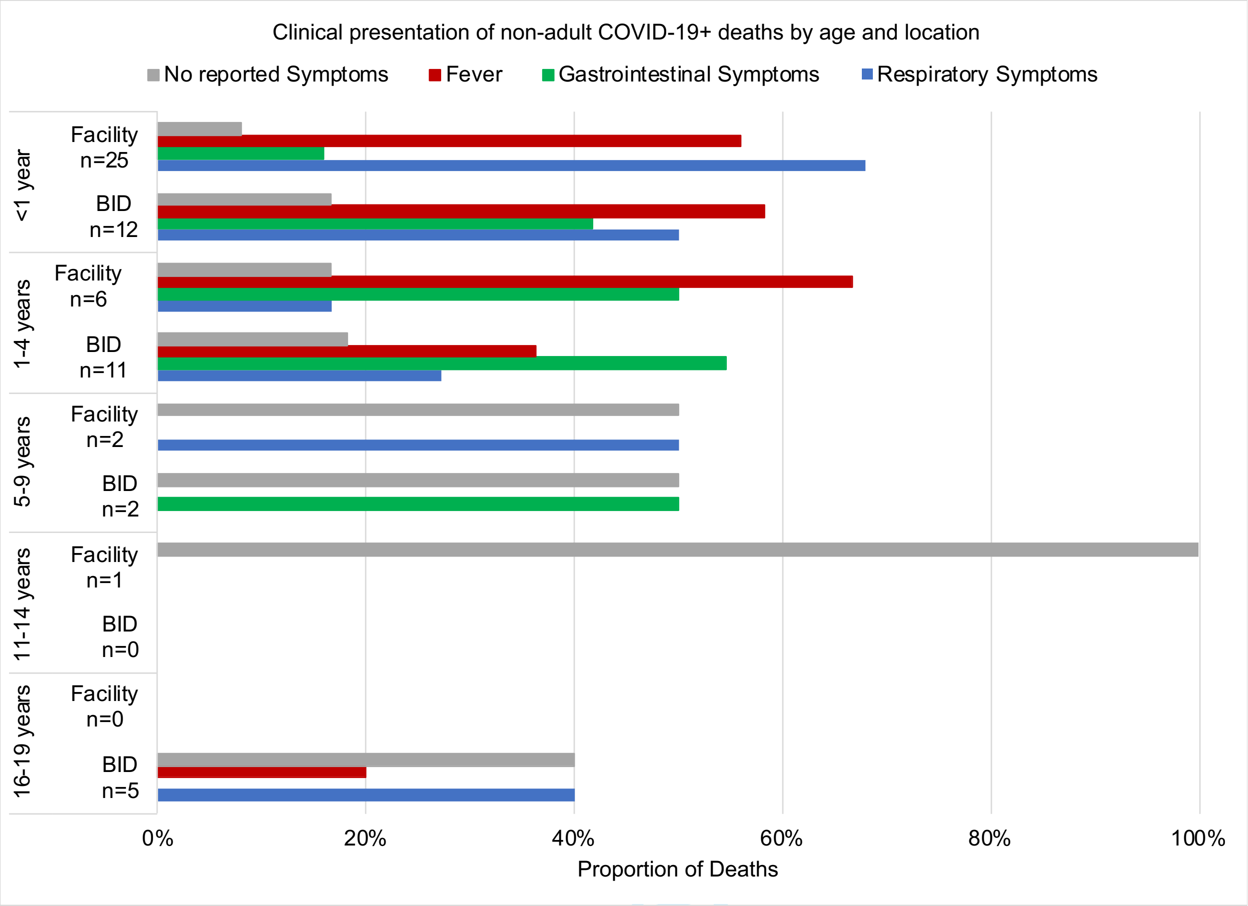

Supplementary Figure S3. Clinical presentations among COVID-19 positive children ≤19 years

For this analysis, we extracted the data about clinical presentation of each case from the medical charts (facility deaths) or verbal autopsy data (community deaths). We then clustered these syndromically into those that were 'respiratory' vs. 'gastrointestinal' vs. 'other'. Since 'fever' could occur in any of these, or by itself, we summarized this separately. As can be seen, the syndromic presentation of COVID-19+ infants <1 is distinct from that in older children. In the infants, we see a high proportion with gastrointestinal complaints and a relative paucity with respiratory disease. In the older children, this pattern reverses, and in adolescents the pattern is consistent with that seen in adults, i.e., various combinations of respiratory symptoms, often accompanied by fever. Since this is an analysis by individual, there was no need to make weighting adjustments to account for enrollment ratios.

STROBE Statement—Checklist of items that should be included in reports of ***cross-sectional studies***

	Item No	Recommendation
**Title and abstract**	1	(*a*) Indicate the study's design with a commonly used term in the title or the abstract***Done pg 1
		(*b*) Provide in the abstract an informative and balanced summary of what was done and what was found***Done
**Introduction**		
Background/rationale	2	Explain the scientific background and rationale for the investigation being reported*** Done
Objectives	3	State specific objectives, including any prespecified hypotheses*** Done
**Methods**		
Study design	4	Present key elements of study design early in the paper*** Done
Setting	5	Describe the setting, locations, and relevant dates, including periods of recruitment, exposure, follow-up, and data collection*** Done
Participants	6	(*a*) Give the eligibility criteria, and the sources and methods of selection of participants*** Done
Variables	7	Clearly define all outcomes, exposures, predictors, potential confounders, and effect modifiers. Give diagnostic criteria, if applicable*** Done
Data sources/ measurement	8*	For each variable of interest, give sources of data and details of methods of assessment (measurement). Describe comparability of assessment methods if there is more than one group*** Done pg 3
Bias	9	Describe any efforts to address potential sources of bias*** Done pg 5
Study size	10	Explain how the study size was arrived at***Done pg 7
Quantitative variables	11	Explain how quantitative variables were handled in the analyses. If applicable, describe which groupings were chosen and why*** not applicable – we did not do modeling
Statistical methods	12	(*a*) Describe all statistical methods, including those used to control for confounding*** Not applicable to this study design
		(*b*) Describe any methods used to examine subgroups and interactions*** Not applicable, there was no modelling in this analysis
		(*c*) Explain how missing data were addressed*** We did not use imputation so this did not apply.
		(*d*) If applicable, describe analytical methods taking account of sampling strategy*** Not applicable
		(*e*) Describe any sensitivity analyses*** Not applicable
**Results**		
Participants	13*	(a) Report numbers of individuals at each stage of study—eg numbers potentially eligible, examined for eligibility, confirmed eligible, included in the study, completing follow-up, and analysed*** Not applicable -this was a cross sectional study, so attrition does not apply
		(b) Give reasons for non-participation at each stage*** Not applicable
		(c) Consider use of a flow diagram*** Also necessary. This was a prevalence study conducted among postmortem participants. There is no 'flow' to report under this methodology
Descriptive data	14*	(a) Give characteristics of study participants (eg demographic, clinical, social) and information on exposures and potential confounders*** Done pg10

For peer review only - http://bmjopen.bmj.com/site/about/guidelines.xhtml

		(b) Indicate number of participants with missing data for each variable of interest*** Not applicable
Outcome data	15*	Report numbers of outcome events or summary measures*** Done pg 9 and actually throughout the results
Main results	16	(*a*) Give unadjusted estimates and, if applicable, confounder-adjusted estimates and their precision (eg, 95% confidence interval). Make clear which confounders were adjusted for and why they were included*** Not applicable. We did not do modelling.
		(*b*) Report category boundaries when continuous variables were categorized***Not applicable
		(*c*) If relevant, consider translating estimates of relative risk into absolute risk for a meaningful time period*** Not applicable. We do not report on relative risks.
Other analyses	17	Report other analyses done—eg analyses of subgroups and interactions, and sensitivity analyses*** not applicable
**Discussion**		
Key results	18	Summarise key results with reference to study objectives*** Done pg 16
Limitations	19	Discuss limitations of the study, taking into account sources of potential bias or imprecision. Discuss both direction and magnitude of any potential bias*** Done pgs 17-18
Interpretation	20	Give a cautious overall interpretation of results considering objectives, limitations, multiplicity of analyses, results from similar studies, and other relevant evidence*** Done. pg 18
Generalisability	21	Discuss the generalisability (external validity) of the study results*** Done pg 18
**Other information**		
Funding	22	Give the source of funding and the role of the funders for the present study and, if applicable, for the original study on which the present article is based*** Done pg 20

*Give information separately for exposed and unexposed groups.

Note: An Explanation and Elaboration article discusses each checklist item and gives methodological background and published examples of transparent reporting. The STROBE checklist is best used in conjunction with this article (freely available on the Web sites of PLoS Medicine at http://www.plosmedicine.org/, Annals of Internal Medicine at http://www.annals.org/, and Epidemiology at http://www.epidem.com/). Information on the STROBE Initiative is available at www.strobe-statement.org.

For peer review only

BMJ Open

What is the prevalence of COVID-19 detection by PCR among deceased individuals in Lusaka, Zambia? A post-mortem surveillance study

Journal:	*BMJ Open*
Manuscript ID	bmjopen-2022-066763.R2
Article Type:	Original research
Date Submitted by the Author:	11-Nov-2022
Complete List of Authors:	Gill, Christopher; Boston University School of Public Health, Department of International Health<br>Mwananyanda, Lawrence; Boston University School of Public Health, Research<br>MacLeod, William; Boston University School of Public Health, Global Health<br>Kwenda, Geoffrey; University of Zambia - Ridgeway Campus, Biomedical Sciences<br>Pieciak, Rachel; Boston University School of Public Health, Global Health<br>Etter, Lauren; Boston University School of Public Health, Department of Global Health<br>Bridges, Daniel; Program For Applied Technology In Health (PATH)<br>Chikoti, Chilufya; Avencion Limited<br>Chirwa, Sarah; Avencion Limited<br>Chimoga, Charles; Avencion Limited<br>Forman, Leah; Boston University School of Public Health, Department of Global Health; Boston University School of Public Health<br>Katowa, Ben; Macha Research Trust<br>Lapidot, Rotem; Boston Medical Center, pediatric infectious diseases<br>Lungu, James; Avencion Limited<br>Matoba, Japhet; Macha Research Trust<br>Mwinga, Gift; Avencion Limited<br>Mubemba, Benjamin; The Copperbelt University, Wildlife Sciences<br>Mupila, Zachariah; Avencion<br>Muleya, Walter; University of Zambia School of Veterinary Medicine, Biomedical Sciences<br>Mwenda, Mulenga; Program for Applied Technology in Health<br>Ngoma, Benard; Avencion Limited<br>Nakazwe, Ruth; University of Zambia University Teaching Hospital, Biomedical sciences<br>Nzara, Diana; Avencion Limited<br>Pawlak, Natalie; Tufts University School of Medicine<br>Pemba, Lillian; Avencion Limited<br>Saasa, Ngonda; University of Zambia School of Veterinary Medicine<br>Simulundu, Edgar; Macha Research Trust<br>Yankonde, Baron; Avencion Limited<br>Thea, Donald; Boston University School of Public Health, Global Health
Primary Subject	Global health

Heading:	
Secondary Subject Heading:	Infectious diseases
Keywords:	COVID-19, Epidemiology < INFECTIOUS DISEASES, PUBLIC HEALTH

SCHOLARONE™
Manuscripts

I, the Submitting Author has the right to grant and does grant on behalf of all authors of the Work (as defined in the below author licence), an exclusive licence and/or a non-exclusive licence for contributions from authors who are: i) UK Crown employees; ii) where BMJ has agreed a CC-BY licence shall apply, and/or iii) in accordance with the terms applicable for US Federal Government officers or employees acting as part of their official duties; on a worldwide, perpetual, irrevocable, royalty-free basis to BMJ Publishing Group Ltd ("BMJ") its licensees and where the relevant Journal is co-owned by BMJ to the co-owners of the Journal, to publish the Work in this journal and any other BMJ products and to exploit all rights, as set out in our licence.

The Submitting Author accepts and understands that any supply made under these terms is made by BMJ to the Submitting Author unless you are acting as an employee on behalf of your employer or a postgraduate student of an affiliated institution which is paying any applicable article publishing charge ("APC") for Open Access articles. Where the Submitting Author wishes to make the Work available on an Open Access basis (and intends to pay the relevant APC), the terms of reuse of such Open Access shall be governed by a Creative Commons licence – details of these licences and which Creative Commons licence will apply to this Work are set out in our licence referred to above.

Other than as permitted in any relevant BMJ Author's Self Archiving Policies, I confirm this Work has not been accepted for publication elsewhere, is not being considered for publication elsewhere and does not duplicate material already published. I confirm all authors consent to publication of this Work and authorise the granting of this licence.

For peer review only

TITLE

What is the prevalence of COVID-19 detection by PCR among deceased individuals in Lusaka, Zambia? A post-mortem surveillance study

AUTHORS

Christopher J Gill [1], Lawrence Mwananyanda [1,2], William MacLeod [1], Geoffrey Kwenda [3], Rachel Pieciak [1], Lauren Etter [1], Daniel Bridges [4], Chilufya Chikoti [2], Sarah Chirwa [2], Charles Chimoga [2], Leah Forman [5], Ben Katowa [6], Rotem Lapidot [1], James Lungu [2], Japhet Matoba [6], Gift Mwinga [2], Benjamin Mubemba [7], Zachariah Mupila [2], Walter Muleya [8], Mulenga Mwenda [8], Benard Ngoma [2], Ruth Nakazwe [2, 9], Diana Nzara [2], Natalie Pawlak [10], Lillian Pemba [2], Ngonda Saasa [8], Edgar Simulundu [6], Baron Yankonde [2], and Donald M. Thea [1].

Corresponding Author: Christopher J. Gill, MD MS, Professor, Dept. of Global Health, Boston University School of Public Health, cgill@bu.edu

AFFILIATIONS

1. Boston University School of Public Health, Department of Global Health, Boston, Massachusetts, USA
2. Avencion Limited, Lusaka, Zambia
3. University of Zambia, School of Health Sciences, Department of Biomedical Sciences, Lusaka, Zambia
4. Program for Applied Technology in Health (PATH), Malaria Control and Elimination Partnership for Africa, Lusaka, Zambia
5. Biostatistics and Epidemiology Data Analytics Center, Boston University School of Public Health, Boston, Massachusetts, USA
6. Macha Research Trust, Choma, Zambia
7. Copperbelt University, School of Natural Resources, Department of Wildlife Sciences, Kitwe, Zambia
8. University of Zambia, School of Veterinary Medicine, Department of Biomedical Sciences, Lusaka, Zambia
9. University Teaching Hospital, Department of Microbiology, Lusaka, Zambia
10. Tufts University school of Medicine, Boston, Massachusetts, USA

For peer review only

ABSTRACT

Objectives. To determine the prevalence of COVID-19 post-mortem setting in Lusaka, Zambia.
Design. A systematic, post-mortem prevalence study.
Setting. A busy, inner-city morgue in Lusaka.
Participants. We sampled a random subset of all decedents who transited the UTH morgue. We sampled the posterior nasopharynx of decedents using quantitative PCR. Prevalence was weighted to account for age-specific enrollment strategies.
Interventions. Not applicable – this was an observational study.
Primary outcomes. Prevalence of COVID-19 detections by PCR. Results were stratified by setting (facility vs. community deaths), age, demographics, and geography, and time.
Secondary outcomes. Shifts in viral variants; causal inferences based on Ct values and other features; antemortem testing rates.
Results. From 1,118 decedents enrolled between Jan-June 2021, COVID-19 was detected among 32.0% (358/1,116). Roughly four COVID-19+ community deaths occurred for every facility death. Antemortem testing occurred for 52.6% (302/574) of facility deaths but only 1.8% (10/544) of community deaths and overall, only ~10% of COVID-19+ deaths were identified in life. During peak transmission periods, COVID-19 was detected in ~90% of all deaths. We observed three waves of transmission that peaked in July 2020, January 2021, and ~June 2021: the AE.1 lineage and the Beta and Delta variants, respectively. PCR signals were strongest among those whose deaths were deemed ‘probably due to COVID-19’, and weakest among children, with an age-dependent increase in PCR signal intensity.
Conclusions. COVID-19 was common among deceased individuals in Lusaka. Antemortem testing was rarely done, and almost never for community deaths. Suspicion that COVID-19 was the cause of deaths was highest for those with a respiratory syndrome and lowest for individuals <19 years.

For peer review only

Strengths

- Decedents were enrolled based on an *a priori* sampling scheme to minimize selection biases
- Contemporaneous death by age strata data for the whole deceased population were obtained to infer whether our sample was representative of the larger whole
- Where possible, samples underwent genetic sequencing, to overlay shifts in the prevalence of viral variants (Beta and Delta) with the prevalence of COVID19 in the morgue

Limitations

- Establishing causality based on sampling of the nasopharynx has limitations
- Our data all come from one city in one country, so we cannot directly generalize our results to other locations in Africa.

For peer review only

INTRODUCTION

Despite the evident harm from the COVID-19 pandemic globally, accurate data about the pandemic's impact in Africa remain sparse. A recent multi-site data identified the severe impact of COVID-19 in terms of deaths in African intensive care units. However, the ICU population is not broadly generalizable, and such observations do not yield a direct estimate of the overall prevalence of COVID-19 or its impact at a population level.[1] In early 2021, our team reported results from systematic COVID-19 post-mortem surveillance conducted from June to October 2020 at the central morgue in Lusaka, Zambia.[2] Using polymerase chain reaction (PCR), we detected COVID-19 in nearly 20% of decedents, most of whom had died in the community. In that group, none had been tested for COVID-19 prior to death. Yet, even among facility deaths, antemortem testing was uncommon. Hence, COVID-19+ deaths appeared rare in Lusaka because testing was rarely done in life. We theorized that the so-called 'Africa Paradox'[3-8] was a myth born from insufficient surveillance data. Other groups have since reached similar conclusions.[9-12]

Our analysis yielded other provocative findings. In contrast with the US and other high-income country (HIC) settings where deaths are concentrated in the elderly, COVID-19+ deaths in Lusaka occurred more evenly across the age spectrum. Further, 10% of COVID-19+ deaths occurred in children, a surprising finding given how rare pediatric COVID-19 deaths are in HICs. It remains an open question whether these pediatric deaths were caused by COVID-19 or whether the virus was a coincidental finding.

These conclusions were conservative given that our analysis rested on only seventy COVID-19+ decedents. Subsequently, our team conducted a second round of post-mortem COVID-19 surveillance, spanning the period January through June 2021. In this longer and larger study, we again quantified the burden of fatal COVID-19 by setting (community vs. facility deaths), calendar date, and geography; and observed the impact and patterns of clinical presentation among children vs. adults. In addition, we aligned our prevalence data with genetic sequencing to characterize shifts in viral lineages/variants over time.

METHODS

Overview

Our COVID-19 surveillance work builds upon a larger body of postmortem surveillance work initiated to define the fatal burden of Respiratory Syncytial Virus and *Bordetella pertussis*. With the onset of the pandemic, the presence of this established infrastructure and team allowed us to quickly amend our protocols to shift from an exclusive focus on RSV and pertussis in infants to measure COVID-19 prevalence across all ages. The RSV results have been published,[13-15] and the pertussis results are currently under review.

We direct the readers to our prior publication for complete details of the surveillance methods.[2] Throughout this paper, we refer to several rounds of surveillance. Round 1 ran from June-Oct 2020; Round 2, comprising the new data in this paper, ran from Jan-June 2021. Round 3 is ongoing, was initiated early in 2022, and is planned to run

For peer review only - http://bmjopen.bmj.com/site/about/guidelines.xhtml

through January 2023. These surveillance windows were the solely due to funding and not based on assumptions about COVID-19 prevalence over time.

The research was conducted with the approval of the ethical review boards from Boston University (reference number H-36469) and the University of Zambia (reference number 015-11-18); written informed consent was obtained from the next of kin or family representative in all cases.

Sampling strategy

When capturing a high proportion of all deaths, postmortem surveillance is robust against ascertainment biases and provides a direct and unambiguous measure of a disease's prevalence, though inferring causality due to the disease may still be challenging. Given concerns about viral RNA degradation leading to false negative results, enrollment was restricted to deaths within the preceding 48 hours.

We enrolled deceased individuals at the University Teaching Hospital (UTH) morgue. UTH is the main teaching hospital for the University of Zambia Medical School. Its morgue is the largest in the city and where ~80% of decedents are passed on to their families, irrespective of socio-economic status of the decedent. This, plus the legal requirement to obtain a burial certificate before interring a body, most of which are issued at the UTH morgue, makes it highly representative of all deaths in Lusaka.

Due to the high volume in the morgue and finite capacity of our team, we capped enrollments at ~5-6 deaths per day. Also due to high volume, we enrolled community deaths at a 1:3 ratio, while enrolling the less common facility deaths at a 1:1 ratio. In response to the surprising high proportion of pediatric deaths in surveillance Round 1, we elected to also oversample this group enrolling infants (<1 year) at a 1:1 ratio. This required that we adjust for these ratios in our total population prevalence estimates (see statistical analysis). Our team had no access to the clinical data about each decedent and could not know the PCR results prior to consent and enrollment. Therefore, we were confident that our enrolment strategy was robust against selection biases with respect to COVID-19 status.

Concurrently, we extracted total deaths by age stratum from the official government burial registry. This allowed us to estimate the contemporaneous proportion of all deaths represented by our sample, and, by comparing the age by death distribution of the two groups, assess whether the enrolled sample was representative of the total.

Data collection and case definitions

We defined a 'facility death' as one that occurred under care at UTH or a referring facility. We defined a community death as one that occurred outside of medical care. We defined pediatric deaths as those occurring between 0-≤19 years, recognizing that some may choose to define the end of adolescence differently, and that such definitions may not align with official age of emancipation laws in different parts of the world.

Following enrollment, individual and household demographic data were collected on all decedents, along with any antemortem COVID-19 testing results that were documented in the medical chart (for facility deaths) or reported by the next of kin/representative of the decedent during the verbal autopsy (for community deaths).

For peer review only - http://bmjopen.bmj.com/site/about/guidelines.xhtml

The most difficult challenge was inferring the causal role of COVID-19 in each death: the SARS-CoV-2 virus may have a direct or indirect role in deaths or could be a coincidental finding. The US CDC has issued guidance stating that in deaths where COVID-19 is detected, the virus should be assumed to be the direct or underlying cause of death, absent exonerating circumstances.[16] That notwithstanding, to better infer the causal role of COVID-19 in deaths, we used the clinical data describing symptoms during the fatal event to categorize the syndromic presentation of each death. For facility deaths, these data were obtained from a medical chart extraction focusing on symptoms at initial presentation to UTH; for community deaths, we conducted a verbal autopsy using the abbreviated tool validated by the Institute for Health Metrics Evaluation (IHME).[17] From these syndromic data, using the same approach as in our initial publication,[2] we classified COVID-19 PCR+ adult decedents (>19 years) into the following categories:

1) Probable COVID-19 deaths, where the individual had presented with any combination of fever and/or respiratory symptoms.
2) Possible COVID-19 deaths, where the individual presented without classic respiratory symptoms, but rather with known sequalae of COVID-19 suggestive of a heart attack, stroke, or other acute vascular event related to COVID-19's propensity to increase the risk of clotting disorders.[18]
3) Probably not COVID-19 deaths, where the individual had an exonerating cause of death suggesting that the COVID-19 finding on PCR was co-incidental.
4) Unknown, where the data were insufficient to adjudicate.

The reasons we only applied this strategy to adults is that this taxonomy quickly proved ill-suited for younger individuals. Unlike adults, where fever and/or respiratory symptoms predominated, the pediatric presentations were more variable and respiratory symptoms less common. Since we were (and remain) uncertain about how COVID-19 should present clinically in children, we worried that creating *a priori* case definitions based on how the virus behaves in adults could be misleading. Similarly, applying a new definition developed iteratively from the constellation of symptoms observed in children and then applying that definition forwards without validation against an external gold standard (such as histopathology coupled with PCR), constitutes a logical circularity.

Facing this uncertainty, we opted simply to describe the pediatric case presentations without attempting to infer causality for decedents aged ≤19 years.

Biological sample collection

From each deceased individual, we obtained posterior nasopharyngeal (NP) samples. We used flocked-tipped NP swabs (Copan Diagnostics, Murietta, CA, USA),[19 20] inserting the swab into both nares and then rotating 180 degrees in both directions and collected into 3mL vials of universal transport media. Samples were stored cold or on ice until processing at our onsite molecular lab.

For peer review only - http://bmjopen.bmj.com/site/about/guidelines.xhtml

Laboratory procedures

PCR testing for COVID-19

After vortexing to elute samples, total nucleic acid for PCR was extracted using the EasyMAG system (Biomerieux, Marcy L'Etoile, France).[21] We ran PCR for 45 cycles using the US CDC's COVID-19 testing kit, which targets the N1 and N2 nucleocapsid proteins.[22] We also ran PCR on each sample against the constitutive human enzyme RNAseP. The presence of RNAseP indicates that the NP swab made effective contact with the respiratory mucosa and that there were no PCR inhibitors.

Per the instructions in the CDC's protocol, we defined a COVID-19 test result as positive if the PCR cycle threshold (Ct) was <40 for both the N1 and N2 genes, provided that each assay demonstrated a logarithmic fluorescence amplification curve, had RNAseP detectable at Ct<40, and had positive and negative plate controls performing as anticipated.

However, Ct is a continuous variable, and the selection of any Ct cut point is arbitrary and hence controversial.[23] While a lower Ct, reflecting a higher viral load, is often associated with increased clinical severity, most COVID-19 surveillance studies assess individuals when they present acutely. In the acute setting, viral loads, and hence PCR signals, are likely at peak intensity. By contrast, the population in this study were all tested after they had died, and currently there are few data about what PCR signals should look like at the end of an arc of infection. At that point, the viral load and PCR signal intensity could have declined (or be undetectable), even if COVID-19 had set in motion a set of events culminating in death. For this reason, we accepted a Ct <40 as positive, even though lower Ct values have often been used for defining COVID-19 status at the time of initial presentation. But since this substitutes one arbitrary cut point (<40) for another arbitrary cut point (<30), we also report the number of decedents with COVID-19 detectable between Ct >40 to ≤ 45, though we have not included these in our summary analyses.

Genetic sequencing of selected samples

To characterize the distributions of novel COVID-19 variants, we conducted genetic sequencing to detect viral variants on the subset of samples with a Ct <30. Prior experience has shown that nucleic acid concentrations are rarely sufficient for sequencing above this threshold. For this, we tested samples both from surveillance Rounds 1 and 2, describing shifts in dominant COVID-19 variants in Lusaka over the cumulative period of surveillance.

Total RNA was re-extracted from vortexed NP swab specimens using the QIAamp® Viral RNA Mini Kit (Qiagen, Hilden, Germany) as prescribed by the manufacturer. Complementary DNA was synthesized using LunaScript® RT SuperMix Kit (New England Biolabs, Ipswich, Massachusetts, USA) according to the manufacturer's protocol and multiplex PCR was conducted using custom primers, which were designed using Geneious software version 10.0.9, as used previously to sequence the first COVID-19 case in Zambia using the Sanger method.[24 25] The multiplex PCR generated overlapping amplicons which were then sequenced on the MinION (Oxford Nanopore Technology, United Kingdom) platform. The samples were multiplexed using the Oxford

For peer review only - http://bmjopen.bmj.com/site/about/guidelines.xhtml

Nanopore native barcoding expansion kits 1–12 and 13–24 in combination with the ligation sequencing kit 109 (Oxford Nanopore). The data generated through the MinION was processed using the standard ARTIC bioinformatic pipeline (https://artic.network/ncov-2019/ncov2019-bioinformatics-sop.html). After removal of sequencing primers, the consensus sequences were deposited in the GISAID database (https://www.gisaid.org/). PANGO lineages were then determined for each genome using Pangolin v3.1.16 (https://pangolin.cog-uk.io/).

Statistical analysis

Given the uncertainties of future detection rates for COVID-19, our sampling was not based on *a priori* power assumptions. Rather, we sought to enroll as many decedents as our team could reasonably accommodate. Our statistical analysis was a straightforward comparison of proportions that did not require statistical modeling or imputation. COVID-19 status, based on PCR, was stratified by setting (community vs. facility deaths), by age groups, by calendar date, and by geography (city ward).

Where indicated, we have adjusted prevalence estimates by calculating weighted outputs to account for the different sampling ratios used during enrolment. We calculated weights so that the sum of the weighted population would sum to the sampled population: unweighted sample (1,118) = weighted sample (1,118). This allowed us to calculate the relative frequency of our unweighted sample in the four weighted groups: (1) facility deaths < 1 year, (2) all other facility deaths, (3) community deaths < 1 year of age, and (4) all other community deaths. We sampled the facility deaths < 1 year, all other facility deaths, and community deaths in infants < 1 year at a 1:1 ratio, and the community deaths ≥ 1 year at a 1:3 ratio. We then calculated the weighted sample by multiplying the inverse of the sampling ratio and calculated the relative frequency of the weighted sample. Setting the weighted sample size at the actual enrolled sample size of 1,118, yielded sample weights of 0.53 for the portion of the sample that we recruited as 1:1 and the sample weight of 1.60 for the portion of the sample that we recruited as 1:3. Hence, the ratio of these sample weights, adjusting for rounding, is 1.60/0.53 =~3:1. The reason for this weighting should be self-evident, since without such weighting the prevalence estimates will necessarily over or under estimate the true prevalence across different groups of decedents.

For analyses of COVID-19 deaths over time and place and for the genetic sequence data, we combined results from Rounds 1 and 2 of surveillance. Effectively this provides a time series analysis over a 1-year period minus the 3-month gap from October 2020-Jan 2021 defined by the end and start of adjacent funding cycles. Where appropriate, we reference our results from Round 1 to compare and contextualize results from Round 2. For all statistical analyses and most data manipulations, we used SAS software (Cary, North Carolina, USA).

For the geospatial analyses, we used ArcGIS software (ESRI, Redlands, California, USA). We pulled population size data from the Zambia Data Hub: https://zambia-open-data-nsdi-mlnr.hub.arcgis.com/, which is managed by the Government of Zambia through the Ministry of Lands and Natural Resources, and the Zambia Statistics Agency. We downloaded these ArcGIS layers to the level of Lusaka's city wards. We

For peer review only - http://bmjopen.bmj.com/site/about/guidelines.xhtml

then created a 2-dimensional heat map that indexed the total number of COVID-19+ deaths against total enrolled deaths across each of Lusaka's main city wards.

Patient and Public Involvement

The participants in this study were all deceased. There was no involvement of patients or the public in the design of this study. The results of this research have already been made publicly available via a posting on MedRXiv.

RESULTS

Study overview and summary of PCR results

This second round of surveillance spanned January-June 2021. The newly enrolled cohort included 1,118 deceased individuals ranging in age from <1 to 102 years. Contemporaneously, 6,270 deaths were reported in the Lusaka burial registries. Thus, our sample represented 17.8% of all deaths in this period. The age distribution of the enrolled vs the total deceased population were similar, arguing to representativeness (**Supplementary Figure S1**).

PCR was successfully conducted on 100% of the NP swabs, and 1,116 had an RNAseP <40, indicating adequacy of sample collection. COVID-19 was detected among 29.3% (327/1,116) of decedents without weighting, and 32.0% (effective sample size = 358/1,116) after adjusting for weighting (**Table 1**).

Table 1. Postmortem COVID-19 PCR results, stratified by setting

Setting of death	Negative, CT≥40	Positive, CT<40	Totals
**Unweighted**			
**Community % (n/N)**	66% (358/543)	34% (185/543)	49% (543/1,116)
**Facility % (n/N)**	75% (431/573)	25% (142/573)	51% (573/1,116)
**Both settings combined**	71% (789/1,116	29% (327/1,116)	100% (1,116/1,116)
**Weighted**			
**Community % (n/N)**	65% 528.2	35% 282.7	72.7% 811
**Facility % (n/N)**	75% 229.5	25% 75.6	27.3% 305
**Both settings combined**	68% (757.7/1,116)	32% (358.3/1,116)	100% (1,116/1,116)

Thus, the overall prevalence of COVID-19 in Round 2 was roughly double what we observed in Round 1 (16%, 58/364). The median Ct values for the N1 and N2 targets were ~33 in both cases (**Supplementary Figure S2)**. We note that an additional 58 samples (45.8 weighted) had COVID-19 detectable at a Ct ≥40-45 but were not included in subsequent analyses.

For peer review only - http://bmjopen.bmj.com/site/about/guidelines.xhtml

The weighted proportion of females among the COVID-19+ and COVID-19- groups was nearly identical (40.6%, 453/1,116). By contrast, the COVID-19+ decedents were significantly older than those without COVID-19 (median 48 years (IQR 30-70 years) vs. 39 years (IQR 0-58 years)), Wilcoxon Ranked Sum test *p*-value < 0.001 (**Table 2**)).

Table 2. Basic demographics of the postmortem cohort, stratified by COVID-19 status

	**Negative, CT≥40**	**Positive, CT<40**	**All Deaths**
**Demographic characteristic**	(n=789)	(n=327)	(n=1,116)
**Unweighted percent Females (n/N)**	42.3% (334/789)	41.0% (134/327)	41.9% (468/1,116)
**Weighted percent female (n/N)**	41.0% 309/758	39.9% 144/358	40.6% 453/1,116
**Unweighted median age at death, years (IQR)**	32.5 (0 - 54)	44.0 (27 - 68)	36.0 (1 - 58)
**Weighted median age at death (IQR)**	39.0 (10-58)	48 (30-70)	41 (23-64)

Proportion of deaths and COVID-19+ deaths by facility vs. community setting

Within the 1,116/1,118 (99.8%) of total deaths with a valid test result, there were 573 facility deaths and 543 community deaths. While this implies that the ratio of facility to community deaths was similar, this does not account for the different enrollment ratios applied to each group. After weighting, 73% of all deaths occurred in the community vs. 27% at a facility, for a ratio of ~three community deaths for each COVID-19+ facility death.

Similarly, COVID-19 was detected among 24.8% (142/573) of facility deaths and 34.0% (185/543) of community deaths. Considering the contribution of deaths by setting, among the 327 COVID-19+ decedents, 56.6% (185/327) were community deaths and 43.4% (142/327) were facility deaths. After weighting to adjust for enrollment ratios, community deaths accounted for 79% and facility deaths for 21% of total COVID-19+ deaths. Stated another way, for each COVID-19+ decedent detected at a facility, ~four COVID-19+ decedents were identified in the community.

COVID-19 deaths by age group

During Round 1, the proportion of COVID-19+ individuals was similar across all age groups and 10% of all COVID-19+ deaths were in children ≤19 years.

Round 2 yielded a similar pattern with COVID-19+ deaths occurring across all age groups and with relatively similar proportions out of total deaths in each group (see **Figure 1**). After weighting, adults aged >19 years comprised 86.2% (300/358) of COVID-19+ deaths and children 0 to ≤19 years for 14.9% (53/358) (**Table 3**).

For peer review only - http://bmjopen.bmj.com/site/about/guidelines.xhtml

Table 3. Distribution of COVID-19 positive deaths by 20-year increments

Age at death (years)	Ct>=40 (Neg)	Ct<40 (Pos)	Total	Ct>=40 (Neg)	Ct<40 (Pos)	Total
	**Unweighted**			**Weighted**		
0-19	308<br>82.8%	64<br>17.2%	372	205.6<br>79.4%	53.3<br>20.6%	261.0
20-39	159<br>66.5%	80<br>33.5%	239	184.8<br>67.1%	90.5<br>32.9%	275.3
40-59	171<br>73.4%	62<br>26.6%	233	190.1<br>74.8%	63.9<br>25.2%	253.9
60-79	108<br>58.1%	78<br>41.9%	186	120.3<br>55.9%	94.8<br>44.1%	215.1
80-99	42<br>52.5%	38<br>47.5%	80	55.4<br>52.0%	51.1<br>48.0%	106.5
100+	0<br>0.0%	2<br>100.0%	2	0<br>0.0%	3.2<br>100.0%	3.2
Total	788*	324*	1,112*	757.7*	358.4*	1,112.1*

*4 had missing age data, of which 3 were COVID-19 positive and 1 was COVID-19 negative, for 789, 327 and 1,116, respectively
Row results are N (Top) and % (Bottom) in each cell.

Overall, 78.1% (208/358, weighted) of COVID-19+ decedents were aged <60 years. Contrasting the death by age distributions for the COVID-19+ decedents relative to the age distributions for the total population from the burial registries, the COVID-19+ deaths were relatively underrepresented among children and relatively over represented in the elderly. Outside of these age extremes, the age by death distributions for the two cohorts were similar (**Supplemental Figure S1**).

Antemortem testing for COVID-19

During Round 1, antemortem testing for COVID-19 was rarely done, occurring in only 10% of facility deaths and none of the majority of deaths that were in the community.

In Round 2, antemortem testing rates improved but only among facility deaths. Within this group, 52.6% (302/574) were tested antemortem, of whom 25 were reportedly positive (see **Supplementary Table S1**). During this period, nearly all COVID-19 testing at UTH relied on rapid antigen tests. However, among the community deaths, we were still only able to document antemortem testing for 1.8% (10/544) of COVID-19+ decedents.

Temporal shifts in COVID-19 prevalence, dominant variants, and geography

Given these changes between Round 1 and Round 2, we further explored the relationship between total deaths, COVID-19 prevalence, viral lineages, and geographic

For peer review only - http://bmjopen.bmj.com/site/about/guidelines.xhtml

distribution in time series analyses. These results, combining Round 1 and 2 data, are presented in **Figure 2**.

Several features are noteworthy. First, the data collected in Round 1 document what in hindsight was likely the first, and smallest, of three waves of COVID-19 to occur in Lusaka during our surveillance period (**Panel A**). Noting the gap in surveillance (between adjacent funding cycles) from October 2020 to January 2021, wave 1 occurred in June-October 2020; wave 2 in January-February 2021; and wave 3 in May-June 2021. Round 2 surveillance ended in June 2021, precluding observations about when wave 3 peaked and receded.

Second, during waves 2 and 3, the proportion of decedents who tested positive for COVID-19 peaked at 91.7% prevalence in January and 83.8% in June of 2021. Both represented a statistically significant increase over the average prevalence during Rounds 1 and 2 (ANOVA, $P<0.001$). The proportion of deceased who tested positive for COVID-19 by month are summarized in **supplementary Table S2**.

Third, each wave presented a distinct pattern in terms of prevalent viral lineages (**Panel B**). Combining Rounds 1 and 2 and selecting those with a Ct<30, we successfully sequenced 96 isolates. From these, lineage AE.1 was dominant during wave 1. During wave 2 in Jan-Feb, the B.1.351 variant (the Beta variant) was dominant. Beta was supplanted by the emergence of the B1.617 variant (Delta) during wave 3, which accounted for 100% of all sequenced isolates during this period.

Concurrently, we mapped the distribution of COVID-19+ cases indexed against total deaths over time across Lusaka's major city wards (**Panel C**). The areas shaded dark blue represent areas where COVID-19+ deaths were disproportionately high relative to total deaths, while those in light pink, light blue, or white are areas where COVID-19+ deaths were underrepresented relative to total deaths. Within this taxonomy, the highest burden of COVID-19 was in Lusaka's poorest and most densely populated areas that constitute the peripheral ring of peri-urban compounds to the city's north, west, and south, and largely spared the more affluent neighborhoods that cluster in the center of the city.

Clinical presentation of COVID-19 positive children and adolescents

Recalling that infants <1 year were intentionally oversampled, requiring weighting to draw overall prevalence estimates, most deaths in children and adolescents ≤19 y were in infants. With that caveat, **Supplementary Figure S3** summarizes the syndromic presentations for the COVID-19 positive deceased children <19 years, stratifying these by age groups and community/facility settings. Since we are describing these on a per individual basis, there was no need to weight these results.

The syndromic categorizations are not mutually exclusive. Clinical presentations appeared to vary by age and setting. Among infants who died at a facility, respiratory symptoms and fever predominated, whereas fever, respiratory and gastrointestinal symptoms occurred in similar proportions among those who died in the community. Among children 1-4 years, fever and gastrointestinal symptoms were more common than respiratory symptoms. Very few COVID-19+ deaths were detected among children aged 5-14 years (N=5). Among children 16-19 years (N=5, all from the community), the

For peer review only - http://bmjopen.bmj.com/site/about/guidelines.xhtml

only reported symptoms were fever and respiratory distress, consistent with COVID-19's presentation in adults. In both community and facility settings, there were several children with no reported symptoms, though this may simply reflect a lack of documentation. Acknowledging the sparse data from older children, these results are broadly consistent with our observations in Round 1 where we had noted the relative infrequency of respiratory symptoms and commonality of gastrointestinal symptoms, particularly among children ≤ 5 years. **Supplementary Table S3** provides a detailed line listing summarizing the clinical presentation of each COVID-19+ pediatric death by setting (facility deaths **Table S3a**, community deaths **Table S3b**).

Inferring the causal role of COVID-19 in these deaths

During Round 1, among those who had sufficient clinical data to allow an assessment of causality, nearly all were judged to have 'probably' or 'possibly' died from COVID-19.

In Round 2, 89.3% (292/327) of deaths had sufficient data for clinical adjudication, leaving 10.7% (45/327) that could not be adjudicated. Excluding the pediatric cases and those with insufficient data for analysis, we were able to adjudicate 219 deceased adults. Of these, 155/219 (70.8%) were considered 'probably' or 'possibly' due to COVID-19, and 29.2% 'probably not' due to COVID-19. When adjusted for weighting, this combined proportion rose to 73.9% (181/244) (see **Table 4**).

We recognized that the categories used for adjudication (probable, possible, probably not) rested on clinical information that may have been incomplete, a particular problem with the verbal autopsy data given that the respondent providing the information was necessarily relaying these data second hand. A gold standard for asserting causality would have been PCR coupled with histology showing end organ damage consistent with SARS-2 infections. Indeed, we had previously published such pathology based on minimally invasive tissue sampling (MITS) among several of the cases here that had been diagnosed antemortem. As reported in that paper, these individuals showed evidence of diffuse organ injury to the lungs, kidneys and liver in patterns consistent with COVID-19 disease.[26] However, the identification of these decedents in life was unlikely done at random but rather based on clinical suspicion, creating the potential for ascertainment bias.

Given these limitations, we conducted additional causal inference analyses around the Ct data for the full cohort, which we felt would be more robust against selection bias. We reasoned that higher viral loads (and hence lower Ct values) should align with increased severity of disease. If so, we hypothesized that decedents deemed 'probable COVID-19', reflecting a high suspicion that COVID-19 was the cause of death, would on average have lower Ct values (reflecting higher viral loads) than those whose death was deemed 'probably not' COVID-19. The data in **Table 4** supported that hypothesis.

For peer review only

Table 4. Causal role of COVID-19 among PCR(+) deceased adults*, >19 years

Role of COVID-19 in death of patient	Median Ct (IQR)	Cases (N)	Percent	Weighted cases (N)	Weighted percent	Cumulative total for cases that could be adjudicated	Cumulative percent (n/N) for cases that could be adjudicated
Probably due to COVID-19	31.6 (24.2-35.5)	127	48.7%	142.2	46.6%	142	58.2% (142/244)
Possibly due to COVID-19	30.2 (23.2-35.7)	28	10.7%	38.3	12.6%	180	73.8% (180/244)
Probably not due to COVID-19	34.9 (28.0-37.3)	64	24.5%	63.9	20.9%	244	100% (244/244)
Insufficient data to adjudicate causality	32.5 (23.9-35.5)	42	16.1%	60.7	19.9%	Not applicable	Not applicable

* Clinical presentations of children with COVID-19 (n= 63) were variable and often lacked typical symptoms of respiratory distress. Since we were uncertain how COVID-19 should behave in children, we did not attempt to adjudicate these cases in terms of presumed causality. The results above have been weighted to adjust for the different enrollment ratios

For peer review only

6

For peer review only

For peer review only - http://bmjopen.bmj.com/site/about/guidelines.xhtml

Among those whose deaths were adjudicated as 'probably' due to COVID-19, the median Ct values were 31.6 (IQR CI 24.2-35.5) whereas those adjudicated as 'probably not' COVID-19 had a median Ct value of 34.9 (IQR 28-37.3), or a ~ten-fold increase in viral load. Thus, COVID-19+ individuals whose clinical data were most suggestive of a COVID-19 illness by presentation had the highest viral loads.

Following this reasoning further, we conducted an analysis exploring the distribution of Ct values by age groups. As noted previously, we were skeptical that COVID-19 was the cause of death in young children, largely because the clinical presentations in children, and especially children <5 years, differed from adults. Therefore, we reasoned that COVID-19 was more likely to be a coincidental finding in children. To test that contention, we stratified the proportion of PCR results by age group sorting them into two groups: those with the strongest signals (i.e., Ct<30) and those with weaker signals (i.e., Ct 30≤Ct≤40). We then calculated the ratio of the proportions of weaker to strongest Ct values within each 20-year age stratum. We hypothesized that if COVID-19 was a co-incidental finding, we would observe a higher ratio of weaker to strong results than if COVID-19 was the cause of death. The results shown in **Supplementary Table S4** supported that hypothesis. After adjusting for weighting, the ratio of weaker to stronger Ct results was 6.6:1 for those aged 0-19 years, compared with a ratio of 1.9:1 across all ages combined. With each increase in age stratum, the proportion of weak to strong PCR signals declined in a stepwise fashion, such that for those 80 years and older, the ratio was 1:1. We draw two conclusions from this analysis. First, the results supported our assumption that COVID-19 was likely a coincidental finding among children. Second, by extension, COVID-19 likely played a causal role in the deaths of older individuals.

DISCUSSION

This second round of postmortem surveillance for COVID-19 reinforces and expands upon our previous observations. First, even if we base our conclusions solely on those who were deemed 'probably due to COVID-19' based on clinical presentation, our data suggests that COVID-19 had a severe impact in Lusaka with much loss of life. Second, COVID-19+ deaths were concentrated in the community where testing for COVID-19 was absent. Third, rather than being concentrated in the elderly, COVID-19+ deaths were distributed widely across the age spectrum, and most (~80%) were in individuals aged <60 years. Fourth, COVID-19 was frequently identified among children. Among children ≤5 years, gastrointestinal complaints were common and respiratory symptoms comparatively uncommon, and we suspect that COVID-19 was more likely a co-incidental finding in these younger individuals. Lastly, the emergence of the Beta and Delta variants coincided with marked increases in the prevalence of COVID-19+ deaths, reaching ~90% at the peak of those two waves. Conservatively, COVID-19 was identified in 30% of all decedents between January and June 2021.

A consistent finding was the low proportion of decedents with COVID-19 who were diagnosed in life. We hypothesize that this can be explained as follows: 1) the majority of COVID-19+ deaths (~80%) occurred in the community where, with few exceptions, testing was unavailable; 2) among the hospital deaths, only half were tested; and 3) most of that testing done at facilities used rapid antigen tests, which are less sensitive than PCR.[27] Putting these in sequence, from the 1,118 decedents tested in our study

For peer review only - http://bmjopen.bmj.com/site/about/guidelines.xhtml

we identified COVID-19 in 327 (unweighted). Of these, only 25 had been identified antemortem at UTH, or <10% of the total based on PCR. Since these hospital data feed into the national COVID-19 surveillance data, we conclude that the prevalence COVID-19 was systematically and significantly under-represented. While it is certainly possible that individuals in the community might have self-tested for COVID using a rapid test, it is very unlikely that such results would be reported to the government of Zambia and hence fail to be captured in official government reports.

As in our prior analysis, COVID-19+ deaths were concentrated in Lusaka's most densely populated wards where Lusaka's poorest and most vulnerable citizens reside. Access to care is challenging in these communities, and we have described elsewhere how delays in seeking care may contribute to infant deaths in the community.[28] This highlights a sad element to the global COVID-19 narrative. Even within a poor country like Zambia, there is a gradient of impact that appears to fall hardest on those with the least resources.

There are several important distinctions from our findings in Round 1. First, the numbers of COVID-19 deaths and the proportion of deceased individuals who tested positive for COVID-19 both increased substantially. This coincided with the emergence of the Beta and Delta variants.

Second, the proportion of these deaths that were judged as 'probably' or 'possibly' due to COVID-19 declined from ~100% in Round 1 to ~70 % in Round 2. We acknowledge that the assessment of causality was limited by the evidence present in the medical chart (facility deaths) or reported through the verbal autopsy (community deaths). With that caveat, our procedures and tools and field team for collecting these data were the same in rounds 1 and 2, leading us to conclude that this was a real shift and not an artifact of shifting study procedures.

Our data do not provide an explanation for this decline, but several explanations are plausible. One is that earlier population exposure to COVID-19 (following the first wave) might have provided increased resistance to the SARS-CoV2 virus, i.e., the change is explained by naturally acquired herd immunity from prior exposure. COVID-19 vaccines were virtually unavailable during this period, so vaccine-derived immunity cannot explain this. A second possibility is that the Beta and Delta variants dominated the second and third waves of transmission. Both variants exhibited increased transmissibility, which plausibly accounts for the high proportions of deaths with COVID-19 at the peak of each wave. As seen with the Omicron variant, a gain in transmissibility could yield a reciprocal loss of virulence, i.e., the change is explained by virology.[29 30] However, recent epidemiologic and *in vitro* data suggest the opposite, that the Beta and particularly Delta variants were more, not less, pathogenic.[31-33] Thus, we the former explanation is more plausible.

Strengths and limitations

A strength was that our data were collected prospectively, capturing a wide spectrum of ages in community and facility settings and without prior knowledge of clinical presentation or antemortem testing results. While our capacity only allowed us to enroll a subset of all deaths at the morgue, enrollment was conducted without prior knowledge of clinical presentation or PCR status, and thus should not introduce bias affecting

For peer review only - http://bmjopen.bmj.com/site/about/guidelines.xhtml

overall prevalence estimates after adjusting for weighting. And while all came from the UTH morgue, 80% of all deaths in the city transit this facility, making it highly representative of Lusaka at large. Additionally, we were able to combine multiple data elements on the same individual: clinical presentation, molecular testing, geography, and viral lineage, to provide an overall picture of the impact of the COVID-19 pandemic in Lusaka during these periods.

Limitations include that there was a three-month gap in surveillance between Rounds 1 and 2, and that Round 2 ended prior to the resolution of Wave 3 from the Delta variant. These surveillance periods were defined by funding for the research, not by knowledge of COVID-19 prevalence. Our adjudications were necessarily limited by the completeness of clinical data for the facility deaths and by the accuracy of recall from non-medical persons through the verbal autopsy for community deaths. That could lead to misclassification during our adjudications. Our data describe prevalence but cannot provide a case fatality rate since we lack concurrent incidence data for the general population. Lastly, while Round 2 data confirm identify a high proportion of pediatric deaths with COVID-19 and again show that gastrointestinal symptoms are common in children <5 years, we are very reluctant to inferring causality in this age group. In our judgement, resolving this question requires a higher gold standard, such as PCR coupled with histopathology. Our data do not address the impact of COVID-19 in terms of a case or infection fatality rate, but this question is being examined in an ongoing excess mortality analysis to be published separately.

The most challenging aspect of this work was inferring the causal role of COVID-19 in each death. We note that the clinical definitions applied during adjudication are themselves of limited precision. However, these are the same definitions we had used in our original report in The BMJ 2021, and we believe our approach here is defensible for several reasons. First, it is supported by guidance from the US CDC: given a patient who presented with respiratory distress and fever and died with a positive PCR test for COVID-19, it is no great leap of faith to infer that the death was probably due to COVID-19. Second, we had conducted minimally invasive tissue sampling on a subset of decedents who had been identified with COVID-19 in life. As previously published, the pathology from these individuals was consistent with known patterns of COVID-19 disease, with evidence of lung and other organ dysfunction.[26] Additionally, we have shown that viral loads were higher among individuals whose deaths were deemed 'probably' due to COVID-19 than those deemed 'probably not' due to COVID-19. And we demonstrated that the ratio of weak to strong PCR signals is age dependent, with much higher ratios in those children and adolescents than older age groups. Combined, these results argue that COVID-19 was a causal factor for deaths in older individuals and more likely a co-incidental finding in those ≤19 years. Causality is far less likely for those deemed 'possibly' due to COVID-19. But such cases comprised a small proportion of all COVID-19 positive deaths in this analysis.

Conclusions

COVID-19 was highly prevalent in the post-mortem setting in Lusaka during the first half of 2021 reaching ~90% of deceased individuals during peak periods of transmission and 30% prevalence over the six months of observation. Despite increased rates of antemortem testing for COVID-19, there was little commensurate increase in

For peer review only - http://bmjopen.bmj.com/site/about/guidelines.xhtml

antemortem testing for those who died in the community, who constituted a 4:1 majority of COVID-19+ deaths. Overall, only about 10% of COVID-19+ decedents had been identified in life, yielding a ~ten-fold undercount. Considering recent reports demonstrating examples of underreporting of COVID-19 elsewhere, including inferential methods from excess mortality analyses,[9-12 34-39] we believe that our results are more likely typical than exceptional. If so, COVID-19's impact in Africa has been underestimated.

ACKNOWLEDGEMENTS

We wish to thank our team at the Bill & Melinda Gates Foundation for their support and encouragement: Prachi Vora, Padmini Srikanthia, and Keith Klugman.

For peer review only

FIGURE LEGENDS

Figure 1. Enrolled decedents and COVID-19+ deaths by 5-year age strata

Each stacked bar in the histogram shows the total number of enrolled decedents within each age stratum. The portion in yellow represents those who tested positive by PCR (Ct <40) for COVID-19 and those in grey those who tested negative (Ct ≥40). The % of positives is indicated at the top of each column. The totals have been adjusted to account for differing enrolment ratios.

Figure 2. Proportion of COVID-19+ deaths and viral lineages/variants, by time and geography

Panel A. The histograms represent the total number of COVID-19 positive deaths identified during the two rounds of surveillance. The gap from October 2020 to January 2021 represents the end and start of funding cycles. It is apparent that there were at least three waves of transmission in 2020-2021. Since our enrollment strategy shifted between Round 1 and Round 2, we have adjusted the proportions for the Round 2 data by weighting to account for the different enrolment ratios applied by age and setting.

Panel B. Combing the samples corresponding to each of the three waves, and the period in between waves 2 and 3, we clustered the viral lineages as shown on the top half of the figure. As can be seen, wave 1 was dominated by lineage AE.1, wave 2 by the 'Beta' variant, and wave 3 by the 'Delta' variant. For these data, we present the results without adjustments for weighting.

Panel C. Over these same time intervals, we summarize the distribution of cases indexed against total population within the major city wards of Lusaka. The colors correspond to a heat map defined by the union of 'total deaths' in pink and 'COVID-19+' deaths in blue, and therefore provide an indexed metric of COVID-19 deaths against all deaths. Those with darker shading indicate that COVID-19 deaths were disproportionately high relative to all deaths; those that are lightly shaded or white are where COVID-19 deaths were disproportionately low, relative to total deaths. As can be seen, the highest concentration of cases clustered in the peripheral areas of the city, which correspond to the poorest and most densely populated parts of the city. Whereas the wealthier sections, clustering in the middle of Lusaka, were relatively spared. Round 2 results have also been adjusted for enrollment ratios as in Panel A.

For peer review only - http://bmjopen.bmj.com/site/about/guidelines.xhtml

DECLARATIONS

Dissemination of results

Round 2 data were presented to members of the Zambian Ministry of Health and the Zambian Medical Association in July 2021, and the final manuscript was shared with the Zambian National Health Research Authority (NHRA) prior to submission. The results have been presented to our team at the Bill & Melinda Gates Foundation and to representatives from the US CDC and the Swiss Tropical Medicine Institute. These data have been shared with a modeling team at the Imperial College London to support our ongoing excess mortality analyses. Data from Round 1 were published previously in the BMJ.

Funding

The original ZPRIME study and this COVID-19 expansion were made possible through the generous support of the Bill & Melinda Gates Foundation (OPP 1163027). The funders had no role in designing the study; in the collection and analysis of data; or in the decision to submit the article for publication.

Data sharing

Data are available upon reasonable request

Community engagement

Due to the time constraints around the pandemic, we did not engage the community on this project.

Data provenance

The lead authors (CJG and LM) affirm that the manuscript is an honest, accurate, and transparent account of the study being reported; that no important aspects of the study have been omitted; and that any discrepancies from the study as originally planned (and, if relevant, registered) have been explained.

Manuscript contributions

CJG was the principle investigator, conceived the project, secured the funding, assisted with the protocol and led on writing the manuscript; LM was the Zambian PI, assisted with protocol and tool development, and oversaw the field team and study implementation; WBM was the study statistician, developed the analysis plan and conducted the analysis; GK was the technical lead for the PCR lab in Lusaka; RP was the project manager and a research fellow, developed the study proposal, oversaw study management, and contributed to key aspects of data analysis and presentation; LE was a research fellow and contributed to key aspects of data analysis and presentation; RN, Chilufya Chikoti (CC#1) and BY were the PCR technicians for our lab;

For peer review only

SC, LP, GM, DN were the field data collection team under team leaders BN and Charles Chimoga (CC#2, unrelated to CC#1), who conducted grief counseling for the families, obtained consent, and collected all of the clinical data and the biological samples: LF supervised creation of the RedCAP data collection tools and oversaw data management, cleaning and retention; RL contributed to data interpretation and analysis; JL provided information technology support to the field team; BK, JM, BM, and WM conducted the genetic sequencing analyses under the supervision of DB and ES; ZM was the PCR lab manager; NP was a research fellow who supported some of the analyses; MM and NS contributed to the genetic sequencing analyses; and DMT was our senior co-investigator who contributed to data analysis and interpretation.

Ethical review:

The research was conducted with the approval of the ethical review boards from Boston University (reference number H-36469) and the University of Zambia (reference number 015-11-18); written informed consent was obtained from the next of kin or family representative in all cases.

Conflict of Interest:

All authors declare that they have no conflicts of interest relevant to this publication.

For peer review only

REFERENCES

1. African C-CCOSI. Patient care and clinical outcomes for patients with COVID-19 infection admitted to African high-care or intensive care units (ACCCOS): a multicentre, prospective, observational cohort study. *Lancet* 2021;397(10288):1885-94. doi: 10.1016/S0140-6736(21)00441-4 [published Online First: 2021/05/24]
2. Mwananyanda L, Gill CJ, MacLeod W, et al. Covid-19 deaths in Africa: prospective systematic postmortem surveillance study. *BMJ* 2021;372:n334. doi: 10.1136/bmj.n334 [published Online First: 2021/02/19]
3. Zerfu TA, Tareke AA. What could be the potential reasons for relatively low coronavirus disease 2019 (COVID-19) fatality rates in Africa? The case for Ethiopia. *J Glob Health* 2021;11:03057. doi: 10.7189/jogh.11.03057 [published Online First: 2021/04/22]
4. Musa HH, Musa TH, Musa IH, et al. Addressing Africa's pandemic puzzle: Perspectives on COVID-19 transmission and mortality in sub-Saharan Africa. *Int J Infect Dis* 2021;102:483-88. doi: 10.1016/j.ijid.2020.09.1456 [published Online First: 2020/10/04]
5. Lawal Y. Africa's low COVID-19 mortality rate: A paradox? *Int J Infect Dis* 2021;102:118-22. doi: 10.1016/j.ijid.2020.10.038 [published Online First: 2020/10/20]
6. Bamgboye EL, Omiye JA, Afolaranmi OJ, et al. COVID-19 Pandemic: Is Africa Different? *J Natl Med Assoc* 2021;113(3):324-35. doi: 10.1016/j.jnma.2020.10.001 [published Online First: 2020/11/07]
7. Adams J, MacKenzie MJ, Amegah AK, et al. The Conundrum of Low COVID-19 Mortality Burden in sub-Saharan Africa: Myth or Reality? *Glob Health Sci Pract* 2021;9(3):433-43. doi: 10.9745/GHSP-D-21-00172 [published Online First: 2021/10/02]
8. Ntoumi F, Velavan TP. COVID-19 in Africa: between hope and reality. *Lancet Infect Dis* 2020 doi: 10.1016/S1473-3099(20)30465-5 [published Online First: 2020/06/20]
9. Warsame A, Bashiir F, Freemantle T, et al. Excess mortality during the COVID-19 pandemic: a geospatial and statistical analysis in Mogadishu, Somalia. *Int J Infect Dis* 2021;113:190-99. doi: 10.1016/j.ijid.2021.09.049 [published Online First: 2021/09/28]
10. Watson OJ, Alhaffar M, Mehchy Z, et al. Leveraging community mortality indicators to infer COVID-19 mortality and transmission dynamics in Damascus, Syria. *Nat Commun* 2021;12(1):2394. doi: 10.1038/s41467-021-22474-9 [published Online First: 2021/04/24]
11. Kogan NE, Gantt S, Swerdlow D, et al. Leveraging Serosurveillance and Postmortem Surveillance to Quantify the Impact of COVID-19 in Africa. *Clin Infect Dis* 2022 doi: 10.1093/cid/ciac797 [published Online First: 20221005]
12. Lewis HC, Ware H, Whelan M, et al. SARS-CoV-2 infection in Africa: a systematic review and meta-analysis of standardised seroprevalence studies, from January

For peer review only

For peer review only - http://bmjopen.bmj.com/site/about/guidelines.xhtml

2020 to December 2021. *BMJ Glob Health* 2022;7(8) doi: 10.1136/bmjgh-2022-008793

13. Gill CJ, Mwananyanda L, MacLeod WB, et al. Infant deaths from respiratory syncytial virus in Lusaka, Zambia from the ZPRIME study: a 3-year, systematic, post-mortem surveillance project. *Lancet Glob Health* 2022;10(2):e269-e77. doi: 10.1016/S2214-109X(21)00518-0 [published Online First: 2022/01/23]
14. Murphy C, MacLeod WB, Forman LS, et al. Risk Factors for Respiratory Syncytial Virus-Associated Community Deaths in Zambian Infants. *Clin Infect Dis* 2021;73(Suppl_3):S187-S92. doi: 10.1093/cid/ciab453 [published Online First: 2021/09/03]
15. Forman LS, Macleod W, Mwananyanda L, et al. Association of Respiratory Syncytial Virus Infection and Underlying Risk Factors for Death Among Young Infants Who Died at University Teaching Hospital, Lusaka Zambia. *Clin Infect Dis* 2021;73(Suppl_3):S180-S86. doi: 10.1093/cid/ciab466 [published Online First: 2021/09/03]
16. Centers for Disease Control and Prevention. Guidance for certifying deaths due to coronavirus disease 2919 (COVID-19). Vital Statistics Reporting Guidance. Hyattsville, Maryland: National Center for Health Statistics, 2020.
17. Serina P, Riley I, Stewart A, et al. A shortened verbal autopsy instrument for use in routine mortality surveillance systems. *BMC Med* 2015;13:302. doi: 10.1186/s12916-015-0528-8 [published Online First: 2015/12/17]
18. Ramlall V, Thangaraj PM, Meydan C, et al. Immune complement and coagulation dysfunction in adverse outcomes of SARS-CoV-2 infection. *Nat Med* 2020;26(10):1609-15. doi: 10.1038/s41591-020-1021-2 [published Online First: 2020/08/05]
19. Van Horn KG, Audette CD, Sebeck D, Tucker KA. Comparison of the Copan ESwab system with two Amies agar swab transport systems for maintenance of microorganism viability. *J Clin Microbiol* 2008;46(5):1655-8. doi: 10.1128/JCM.02047-07 [published Online First: 2008/03/21]
20. Van Horn KG, Audette CD, Tucker KA, Sebeck D. Comparison of 3 swab transport systems for direct release and recovery of aerobic and anaerobic bacteria. *Diagn Microbiol Infect Dis* 2008;62(4):471-3. doi: 10.1016/j.diagmicrobio.2008.08.004 [published Online First: 2008/09/26]
21. Yang G, Erdman DE, Kodani M, et al. Comparison of commercial systems for extraction of nucleic acids from DNA/RNA respiratory pathogens. *J Virol Methods* 2011;171(1):195-9. doi: 10.1016/j.jviromet.2010.10.024 [published Online First: 2010/11/03]
22. Lu X, Wang L, Sakthivel SK, et al. US CDC Real-Time Reverse Transcription PCR Panel for Detection of Severe Acute Respiratory Syndrome Coronavirus 2. *Emerg Infect Dis* 2020;26(8) doi: 10.3201/eid2608.201246 [published Online First: 2020/05/13]
23. Gill CJ, Gunning CE, MacLeod WB, et al. Asymptomatic Bordetella pertussis infections in a longitudinal cohort of young African infants and their mothers. *Elife* 2021;10 doi: 10.7554/eLife.65663 [published Online First: 2021/06/08]

For peer review only

24. Mwenda M, Saasa N, Sinyange N, et al. Detection of B.1.351 SARS-CoV-2 Variant Strain - Zambia, December 2020. *MMWR Morb Mortal Wkly Rep* 2021;70(8):280-82. doi: 10.15585/mmwr.mm7008e2 [published Online First: 2021/02/26]
25. Simulundu E, Mupeta F, Chanda-Kapata P, et al. First COVID-19 case in Zambia - Comparative phylogenomic analyses of SARS-CoV-2 detected in African countries. *Int J Infect Dis* 2021;102:455-59. doi: 10.1016/j.ijid.2020.09.1480 [published Online First: 2020/10/10]
26. Mudenda V, Mumba C, Pieciak RC, et al. Histopathological Evaluation of Deceased Persons in Lusaka, Zambia With or Without Coronavirus Disease 2019 (COVID-19) Infection: Results Obtained From Minimally Invasive Tissue Sampling. *Clin Infect Dis* 2021;73(Suppl_5):S465-S71. doi: 10.1093/cid/ciab858 [published Online First: 2021/12/16]
27. Scheiblauer H, Filomena A, Nitsche A, et al. Comparative sensitivity evaluation for 122 CE-marked rapid diagnostic tests for SARS-CoV-2 antigen, Germany, September 2020 to April 2021. *Euro Surveill* 2021;26(44) doi: 10.2807/1560-7917.ES.2021.26.44.2100441 [published Online First: 2021/11/06]
28. Lapidot R, Larson Williams A, MacLeod WB, et al. Verbal Autopsies for Out-of-Hospital Infant Deaths in Zambia. *Pediatrics* 2021;147(4) doi: 10.1542/peds.2020-1767 [published Online First: 2021/03/06]
29. Johnson AG, Amin AB, Ali AR, et al. COVID-19 Incidence and Death Rates Among Unvaccinated and Fully Vaccinated Adults with and Without Booster Doses During Periods of Delta and Omicron Variant Emergence - 25 U.S. Jurisdictions, April 4-December 25, 2021. *MMWR Morb Mortal Wkly Rep* 2022;71(4):132-38. doi: 10.15585/mmwr.mm7104e2 [published Online First: 2022/01/28]
30. Shuai H, Chan JF, Hu B, et al. Attenuated replication and pathogenicity of SARS-CoV-2 B.1.1.529 Omicron. *Nature* 2022 doi: 10.1038/s41586-022-04442-5 [published Online First: 2022/01/22]
31. Fisman DN, Tuite AR. Evaluation of the relative virulence of novel SARS-CoV-2 variants: a retrospective cohort study in Ontario, Canada. *Cmaj* 2021;193(42):E1619-E25. doi: 10.1503/cmaj.211248 [published Online First: 2021/10/07]
32. Zhang J, Xiao T, Cai Y, et al. Membrane fusion and immune evasion by the spike protein of SARS-CoV-2 Delta variant. *Science* 2021;374(6573):1353-60. doi: 10.1126/science.abl9463 [published Online First: 2021/10/27]
33. Tao K, Tzou PL, Nouhin J, et al. The biological and clinical significance of emerging SARS-CoV-2 variants. *Nat Rev Genet* 2021;22(12):757-73. doi: 10.1038/s41576-021-00408-x [published Online First: 2021/09/19]
34. Walker PGT, Whittaker C, Watson OJ, et al. The impact of COVID-19 and strategies for mitigation and suppression in low- and middle-income countries. *Science* 2020;369(6502):413-22. doi: 10.1126/science.abc0035 [published Online First: 2020/06/14]
35. Russell TW, Golding N, Hellewell J, et al. Reconstructing the early global dynamics of under-ascertained COVID-19 cases and infections. *BMC Med* 2020;18(1):332. doi: 10.1186/s12916-020-01790-9 [published Online First: 2020/10/23]
36. Koum Besson ES, Norris A, Bin Ghouth AS, et al. Excess mortality during the COVID-19 pandemic: a geospatial and statistical analysis in Aden governorate,

For peer review only

Yemen. *BMJ Glob Health* 2021;6(3) doi: 10.1136/bmjgh-2020-004564 [published Online First: 2021/03/25]

37. Karlinsky A, Kobak D. Tracking excess mortality across countries during the COVID-19 pandemic with the World Mortality Dataset. *Elife* 2021;10 doi: 10.7554/eLife.69336 [published Online First: 2021/07/01]
38. Alves THE, de Souza TA, Silva SA, Ramos NA, de Oliveira SV. Underreporting of Death by COVID-19 in Brazil's Second Most Populous State. *Front Public Health* 2020;8:578645. doi: 10.3389/fpubh.2020.578645 [published Online First: 2021/01/02]
39. Van Gordon MM, Mwananyanda L, Gill CJ, McCarthy KA. Regional comparisons of COVID reporting rates, burden, and mortality age-structure using auxiliary data sources. *MedRxiv (preprint)* 2021 doi: 2021.08.18.212622482021

For peer review only

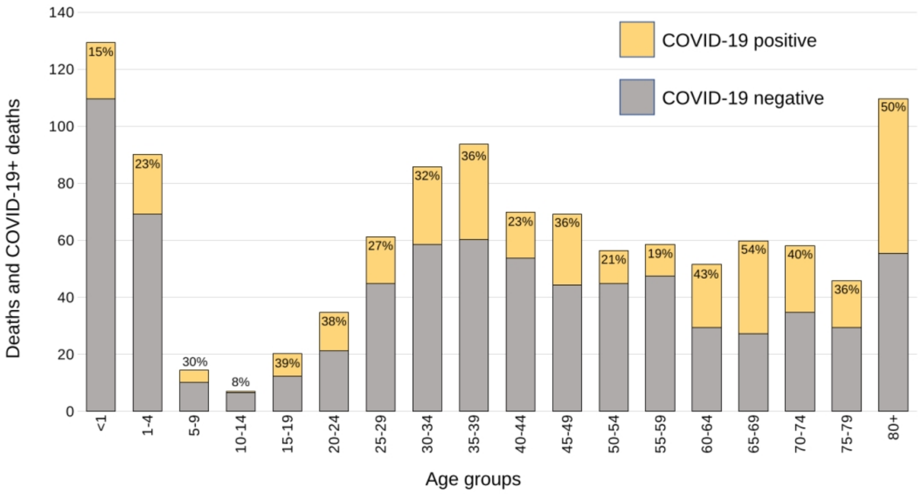

Figure 1. Enrolled decedents and COVID-19+ deaths by 5-year age strata

37058x20828mm (1 x 1 DPI)

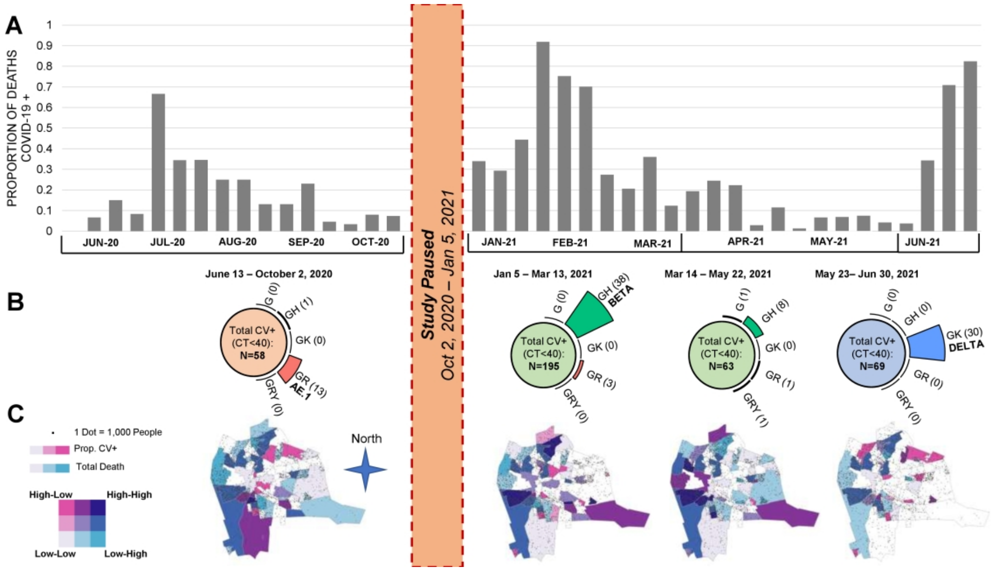

Figure 2. Proportion of COVID-19+ deaths and viral lineages/variants, by time and geography

338x190mm (300 x 300 DPI)

SUPPLEMENTARY DATA

Table S1. Proportion of the deceased who had been tested antemortem, by setting

	Facility	Community	All deaths
Tested antemortem	47.4% (272)	1.8% (10)	282
Not tested antemortem	52.6% (302)	97.2% (534)	836
Total	574	544	1,118

Note: We have elected not to conduct a detailed analysis of the concordance between the off study antemortem (clinical care) and postmortem (research) testing. Our team had no role in how those off study samples were collected, what swabs were used, how staff were trained, what test kits were used over time, what quality control measures were used, and so forth. Hence, we have no provenance over the non-study data and do not vouch for their accuracy.

With that caveat, we identified 35 individuals for whom a positive test result was recorded or reported (25 facility and 10 community deaths). Obviously, this is far fewer than what we observed in our postmortem testing. For the reasons outlined above, we can only hypothesize explanations for this discrepancy. Yet, the simplest explanation is plausible: most of the clinical samples were evaluated using rapid antigen kits, which are far less sensitive than PCR.

Please also note that this Table does not account for the different enrollment ratios between the facility and community deaths, which will under-represent the community deaths by three-fold.

For peer review only

Table S2. Proportion of the deceased who tested positive for CV19 by study month

	Ct>=40 (Neg)	Ct<40 (Pos)	Total	Ct>=40 (Neg)	Ct<40 (Pos)	Total
**Month**	**Unweighted**			**Weighted**		
January	77<br>47.8%	84<br>52.2%	161	108.1<br>49.4%	110.8<br>50.6%	218.8
February	118<br>57.0%	89<br>43.0%	207	118.2<br>57.1%	88.9<br>42.9%	207.2
March	189<br>77.5%	55<br>22.5%	244	173.1<br>76.7%	52.7<br>23.4%	225.8
April	216<br>91.5%	20<br>8.5%	236	176.8<br>92.2%	14.9<br>7.8%	191.7
May	154<br>89.0%	19<br>11.0%	173	154.4<br>92.1%	13.3<br>7.9%	167.7
June	37<br>38.1%	60<br>61.9%	97	29.3<br>27.4%	77.7<br>72.6%	107.0
Total	791<br>70.8%	327<br>29.2%	1,118	759.9<br>68.0%	358.4<br>32.0%	1,118.3<br>100.0%

Row results are N (Top) and % (Bottom) in each cell.

For peer review only

Table S3. Clinical presentations of pediatric CV19 positive deaths

Table S3a. Clinical presentation of COVID-19+ facility deaths in children aged 0-19 years

No.	Age in years	Respiratory Symptoms	Gastrointestinal Symptoms	Fever	Cause of Death*
1	<1	difficulty breathing	diarrhea	X	sepsis
2	<1	cough		X	pneumonia
3	<1	cough, difficulty breathing, shortness of breath	diarrhea, vomiting	X	pneumonia
4	<1	difficulty breathing			sepsis
5	<1	cough, difficulty breathing		X	COVID-19 pneumonia
6	<1	difficulty breathing			sepsis
7	<1	difficulty breathing			pneumonia
8	<1	difficulty breathing			sepsis
9	<1	runny nose			sepsis
10	<1	difficulty breathing			sepsis
11	<1	cough	vomiting		sepsis
12	<1	difficulty breathing		X	sepsis
13	<1		vomiting	X	sepsis
14	<1			X	sepsis
15	<1			X	sepsis
16	<1				sepsis
17	<1	difficulty breathing, shortness of breath		X	sepsis
18	<1			X	sepsis
19	<1			X	sepsis
20	<1	cough, difficulty breathing		X	sepsis
21	<1	difficulty breathing			sepsis
22	<1				hypothermia
23	<1			X	sepsis
24	<1	difficulty breathing			pneumonia
25	<1	cough, difficulty breathing		X	pneumonia
26	1-5	shortness of breath		X	sepsis
27	1-5		diarrhea, vomiting		diarrhea
28	1-5				dehydration
29	1-5		diarrhea	X	malnutrition
30	1-5		diarrhea	X	sepsis
31	1-5			X	respiratory failure
32	6-10				diarrhea
33	6-10	difficulty breathing			sepsis
34	11-15				intracranial abscess

* Cause of death from the official death certificate completed by the UTH medical examiner.

For peer review only

Table S3b. Clinical presentation of COVID-19+ community deaths in children aged 0-19 years

No.	Age in years	Respiratory Symptoms	Gastrointestinal Symptoms	Fever
1	<1	difficulty breathing		
2	<1	cough		X
3	<1	cough, difficulty breathing	vomiting	
4	<1		vomiting	
5	<1			X
6	<1		diarrhea, vomiting	X
7	<1	cough, difficulty breathing, fast breathing, shortness of breath		X
8	<1			
9	<1			
10	<1	cough, fast breathing		X
11	<1		diarrhea, vomiting	X
12	<1	cough, difficulty breathing	diarrhea	X
13	1-5	cough		X
14	1-5		diarrhea, vomiting	
15	1-5		diarrhea, vomiting	X
16	1-5		diarrhea	
17	1-5		diarrhea, vomiting	X
18	1-5			
19	1-5			X
20	1-5		diarrhea	
21	1-5	cough	diarrhea	
22	1-5			
23	1-5	cough, difficulty breathing		
24	6-10			
25	6-10		diarrhea	
26	16-19	difficulty breathing		
27	16-19	cough, difficulty breathing		
28	16-19			X
29	16-19			
30	16-19			

We do not include a cause of death for the community deaths. A death certificate is still issued for each case, but these are not based on information from medical providers and are not considered to be accurate.

For peer review only

Supplementary Table S4. The relationship between age and PCR results and viral load as measured through cycle threshold values (Ct).

Age at death (years)	Ct≥40 (Neg)	Weaker (30≤Ct<40)	Strong-est (Ct<30)	Ratio of Weaker to Strongest	Ct≥40 (Neg)	Weaker (30≤Ct<40)	Strong-est (Ct<30)	Ratio of Weaker to Strongest
	Unweighted				Weighted			
0-19	304	54	9	6.0	203.4	45.8	6.9	6.6
	82.8	14.7	2.5		79.4	17.9	2.7	
20-39	158	55	24	2.3	184.2	62.3	27.7	2.3
	66.7	23.2	10.1		67.2	22.7	10.1	
40-59	171	36	24	1.7	190.1	39.4	23.4	1.7
	74.0	15.6	10.4		75.2	15.6	9.3	
60-79	107	45	31	1.4	119.8	54.8	38.9	1.4
	58.5	24.6	16.9		56.1	25.7	18.2	
80-99	42	19	19	1.0	55.4	26.1	25.0	1.0
	52.5	23.8	23.8		52.0	24.5	23.5	
100+	0	1	1	1.0	0	1.6	1.6	1.0
	0.0	50.0	50.0		0.0	50.0	50.0	
Total	782	210	108	1.9	752.9	230.0	123.5	1.9

*4 had missing age data and 12 had no Ct value (tested antemortem with rapid test).

Row results are N (Top) and % (Bottom) in each cell.

Within each age strata we plot the proportion of decedents with a ‘negative’ test, defined as Ct >40, or a positive test, stratified by Ct strata. Ct is the inverse of the viral load, hence low Ct equals a stronger PCR result and a higher vial load. What we observe is an age dependent effect on the ratio of weaker to strongest PCR strata. Among younger individuals ≤19 years, the ratio of weaker to strongest PCR results was 6.6:1, after weighting. This ratio declines as a function of increasing age. If we assume that viral load is reflective of pathology, then the higher ratios seen in the youngest group indicate a greater proportion of cases where COVID-19 was a co-incidental finding, whereas those in the older age groups, indicate that COVID-19 was more likely to have been a causal factor in those deaths.

For peer review only

Figure S1. Comparison of death by age distributions for the total deceased cohort, the enrolled cohort, and the CV19 positive enrolled cohort.

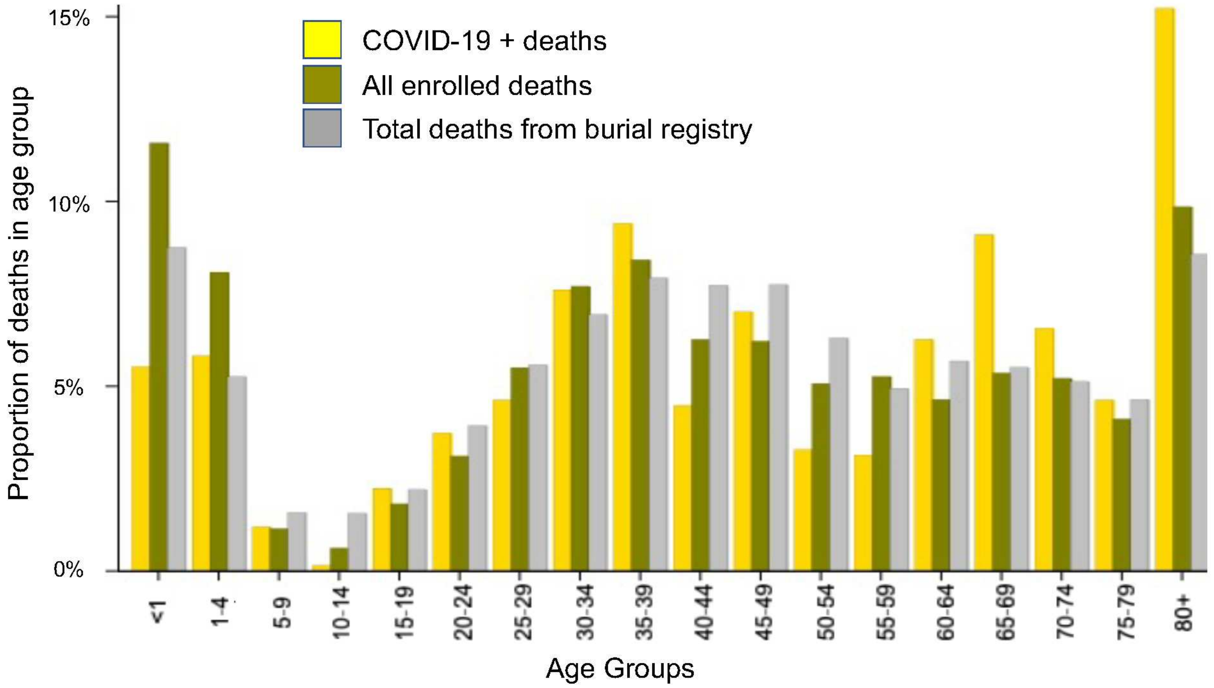

Supplementary Figure S1. Comparison of death by age distributions for the total deceased cohort, the enrolled cohort, and the COVID-19 positive enrolled cohort.

Data for the total death cohort were from Lusaka's burial registry logs maintained by the Zambian Ministry of Health. From each entry, we extracted data of death, age, and sex. We then extracted those deaths that corresponded to the periods of surveillance to capture the age by death distribution for the total cohort (enrolled and unenrolled). The second two sets correspond to the total enrolled cohort (N=1,118 person) and the subset who were COVID-19+ (N=327).

Figure S2. Distribution of cycle threshold values for PCR results targeting the N1 and N2 nucleocapsid proteins.

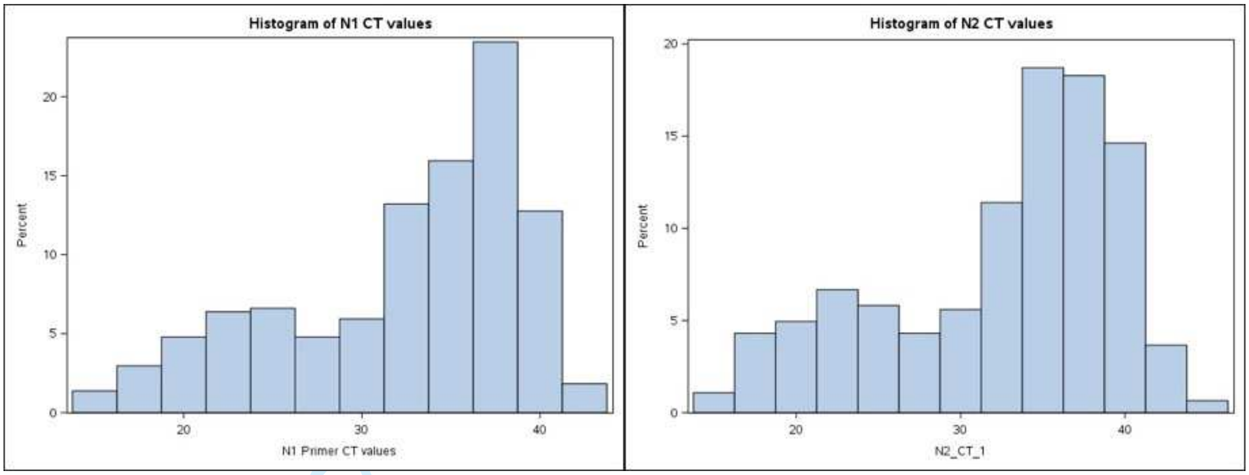

Supplementary Figure S2. Distribution of cycle threshold values for the N1 and N2 PCR reactions

The median Ct results were 32 and 33, respectively for the N1 and N2 targets. Results for detections between >40 and 45 are noted in text but are not included in any of our summary results otherwise.

Supplementary Figure S3

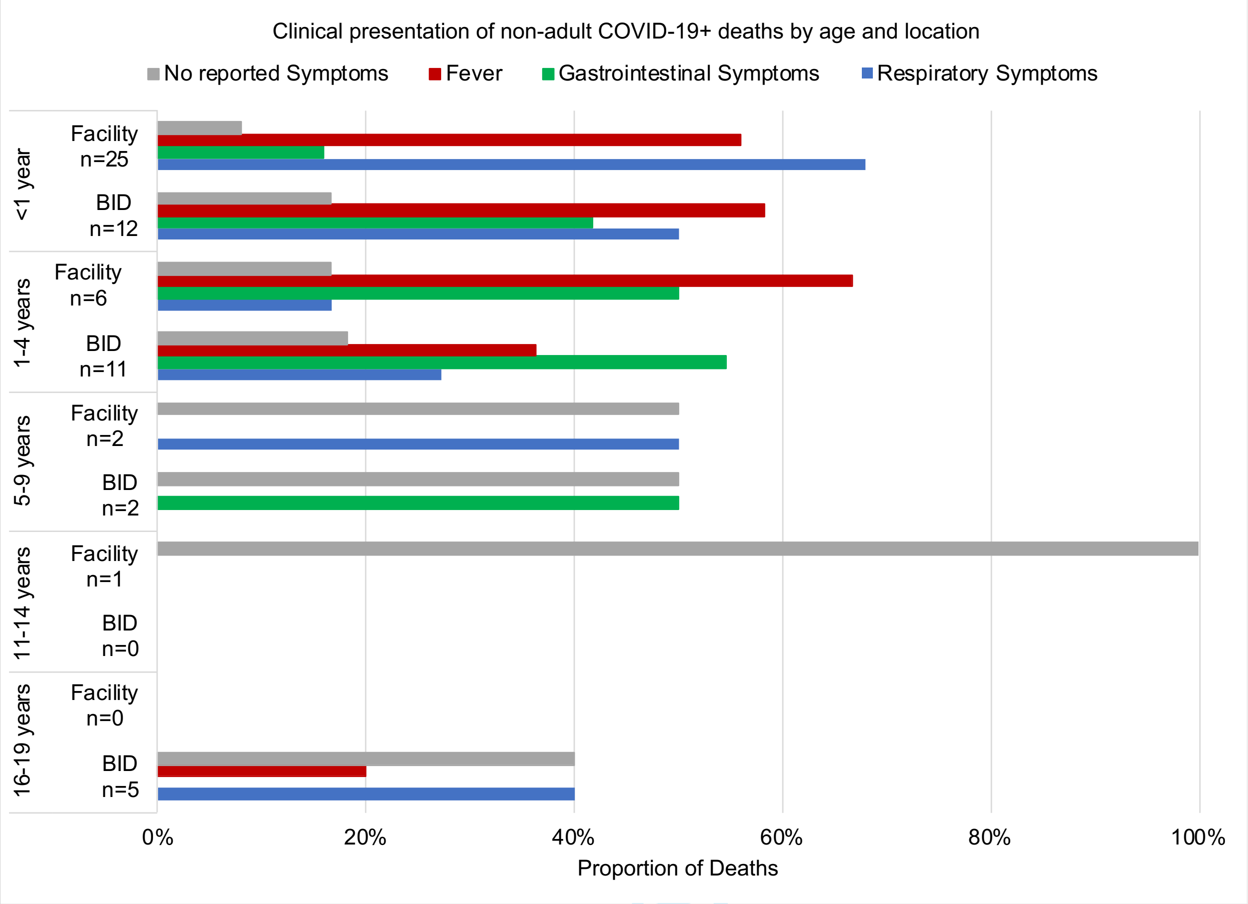

Supplementary Figure S3. Clinical presentations among COVID-19 positive children ≤19 years

For this analysis, we extracted the data about clinical presentation of each case from the medical charts (facility deaths) or verbal autopsy data (community deaths). We then clustered these syndromically into those that were ‘respiratory’ vs. ‘gastrointestinal’ vs. ‘other’. Since ‘fever’ could occur in any of these, or by itself, we summarized this separately. As can be seen, the syndromic presentation of COVID-19+ infants <1 is distinct from that in older children. In the infants, we see a high proportion with gastrointestinal complaints and a relative paucity with respiratory disease. In the older children, this pattern reverses, and in adolescents the pattern is consistent with that seen in adults, i.e., various combinations of respiratory symptoms, often accompanied by fever. Since this is an analysis by individual, there was no need to make weighting adjustments to account for enrollment ratios.

STROBE Statement—Checklist of items that should be included in reports of ***cross-sectional studies***

	Item No	Recommendation
**Title and abstract**	1	(*a*) Indicate the study's design with a commonly used term in the title or the abstract***Done pg 1
		(*b*) Provide in the abstract an informative and balanced summary of what was done and what was found***Done
**Introduction**		
Background/rationale	2	Explain the scientific background and rationale for the investigation being reported*** Done
Objectives	3	State specific objectives, including any prespecified hypotheses*** Done
**Methods**		
Study design	4	Present key elements of study design early in the paper*** Done
Setting	5	Describe the setting, locations, and relevant dates, including periods of recruitment, exposure, follow-up, and data collection*** Done
Participants	6	(*a*) Give the eligibility criteria, and the sources and methods of selection of participants*** Done
Variables	7	Clearly define all outcomes, exposures, predictors, potential confounders, and effect modifiers. Give diagnostic criteria, if applicable*** Done
Data sources/ measurement	8*	For each variable of interest, give sources of data and details of methods of assessment (measurement). Describe comparability of assessment methods if there is more than one group*** Done pg 3
Bias	9	Describe any efforts to address potential sources of bias*** Done pg 5
Study size	10	Explain how the study size was arrived at***Done pg 7
Quantitative variables	11	Explain how quantitative variables were handled in the analyses. If applicable, describe which groupings were chosen and why*** not applicable – we did not do modeling
Statistical methods	12	(*a*) Describe all statistical methods, including those used to control for confounding*** Not applicable to this study design
		(*b*) Describe any methods used to examine subgroups and interactions*** Not applicable, there was no modelling in this analysis
		(*c*) Explain how missing data were addressed*** We did not use imputation so this did not apply.
		(*d*) If applicable, describe analytical methods taking account of sampling strategy*** Not applicable
		(*e*) Describe any sensitivity analyses*** Not applicable
**Results**		
Participants	13*	(a) Report numbers of individuals at each stage of study—eg numbers potentially eligible, examined for eligibility, confirmed eligible, included in the study, completing follow-up, and analysed*** Not applicable -this was a cross sectional study, so attrition does not apply
		(b) Give reasons for non-participation at each stage*** Not applicable
		(c) Consider use of a flow diagram*** Also necessary. This was a prevalence study conducted among postmortem participants. There is no 'flow' to report under this methodology
Descriptive data	14*	(a) Give characteristics of study participants (eg demographic, clinical, social) and information on exposures and potential confounders*** Done pg10

For peer review only

For peer review only - http://bmjopen.bmj.com/site/about/guidelines.xhtml

		(b) Indicate number of participants with missing data for each variable of interest*** Not applicable
Outcome data	15*	Report numbers of outcome events or summary measures*** Done pg 9 and actually throughout the results
Main results	16	(*a*) Give unadjusted estimates and, if applicable, confounder-adjusted estimates and their precision (eg, 95% confidence interval). Make clear which confounders were adjusted for and why they were included*** Not applicable. We did not do modelling.
		(*b*) Report category boundaries when continuous variables were categorized***Not applicable
		(*c*) If relevant, consider translating estimates of relative risk into absolute risk for a meaningful time period*** Not applicable. We do not report on relative risks.
Other analyses	17	Report other analyses done—eg analyses of subgroups and interactions, and sensitivity analyses*** not applicable
**Discussion**		
Key results	18	Summarise key results with reference to study objectives*** Done pg 16
Limitations	19	Discuss limitations of the study, taking into account sources of potential bias or imprecision. Discuss both direction and magnitude of any potential bias*** Done pgs 17-18
Interpretation	20	Give a cautious overall interpretation of results considering objectives, limitations, multiplicity of analyses, results from similar studies, and other relevant evidence*** Done. pg 18
Generalisability	21	Discuss the generalisability (external validity) of the study results*** Done pg 18
**Other information**		
Funding	22	Give the source of funding and the role of the funders for the present study and, if applicable, for the original study on which the present article is based*** Done pg 20

*Give information separately for exposed and unexposed groups.

Note: An Explanation and Elaboration article discusses each checklist item and gives methodological background and published examples of transparent reporting. The STROBE checklist is best used in conjunction with this article (freely available on the Web sites of PLoS Medicine at http://www.plosmedicine.org/, Annals of Internal Medicine at http://www.annals.org/, and Epidemiology at http://www.epidem.com/). Information on the STROBE Initiative is available at www.strobe-statement.org.

For peer review only

For peer review only - http://bmjopen.bmj.com/site/about/guidelines.xhtml
